# Contra‐Diffusion Engineering of Single‐Atom Catalytic Interlayers Enables Reversible Sulfur Redox Chemistry

**DOI:** 10.1002/anie.7009531

**Published:** 2026-04-27

**Authors:** Yan‐Jhang Chen, Tsung‐I. Yeh, Chia‐Yu Chang, Wei‐Ming Huang, Jing‐Yu Li, Mohamed Gamal Mohamed, Shiao‐Wei Kuo, Bing‐Joe Hwang, Yun‐Sheng Ye

**Affiliations:** ^1^ Department of Materials and Optoelectronic Science Center of Crystal Research National Sun Yat‐Sen University Kaohsiung Taiwan; ^2^ Department of Chemical Engineering National Taiwan University of Science and Technology Taipei Taiwan; ^3^ Sustainable Electrochemical Energy Development Center (SEED Center) National Taiwan University of Science and Technology Taipei Taiwan; ^4^ Graduate Institute of Applied Science and Technology National Taiwan University of Science and Technology Taipei Taiwan

**Keywords:** contra‐diffusion synthesis, lithium–sulfur batteries, metal–organic frameworks, polysulfide conversion, single‐atom catalysts

## Abstract

Achieving durable lithium–sulfur batteries with minimal catalyst loading remains challenging, particularly for interlayer designs where catalytic efficiency is often compromised by nonuniform active‐site utilization. Here we demonstrate that diffusion‐regulated precursor growth enables the construction of atomically dispersed Co–N_x_ catalytic sites within a freestanding aramid nanofiber‐derived carbon interlayer. By synchronizing the bidirectional diffusion of metal ions and ligands, this process enforces spatially confined nucleation and homogeneous precursor evolution, yielding a uniformly accessible single‐atom catalytic architecture while preserving the intrinsic fibrous conduction network. The resulting interlayer simultaneously enhances polysulfide anchoring, accelerates bidirectional sulfur redox kinetics, and regulates Li_2_S nucleation and dissolution, as directly revealed by in situ Raman spectroscopy and electrochemical analyses. As a consequence, the system delivers exceptional cycling stability under high‐rate operation despite a low Co loading, highlighting the importance of diffusion‐regulated catalytic architectures for efficient sulfur redox regulation in lithium–sulfur batteries.

## Graphical abstract

A contra‐diffusion strategy is introduced to grow uniformly distributed Co–N_x_ single‐atom catalysts (CoSAs) within a carbonized aramid nanofiber (CANF) interlayer. The resulting CD‐CANF enables efficient polysulfide regulation, low metal loading, and exceptional long‐term cycling stability in lithium–sulfur batteries.

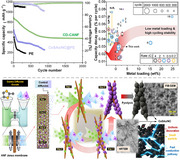

## 1 Introduction

Rechargeable lithium–sulfur (Li–S) batteries have attracted intensive research interest owing to their remarkable theoretical energy density (2600 Wh kg^−1^), high sulfur abundance, and intrinsic sustainability advantages over transition‐metal‐based cathode systems [1]. Despite these appealing features, the practical deployment of Li–S batteries is severely hindered by two intrinsic challenges: the dissolution and uncontrolled migration of lithium polysulfides (LiPSs) across the separator, and the intrinsically sluggish redox conversion kinetics between S_8_, Li_2_S_n_ intermediates, and Li_2_S. The combination of these effects leads to rapid capacity fade, poor Coulombic efficiency, and limited rate capability. Therefore, constructing functional interlayers capable of simultaneously suppressing LiPS shuttling and regulating multistep sulfur redox reactions has emerged as an effective strategy toward high‐performance Li–S systems [2]. In particular, designing catalytic interlayers with well‐defined active‐site distribution and continuous ion‐transport pathways is increasingly recognized as a critical factor for achieving efficient sulfur conversion and long‐term cycling stability.

To address the sluggish sulfur redox kinetics, extensive efforts have focused on introducing electrocatalytic centers into the sulfur cathode environment and separator interfaces. Transition‐metal‐based catalysts, including metal oxides, sulfides, carbides, and heteroatom‐doped carbons, have demonstrated the ability to accelerate the nucleation and decomposition of Li_2_S species by lowering the activation barriers of the multistep conversion reactions. Among these catalysts, single‐atom catalysts (SACs) have emerged as a particularly powerful class owing to their maximized atomic utilization, well‐defined coordination environments, and strong chemisorption capability toward polysulfide intermediates. The isolated metal‐N_x_ sites in SACs can effectively modulate the adsorption strength and reaction pathway of LiPSs, thereby enabling both improved reaction kinetics and suppressed shuttle behavior [3, 4]. However, despite these advantages, achieving homogeneous spatial distribution of SACs within practical battery architectures remains challenging. In many reported systems, catalytic sites are either confined within cathode hosts or deposited nonuniformly on separator, leading to uneven catalytic utilization and limited accessibility of active sites. Therefore, constructing architectures that can simultaneously ensure uniform single‐atom dispersion and continuous ion‐transport pathway is crucial for maximizing catalytic efficiency in Li–S batteries [5, 6].

Metal–organic frameworks (MOFs), particularly zeolitic imidazolate frameworks (ZIFs), have been widely explored as precursors for constructing atomically dispersed metal‐N_x_ catalytic sites [7]. Their well‐defined metal–ligand coordination, tunable composition, and high‐density nitrogen environments provide a favorable structural platform for stabilizing isolated metal atoms after pyrolysis [8, 9]. In Zn–Co bimetallic ZIFs, the presence of volatile Zn plays an additional role in preventing metal aggregation by creating abundant vacancies during thermal treatment, thereby facilitating the formation of Co–N_4_ single‐atom centers. However, conventional MOF synthesis strategies, such as solution‐phase mixing, solvothermal growth, or direct impregnation, typically lead to uncontrolled homogeneous nucleation in solution, resulting in poor control over precursor deposition on solid substrates. These limitations make it difficult to achieve spatially confined precursor deposition or uniform metal distribution within three‐dimensional host architectures, which is essential for constructing structurally stable SAC frameworks with accessible catalytic interfaces [10].

To overcome the limitations of uncontrolled homogeneous nucleation in conventional MOF syntheses, diffusion‐regulated interfacial growth strategies have been explored as an effective approach to spatially confined precursor formation. In contra‐diffusion systems, two precursor solutions are separated by a porous or asymmetric membrane, and MOF formation occurs exclusively at the reaction front where the diffusing metal ions and organic linkers meet [11]. This configuration inherently suppresses bulk nucleation and enables spatially confined crystal growth because the precursor flux can be regulated through the membrane microstructure. Previous studies have shown that such diffusion‐controlled synthesis follows a self‐limiting mechanism, wherein the newly formed MOF layer gradually restricts further precursor transport and stabilizes the reaction front [12]. Such diffusion‐regulated growth provides a useful platform for constructing uniformly distributed catalytic architectures in electrochemical systems [13, 14]. Importantly, when implemented within a three‐dimensional fibrous scaffold, diffusion‐regulated precursor evolution can directly determine the spatial distribution of catalytic centers after pyrolysis, thereby enabling uniformly accessible single‐atom catalytic architectures.

**FIGURE 1:**
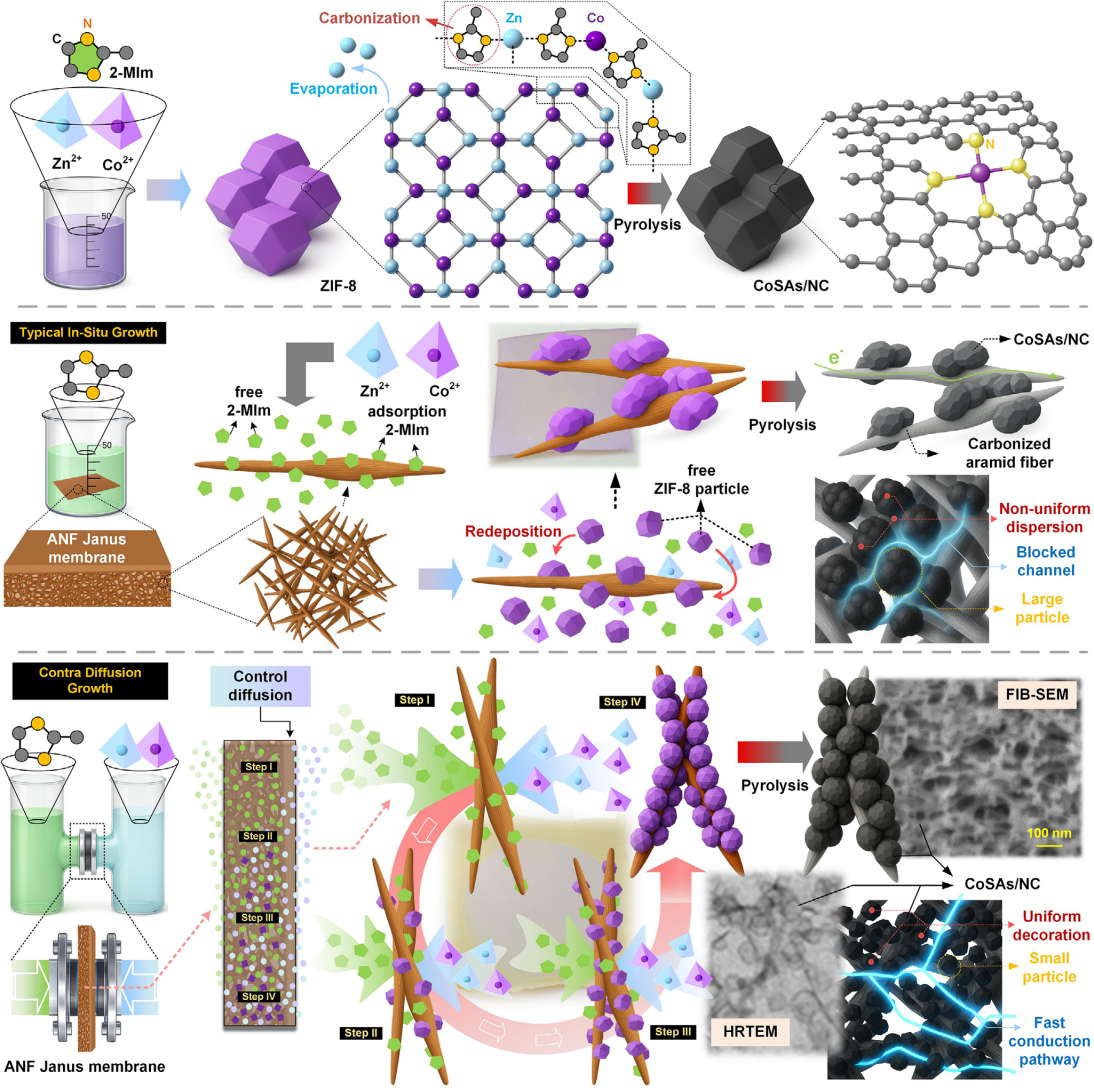
Schematic illustration of diffusion‐regulated growth of Zn‐Co ZIF precursors and the formation of atomically dispersed Co–N_4_ sites on an asymmetric ANF membrane. (i) Controlled coordination of Zn^2^^+^/Co^2^^+^ with 2‐MIm followed by pyrolysis generates atomically dispersed Co sites coordinated with nitrogen in a carbonized ANF framework (CoSAs/NC), assisted by Zn volatilization during thermal treatment. (ii) Conventional in situ growth leads to homogeneous nucleation in bulk solution, particle aggregation on ANF fibers, pore blockage, and spatially nonuniform Co–N_x_ sites after carbonization. (iii) The asymmetric ANF membrane regulates the diffusion of metal ions and ligands through its dense surface layer, confining precursor interaction within the membrane interior and producing uniformly distributed nanoscale ZIF domains. These domains are subsequently converted into homogeneously dispersed Co–N_4_ sites within a free‐standing conductive carbon network after pyrolysis.

Here, we report an asymmetric aramid‐nanofiber (ANF) Janus membrane [15] that enables diffusion‐regulated in‐situ growth of Zn‐Co ZIF precursors within the fibrous scaffold (Figure 1) [16]. In typical in‐situ polymerization systems [17, 18, 19], 2‐methyl imidazole (2‐MIm) and metal ions freely diffuse in the bulk solution, leading to uncontrolled homogeneous nucleation, particle agglomeration, and redeposition on the ANF scaffold, which ultimately results in nonuniform ZIF coverage and blocked ion‐transport channels. By engineering a dense ANF surface layer that functions as a molecular diffusion gate, our contra‐diffusion design precisely modulates the flux of both precursors and confines their encounter exclusively within the membrane interior. This stepwise diffusion‐controlled coordination chemistry produces uniformly distributed and nanoscale Zn‐Co ZIF domains intimately anchored to the ANF network. Upon pyrolysis, volatile Zn species evaporate while the ANF framework carbonizes, leaving atomically dispersed Co centers strongly coordinated by nitrogen functionalities derived from both the ZIF and ANF backbones. The resulting free‐standing Co–N_4_ interlayer exhibits highly uniform active‐site distribution, abundant ion‐transport channels, and continuous charge‐conduction pathways, representing a distinct catalytic architecture compared with conventional in‐situ‐derived composites. This work demonstrates that diffusion‐regulated precursor growth can construct a uniformly distributed single‐atom catalytic architecture within a conductive fibrous membrane, enabling efficient utilization of Co–N_x_ active sites and spatially regulated sulfur redox reactions in Li–S batteries. As a consequence, this diffusion‐regulated catalytic architecture enables efficient sulfur redox regulation and provides a structural basis for stable high‐rate cycling even at low Co loading.

## 2 Results and Discussion

### 2.1 Diffusion‐Regulated Formation and Atomic Structure of CoSAs/NC

**FIGURE 2:**
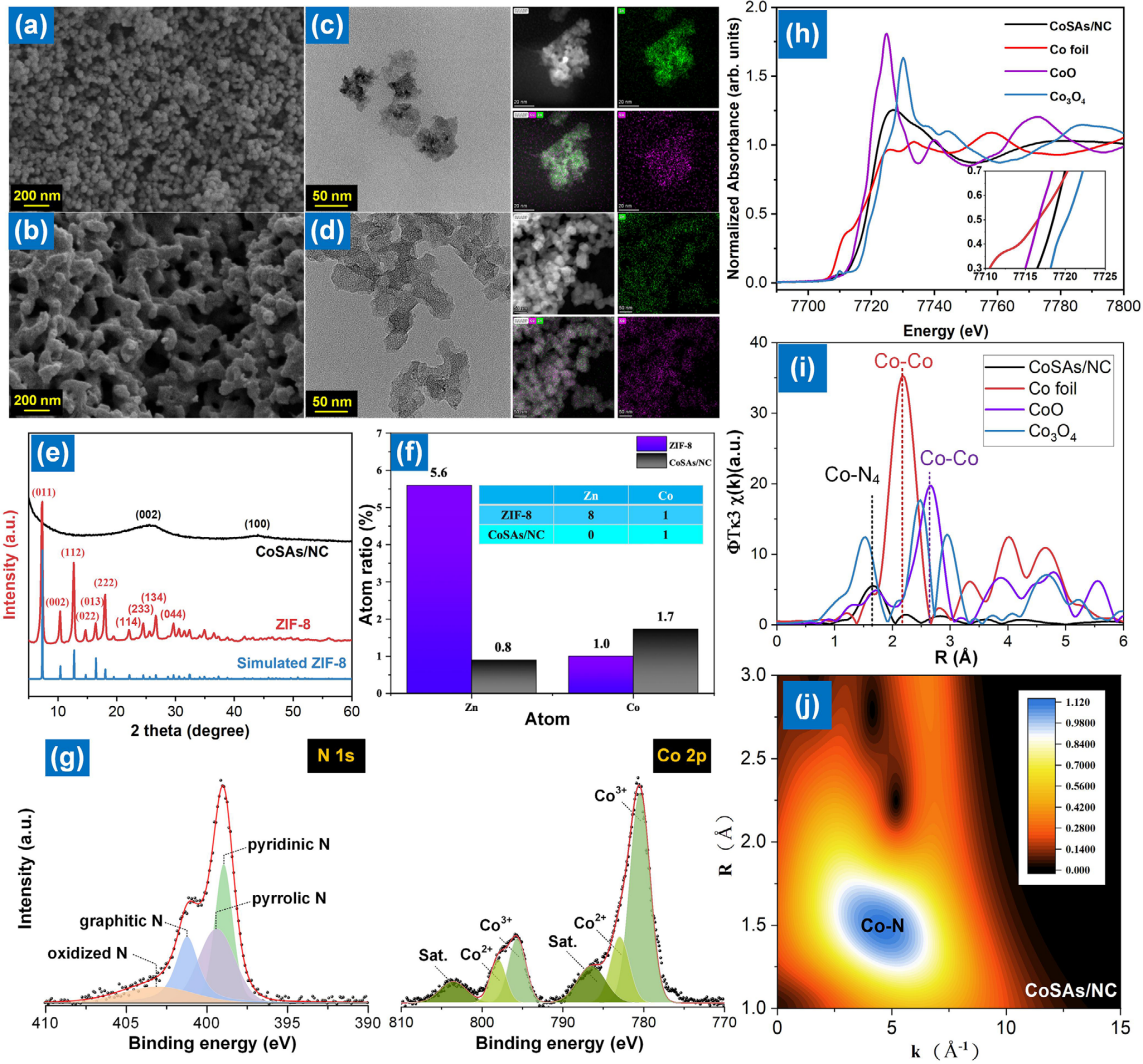
Structural characterization of the Zn‐Co ZIF precursor and the derived CoSAs/NC. (a, b) SEM images of ZIF‐8 and CoSAs/NC. (c, d) TEM/HAADF‐STEM images with elemental mapping. (e) XRD patterns of ZIF‐8 and CoSAs/NC. (f) EPMA elemental composition. (g) High‐resolution N 1s and Co 2p XPS spectra. (h) Co K‐edge XANES spectra. (i) Fourier‐transformed EXAFS. (j) Wavelet‐transform EXAFS contour map.

The pristine Zn‐Co ZIF precursor exhibits a uniform polyhedral morphology with well‐defined facets, characteristic of ZIF‐8‐type frameworks (Figure 2a,c) [20]. After pyrolysis, the crystalline ZIF structure collapses into a porous nitrogen‐doped carbon matrix composed of interconnected nanoparticles, while largely preserving the nanoscale morphology (Figure 2b,d) [21]. Notably, no large aggregates or metal‐rich domains are observed, indicating that the bimetallic ZIF precursor effectively disperses metal species prior to thermal transformation. Elemental mapping reveals a clear evolution of metal distribution during carbonization. Both Zn and Co are homogeneously distributed in the ZIF precursor, whereas in the carbonized CoSAs/NC sample, the Zn signal nearly disappears, consistent with Zn volatilization at elevated temperature, while Co remains uniformly dispersed throughout the carbon framework [22]. The absence of Co‐rich clusters in high‐resolution TEM and elemental maps suggests that Co atoms are stabilized within the nitrogen‐doped carbon matrix rather than aggregating into nanoparticles.

The structural evolution from crystalline ZIF to carbonized CoSAs/NC was further examined by x‐ray diffraction (Figures 2e and S1). The Zn‐Co ZIF precursor displays characteristic reflections corresponding to the sodalite topology of ZIF‐8, which completely vanish after pyrolysis [23]. Importantly, no diffraction peaks attributable to metallic Co or cobalt oxides are detected, supporting the absence of Co‐containing crystalline phases after carbonization. Quantitative elemental analysis by electron probe microanalysis (EPMA, Figure 2f) shows a drastic decrease of Zn content to near the detection limit after pyrolysis, while a low but appreciable Co content is retained. This result confirms the role of Zn as a sacrificial, volatile component and the immobilization of Co within the carbon framework at a loading level consistent with SACs. The Raman spectroscopy of CANF, S‐CANF, CD‐CANF, and CoSAs/NC is shown in Figure S2. All samples exhibit the characteristic D band (≈1350 cm^−1^) and G band (≈1580 cm^−1^) of carbon materials. The CANF‐derived samples display similar *I_D_*/*I_G_* ratios (≈0.9), indicating that the contra‐diffusion growth process does not significantly alter the carbon framework of the nanofiber scaffold. In contrast, CoSAs/NC shows a higher *I_D_*/*I_G_* ratio (≈1.3), suggesting a higher density of structural defects associated with the MOF‐derived carbon matrix, which can serve as anchoring sites for atomically dispersed Co–N_x_ species [24].

The chemical environments of nitrogen species and cobalt centers were investigated by x‐ray photoelectron spectroscopy (XPS) (Figure 2g). The N 1s spectrum of CoSAs/NC can be deconvoluted into pyridinic‐N, pyrrolic‐N, graphitic‐N, and oxidized‐N components (Figure S3) [25], indicating the coexistence of multiple nitrogen functionalities inherited from both the ZIF framework and the ANF backbone. Among them, pyridinic‐N and graphitic‐N are commonly regarded as effective coordination sites for stabilizing isolated metal atoms, providing a suitable electronic environment for the formation of Co–N_x_ moieties. The Co 2p spectrum displays characteristic Co^2^^+^/Co^3^^+^ features without any detectable signal corresponding to metallic Co^0^ (≈778.1 eV), excluding the presence of cobalt nanoparticles. Together with the absence of metallic Co signatures, these results suggest that cobalt is incorporated into nitrogen‐coordinated environments rather than aggregated metallic or oxide phases [26].

The local coordination structure and oxidation state of Co were further elucidated by Co K‐edge x‐ray absorption spectroscopy (Figures 2h–j and S4) [27]. The XANES edge position of CoSAs/NC lies between those of CoO and Co_3_O_4_ and is distinctly higher than that of metallic Co foil, indicating an oxidized, non‐metallic cobalt state. The enhanced white‐line intensity compared with CoO is consistent with reduced coordination symmetry and electron density associated with isolated Co–N_x_ configurations. Fourier‐transformed EXAFS spectra exhibit a dominant peak at ≈1.45 Å, attributable to Co–N scattering, while the Co–Co coordination peak at ∼2.15 Å is completely absent. Wavelet transform (WT) analysis further reveals a single intensity maximum located in the Co–N scattering region, with no features associated with Co–Co interactions. These spectroscopic results collectively confirm that cobalt exists predominantly as atomically dispersed Co–N_x_ sites. This conclusion is consistent with high‐resolution TEM and FFT analyses (Figure S5), which show no evidence of crystalline Co‐containing domains [28].

**FIGURE 3:**
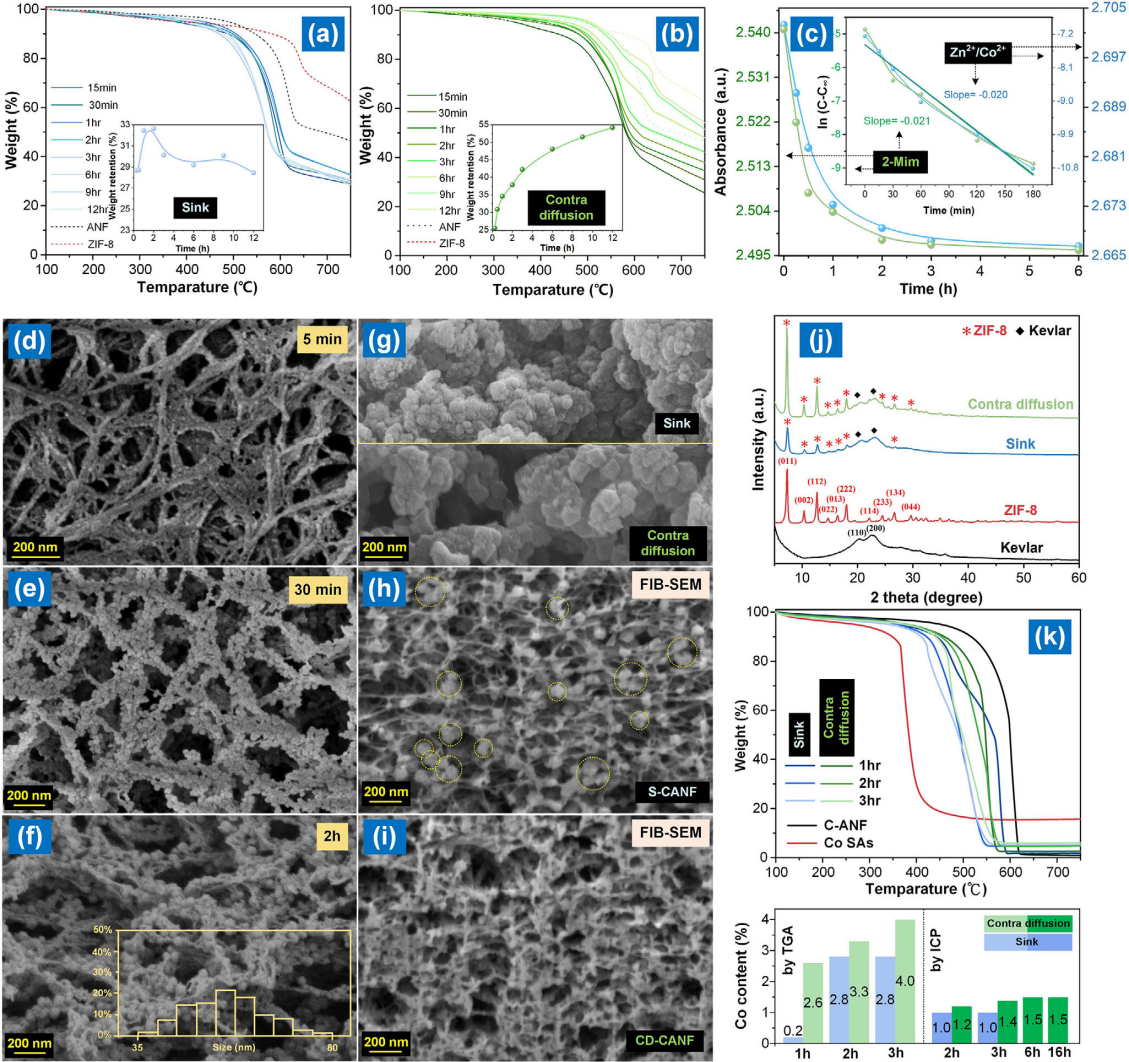
Diffusion‐regulated in situ growth of ZIF‐8 on ANF membranes *via* sink and contra‐diffusion strategies. (a, b) TGA curves of ZIF‐8 grown on ANF at different reaction times using (a) sink and (b) contra‐diffusion methods; insets show the corresponding evolution of inorganic residue, reflecting precursor uptake behavior. (c) Time‐dependent concentration profiles of 2‐MIm and Zn^2^^+^/Co^2^^+^ ions in the contra‐diffusion system with fitted diffusion kinetics. (d–f) Time‐resolved SEM images of ZIF‐8 growth on ANF under contra diffusion at 5 min, 30 min, and 2 h, respectively; insets show the corresponding particle‐size distributions. (g) SEM comparison of ZIF‐8 growth on ANF after 2 h using sink and contra‐diffusion methods. (h, i) FIB‐SEM cross‐sectional images of the carbonized S‐CANF and CD‐CANF interlayers. (j) XRD patterns of pristine ZIF‐8, Kevlar, and ZIF‐8 grown on ANF via sink and contra‐diffusion methods. (k) TGA profiles of carbonized interlayers prepared at different growth durations and corresponding Co contents determined by TGA and ICP analyses.

To elucidate the factors governing the in‐situ growth of ZIF precursors on ANF membranes, the evolution of precursor uptake was first examined by thermogravimetric analysis (Figure 3a,b). In the conventional sink‐growth mode, mass deposition is limited and irregular, whereas the contra‐diffusion configuration yields a predictable and time‐dependent increase in inorganic residue. This behavior indicates that precursor formation is directly regulated by the controlled interfacial supply of both 2‐MIm and Zn^2^^+^/Co^2^^+^ ions. Time‐resolved concentration measurements performed on both compartments of the contra‐diffusion cell (Figures 3c and S6) further quantify the transport behavior of the reactants. Fitting the concentration profiles using Fick's diffusion model gives diffusion coefficients of *D*_2 − *MIm*_ = 7.4 × 10^−9^ m^2^ s^−1^ and $D_{Zn^{2+}/Co^{2+}}$ = 7.1 × 10^−9^ m^2^ s^−1^, indicating nearly identical diffusivities. Such balanced bidirectional diffusion establishes a well‐defined reaction front within the ANF scaffold, enabling uniform nucleation and homogeneous growth of ZIF domains throughout the membrane [11].

### 2.2 Structural Evolution Toward Atomically Dispersed Co–N_x_ Sites

SEM imaging was employed to visualize the time‐dependent evolution of ZIF‐8 formation within the ANF network under contra‐diffusion conditions (Figure 3d–f). At the initial stage, only nanoscale nuclei are detected, which are already uniformly distributed along the ANF fibers. After 30 min, well‐defined ZIF‐8 particles with sizes exceeding 20 nm emerge and homogeneously decorate the fiber surfaces, forming a continuous coating. With prolonged reaction time, particle size increases gradually while the spatial distribution remains highly uniform, indicative of a reaction‐controlled nucleation‐growth process rather than uncontrolled bulk precipitation. Particle size distributions extracted from SEM images (Figures S7 and S8) exhibit a narrow and systematic evolution, further confirming kinetically moderated growth. A direct comparison after 2 h (Figure 3g) highlights the intrinsic advantage of the contra‐diffusion strategy: ZIF‐8 crystals grow conformally along the ANF scaffold without blocking pore channels, whereas the sink‐growth process leads to irregular aggregation and partial channel obstruction.

The carbonized interlayers largely inherit the morphology of their corresponding ZIF precursors, confirming that the precursor growth mode dictates the final carbon architecture after pyrolysis. As shown in Figure S9, S‐CANF prepared by immersion growth exhibits relatively aggregated carbon domains. In contrast, CD‐CANF synthesized *via* contra‐diffusion displays a more homogeneous nanoscale texture distributed throughout the fibrous scaffold. This difference indicates that the contra‐diffusion process effectively regulates precursor nucleation and spatial distribution within the ANF framework. The influence of precursor growth duration on the resulting carbon structure was further examined using CD‐CANF samples synthesized with different growth times (3–16 h) (Figure S10). The sample obtained after 3 h of growth preserves an open fibrous network with a conformal carbon coating. With increasing growth time (6–12 h), the carbon layer gradually thickens, and localized aggregates begin to appear. At 16 h, a noticeably denser carbon architecture with inter‐fiber bridging features is observed, suggesting excessive precursor accumulation followed by carbonization‐induced shrinkage. These observations indicate that prolonged growth may reduce the structural openness of the fibrous scaffold. Considering both structural characteristics and catalytic performance (Figure 5a), a growth time of 2 h was selected as the optimal condition for further study.

To further clarify the internal structural distribution of the catalytic framework, focused ion beam system (FIB‐SEM) analysis was performed for both S‐CANF and CD‐CANF interlayers (Figures 3 h,i, S11, and S12). The S‐CANF sample exhibits locally aggregated carbon domains distributed along the fibrous scaffold, indicating heterogeneous precursor growth within the network. In contrast, the CD‐CANF interlayer displays a more conformal nanoscale carbon texture covering the ANF framework, with smaller and more evenly distributed domains. This architecture preserves the inter‐fiber voids while forming continuous catalytic interfaces throughout the scaffold, providing accessible pathways for ion transport and LiPS conversion. These observations confirm that diffusion‐regulated precursor growth directly determines the spatial distribution of catalytic domains within the fibrous scaffold. The structural evolution during carbonization was further examined by XRD analysis. As shown in Figure 3j, the diffraction peaks of ZIF‐8 are clearly observed in the precursor, while after carbonization all samples (CANF, S‐CANF, and CD‐CANF) exhibit a broad (002) feature characteristic of turbostratic carbon. Notably, CD‐CANF shows a slightly sharper and more intense (002) peak (Figure S13), suggesting a more uniform carbon framework derived from the regulated precursor distribution [29]. No diffraction signals corresponding to metallic cobalt or cobalt oxides are detected, indicating the absence of crystalline cobalt aggregates.

**FIGURE 4:**
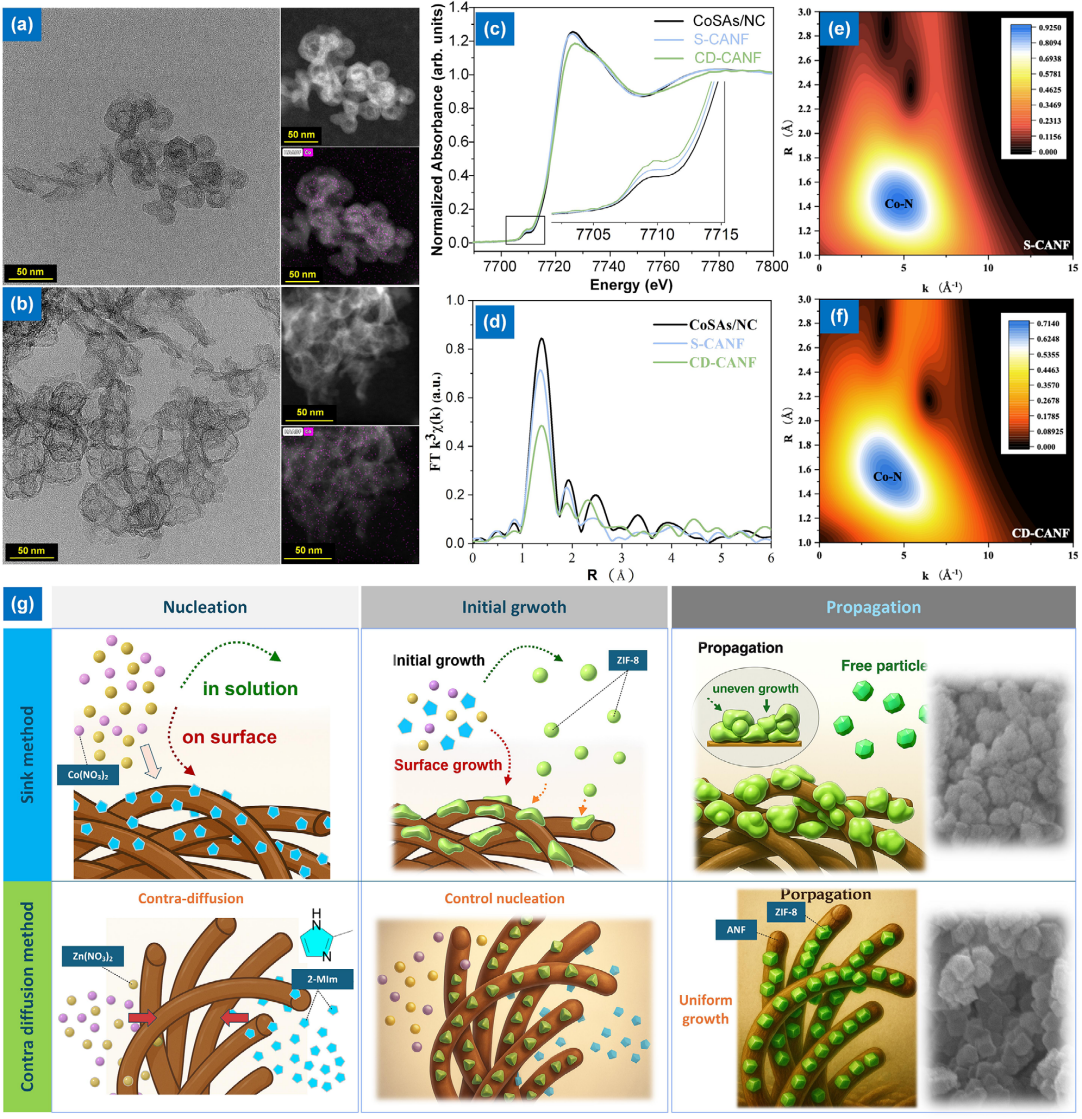
(a, b) TEM images and corresponding Co elemental mapping of carbonized interlayers prepared by the sink method (S‐CANF) and the contra‐diffusion method (CD‐CANF), respectively. (c) Co K‐edge XANES spectra of CoSAs/NC, S‐CANF, and CD‐CANF, indicating oxidized and nitrogen‐coordinated Co species. (d) Fourier‐transformed EXAFS spectra highlighting the dominant Co–N coordination and the absence of Co–Co scattering. (e, f) Wavelet transform (WT) contour plots of S‐CANF and CD‐CANF, confirming atomic dispersion of Co through the exclusive Co–N scattering feature. (g) Schematic illustration comparing the nucleation and growth mechanisms of ZIF precursors under sink and contra‐diffusion conditions, emphasizing differences in nucleation confinement, growth uniformity, and resulting Co–N_x_ coordination environments after carbonization.

The Co content in the carbonized interlayers was quantified by thermogravimetric analysis (TGA) in air and inductively coupled plasma (ICP) spectroscopy (Figure 3k). Compared with S‐CANF, the CD‐CANF consistently exhibits higher Co loading, indicating that the contra‐diffusion growth process promotes more efficient incorporation of Co species into the fibrous framework. The ICP results further reveal that the cobalt content in CD‐CANF increases slightly with growth time (1.2 wt% at 2 h, 1.4 wt% at 3 h, and ∼1.5 wt% at longer durations), suggesting progressive precursor incorporation during the diffusion‐regulated growth process. Notably, the 2 h sample already achieves a cobalt loading comparable to those obtained at extended growth times, while preserving the open fibrous architecture observed in the structural analysis. These results indicate that a moderate growth duration is sufficient to introduce an adequate amount of Co species while avoiding excessive precursor accumulation. The nanoscale distribution of Co in the carbonized interlayers was further examined by TEM and elemental mapping (Figure 4a,b). S‐CANF displays irregular carbon domains accompanied by spatially heterogeneous Co distribution, consistent with nonuniform precursor deposition during sink growth. In contrast, CD‐CANF exhibits a more homogeneous nanostructure with Co uniformly distributed throughout the framework and no observable Co‐rich clusters. This uniform distribution is consistent with the diffusion‐regulated nucleation behavior revealed in Figure 3 and supports the formation of isolated Co–N_x_ coordination environments after carbonization.

The Co K‐edge XANES spectra of S‐CANF and CD‐CANF (Figure 4c) show absorption edges positioned between those of CoO and Co_3_O_4_, indicating that cobalt in both samples exists in an oxidized, nitrogen‐coordinated state rather than as metallic Co^0^. Notably, CD‐CANF exhibits a slightly enhanced white‐line intensity relative to S‐CANF, suggesting a more localized electronic structure and a more well‐defined Co–N_x_ coordination environment [30]. This feature is consistent with the more uniform cobalt incorporation enabled by the contra‐diffusion growth process. Further insights are provided by the Fourier‐transformed EXAFS spectra (Figure 4d). Both samples display a dominant Co–N scattering peak at ≈1.4 Å, confirming the formation of atomically dispersed Co–N_x_ moieties. However, S‐CANF exhibits a broader and less resolved Co–N feature, accompanied by residual intensity extending toward the 2–3 Å region, indicative of increased structural heterogeneity and partially disordered local environments. In contrast, CD‐CANF shows a sharper Co–N peak with strongly suppressed intensity in the Co–Co scattering region, evidencing the absence of cobalt clustering and a more uniform single‐atom coordination structure [31]. These spectroscopic differences demonstrate that contra diffusion yields a more homogeneous and well‐defined Co–N_x_ configuration, whereas the sink‐grown sample contains a broader distribution of local Co environments. The superior atomic uniformity of CD‐CANF is further corroborated by wavelet transform analysis (Figure 4e,f).

Taken together, the growth kinetics, morphological evolution, carbonization behavior, and atomic‐scale coordination analyses reveal a fundamental divergence between the sink and contra‐diffusion pathways. To integrate these observations, a mechanistic model is proposed in Figure 4g. In the conventional sink process, ANF is simultaneously exposed to 2‐MIm and Zn^2^^+^/Co^2^^+^ ions, leading to rapid homogeneous nucleation in solution. This results in extensive formation of free ZIF particles, partial blockage of fiber channels, and spatially heterogeneous precursor deposition on ANF, which ultimately translates into nonuniform Co–N_x_ coordination environments after pyrolysis. By contrast, the contra‐diffusion configuration spatially separates the two reactants across the ANF membrane. 2‐MIm and Zn^2^^+^/Co^2^^+^ ions diffuse inward from opposite sides with comparable diffusion coefficients, generating a confined reaction zone within the ANF nanochannels. This spatial restriction suppresses solution‐phase nucleation and enforces heterogeneous nucleation directly on the ANF fiber surface, where coordinated amide groups and π‐rich domains provide uniform anchoring sites. Subsequent growth proceeds under diffusion‐limited conditions, yielding highly homogeneous ZIF‐8 domains without pore blockage. After carbonization, this precursor uniformity is preserved as atomically dispersed Co–N_x_ sites, as reflected by the sharp Co–N scattering and complete suppression of Co–Co contributions in EXAFS. Overall, this mechanistic analysis identifies contra‐diffusion as the decisive factor governing controlled nucleation, uniform precursor evolution, and the formation of a highly homogeneous Co–N_x_ single‐atom coordination environment in CD‐CANF.

### 2.3 Polysulfide Adsorption, Catalytic Activity, and Ion‐Electron Transport in the Engineered Interlayers

To correlate precursor growth regulation with the resulting textural properties, N_2_ adsorption–desorption measurements were conducted for CoSAs/NC, S‐CANF, and CD‐CANF (Figure S14). CD‐CANF exhibits the highest BET surface area (362 m^2^ g^−1^), significantly exceeding those of S‐CANF (316 m^2^ g^−1^) and CoSAs/NC (70 m^2^ g^−1^), together with a narrow mesopore distribution centered at ≈2–3 nm. In contrast, S‐CANF shows a broader and less well‐defined pore profile. These differences directly reflect the diffusion‐regulated ZIF growth enabled by the contra‐diffusion strategy, which suppresses free‐particle aggregation and preserves the intrinsic ANF pore network. The resulting combination of high surface area and uniform mesoporosity in CD‐CANF maximizes the accessibility of Co–N_x_ sites and provides an optimal structural basis for LiPS confinement and catalytic conversion.

**FIGURE 5:**
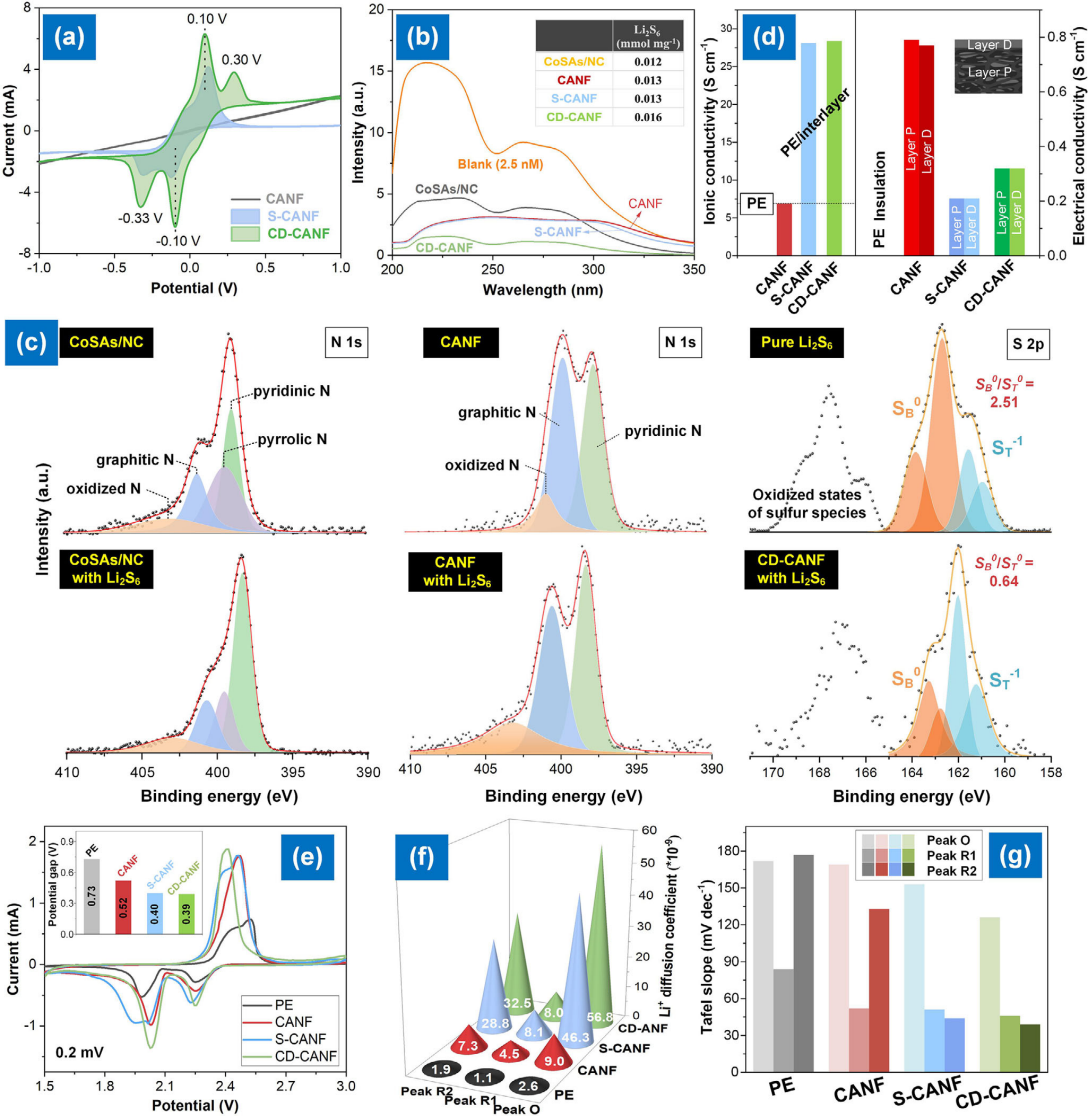
(a) CV profiles of symmetric cells employing CANF, S‐CANF, and CD‐CANF interlayers, highlighting the characteristic redox responses of LiPSs. (b) UV–vis absorption spectra of Li_2_S_6_ solutions after contact with different interlayers, reflecting their relative LiPS adsorption capability. (c) N 1s XPS spectra of pristine CoSAs/NC, CANF, and CD‐CANF, together with the corresponding spectra after Li_2_S_6_ adsorption, illustrating the interfacial interaction between nitrogen‐coordinated Co sites and LiPS species. (d) Ionic conductivity of PE separators modified with different interlayers (left), and electronic conductivity measured on the dense (Layer D) and porous (Layer P) sides of the interlayers (right). (e) CV curves of Li–S full cells assembled with PE, CANF, S‐CANF, and CD‐CANF interlayers at various scan rates. (f) Apparent $D_{Li^{+}}$ derived from Randles–Sevcik analysis of the three characteristic redox peaks (R2, R1, and O). (g) Tafel slopes extracted from the anodic (O) and cathodic (R1 and R2) processes, reflecting the reaction kinetics associated with different interlayers.

The intrinsic catalytic activity toward LiPS conversion was next evaluated using symmetric‐cell configurations, allowing direct comparison of redox kinetics independent of sulfur loading or electrode architecture. As shown in Figure S15a, CoSAs/NC exhibits markedly stronger and more defined redox features than pristine CNT, confirming the high intrinsic catalytic activity of atomically dispersed Co–N_x_ sites. To further elucidate the role of precursor evolution, interlayers prepared at different growth durations (1, 2, and 3 h) under sink and contra‐diffusion conditions were systematically examined by cyclic voltammetry (CV) (Figure S15c,d). With increasing growth time, progressively sharper and more symmetric LiPS redox peaks are observed, consistent with the formation of more uniform ZIF‐derived domains and enhanced exposure of Co–N_x_ active sites. At all reaction durations, CD‐CANF consistently outperforms S‐CANF, highlighting the decisive advantage of diffusion‐controlled nucleation. Based on these time‐dependent trends, the 2 h samples, exhibiting the optimal balance between catalytic activity, structural uniformity, and pore accessibility, were selected for subsequent electrochemical analyses. As shown in Figure 5a, CD‐CANF delivers substantially sharper and more intense redox features than CANF and S‐CANF, indicating faster LiPS conversion kinetics and a more efficient catalytic interface, whereas CANF displays broad and poorly defined peaks characteristic of limited catalytic contribution [32, 33].

The LiPS adsorption capability of the interlayers was further assessed by UV–vis spectroscopy (Figure 5b). Among the three samples, CD‐CANF induces the most pronounced decrease in Li_2_S_6_ absorbance, indicating the strongest affinity toward soluble LiPS species. S‐CANF shows moderate adsorption, whereas CANF exhibits only a marginal effect. This trend can be rationalized by the larger accessible surface area, well‐developed mesoporosity, and uniformly exposed Co–N_x_ anchoring sites in CD‐CANF, which collectively favor efficient LiPS capture. Together with the symmetric‐cell results, these observations establish that CD‐CANF simultaneously enhances LiPS immobilization and catalytic conversion, providing a clear advantage over CANF and S‐CANF. To elucidate the underlying interfacial chemistry, XPS analyses were performed on CANF, S‐CANF, and CD‐CANF after Li_2_S_6_ adsorption (Figures 5c and S16). In the N 1s spectra, both S‐CANF and CD‐CANF exhibit discernible positive shifts in the pyridinic‐N and Co–N_x_ components, indicative of electron transfer from LiPS species to nitrogen‐coordinated cobalt centers. Notably, the shift is most pronounced for CD‐CANF, consistent with its higher density of electronically accessible Co–N_x_ sites and the superior adsorption behavior observed in Figure 5b. In contrast, CANF shows negligible spectral changes, confirming its weak chemical interaction with LiPSs.

Further insight is provided by the S 2p spectra, which reveal progressive stabilization of reduced sulfur species. Compared with CANF (*S_B_^0^*/*S_T_^−1^* = 0.55), higher fractions of terminal sulfur species are observed for S‐CANF (0.61) and CD‐CANF (0.64), indicating increasingly strong interactions with LiPSs. In particular, CD‐CANF displays enhanced LiPS‐related signals together with emerging features attributable to thiosulfate/polythionate intermediates, characteristic of chemically activated sulfur species. These results suggest that CD‐CANF not only captures LiPSs more effectively but also shifts their surface speciation toward more reactive, catalytically relevant states. Consistent with this interpretation, the Co 2p spectra (Figure S17) show a distinct positive shift of the Co 2p_3/2_ peak for CD‐CANF after Li_2_S_6_ adsorption, accompanied by suppression of satellite features, reflecting strong Co–S coordination and direct electronic coupling between Co–N_x_ sites and LiPS intermediates [34]. These changes are less evident for S‐CANF and nearly absent for CANF, mirroring the catalytic activity hierarchy established in Figure 5a. Collectively, the N 1s, S 2p, and Co 2p analyses converge to demonstrate that CD‐CANF possesses the strongest chemical affinity and activation capability toward LiPSs. This well‐defined interfacial interaction, enabled by uniformly dispersed and fully accessible Co–N_x_ sites, provides the mechanistic foundation for the accelerated LiPS conversion kinetics and superior electrochemical performance discussed in the following sections.

Following the catalytic and adsorption analyses, the ion and electron transport properties of the interlayers were systematically evaluated, as both are critical for sustaining fast sulfur redox under high‐rate operation. As shown in Figure 5d (left), the introduction of ZIF‐derived Co–N_x_ coordination environments leads to a clear enhancement in ionic conductivity compared with pristine CANF. While S‐CANF shows a moderate increase, CD‐CANF exhibits the highest ionic conductivity, which can be attributed to its continuous mesoporous architecture and uniformly distributed polar coordination sites that facilitate Li^+^ migration across the interlayer. In contrast, the electronic conductivity (Figure 5d, right) follows a different trend. Incorporation of ZIF‐derived domains inevitably perturbs the intrinsic electron‐conduction pathways of the ANF framework, resulting in lower conductivity relative to pristine CANF. Notably, this reduction is significantly less pronounced in CD‐CANF than in S‐CANF. The diffusion‐regulated growth in CD‐CANF minimizes local aggregation and pore blockage, thereby preserving fiber‐fiber contact within the ANF network and maintaining an effective electronic percolation pathway. In comparison, the less controlled precursor growth in S‐CANF leads to spatially heterogeneous particle deposition, which more severely disrupts electronic transport. The combination of enhanced Li^+^ mobility and preserved electronic connectivity in CD‐CANF therefore provides a favorable transport environment that synergistically complements its catalytic Co–N_x_ sites.

To further quantify the kinetic advantages imparted by the contra‐diffusion strategy, CV measurements were performed using Li–S full cells incorporating CANF, S‐CANF, and CD‐CANF interlayers (Figure 5e), with additional symmetric‐cell comparisons shown in Figure S18. The separation between the cathodic and anodic peaks (*ΔE*) decreases progressively from PE (0.54 V) to CANF (0.46 V), S‐CANF (0.45 V), and CD‐CANF (0.38 V), indicating increasingly accelerated LiPS redox kinetics. The substantially reduced *ΔE* for CD‐CANF reflects lower polarization and faster charge‐transfer processes during both sulfur reduction and Li_2_S oxidation [35]. By contrast, CANF exhibits broad and sluggish redox features due to the absence of catalytic sites, while S‐CANF provides only partial improvement owing to its less uniform structure and partially hindered ion transport. Taken together, these results demonstrate that diffusion‐regulated ZIF growth uniquely integrates catalytic activity with balanced ion‐electron transport, enabling a uniformly active interlayer interface and markedly accelerated LiPS conversion kinetics, in full agreement with the trends observed in Figure 5a–d.

To further elucidate the kinetic advantages imparted by the contra‐diffusion strategy, the LiPS conversion behavior of the interlayers was quantitatively analyzed through redox peak separation, diffusion kinetics, and Tafel analysis. As summarized in Figure 5e–g, and Figures S19 and S20, CD‐CANF consistently exhibits the smallest redox peak separation, the highest slopes in the Randles‐Ševčík plots (*I_p_* vs. *ν^1^/^2^*), and the lowest Tafel slopes among all samples. These features collectively indicate reduced polarization, accelerated Li^+^ diffusion, and lower activation barriers for LiPS redox reactions. In contrast, pristine PE and CANF display broad redox peaks, large *ΔE* values, sluggish diffusion behavior, and steep Tafel slopes, reflecting kinetically hindered charge transfer and the absence of effective catalytic sites. S‐CANF provides partial improvement but remains inferior to CD‐CANF, consistent with its less uniform precursor‐derived structure and a lower density of accessible Co–N_x_ active centers. The superior kinetics of CD‐CANF therefore arise from the synergistic integration of a homogeneous ZIF‐derived nanostructure, uniformly dispersed Co–N_x_ catalytic sites, and a well‐preserved ANF conductive framework.

### 2.4 Electrochemical Kinetics and Rate‐Dependent Sulfur Conversion

**FIGURE 6:**
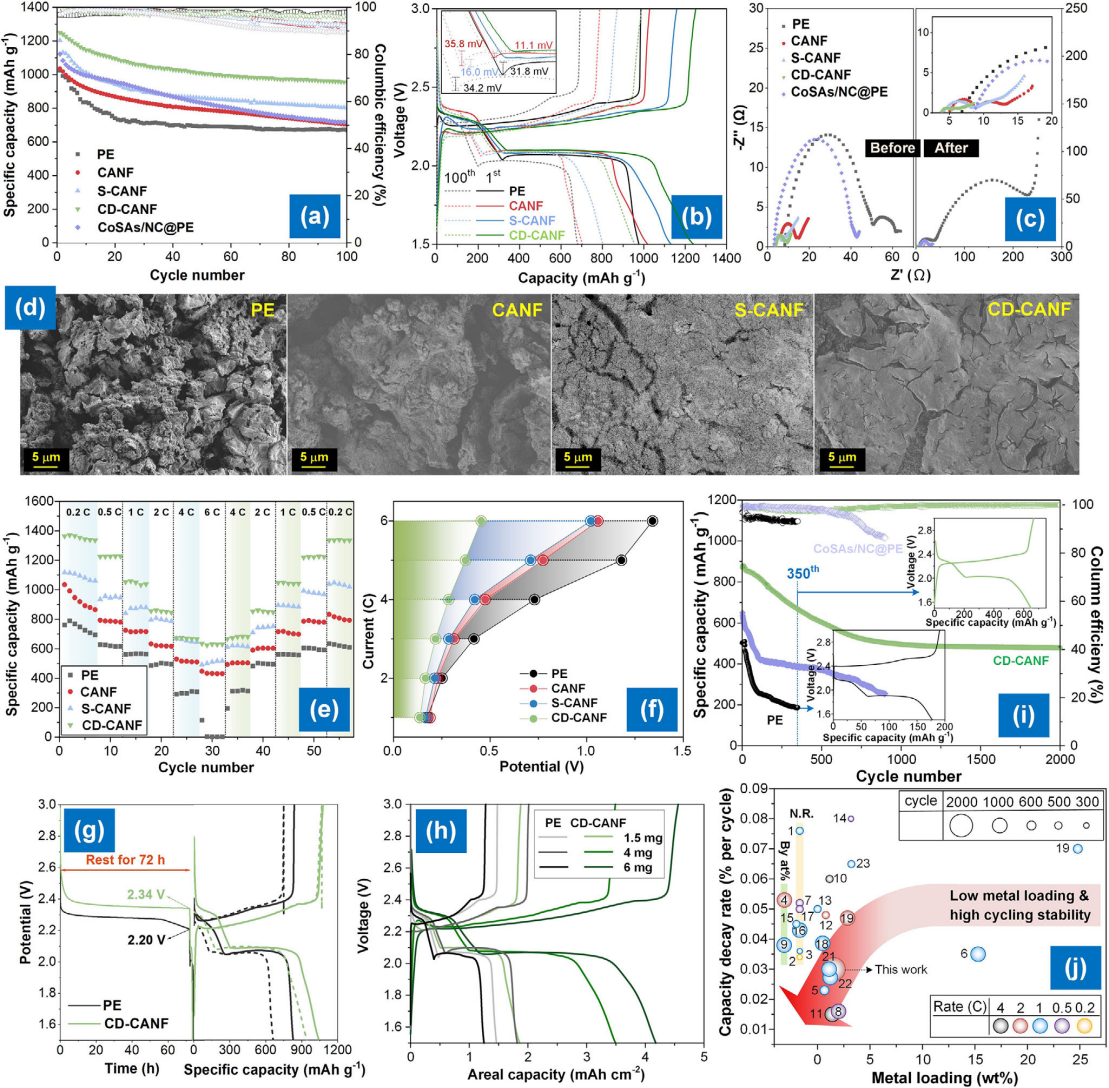
(a) Cycling performance of Li–S cells using PE, CANF, S‐CANF, CD‐CANF, and CoSAs/NC@PE at 0.5 C. (b) Charge–discharge voltage profiles of the corresponding cells, with the potential gaps annotated for comparison of polarization. (c) Nyquist plots before and after cycling, showing the evolution of charge‐transfer resistance. (d) SEM images of the Li anode after cycling with different interlayers. (e) Rate performance measured from 0.2 C to 6 C and returning to 0.2 C. (f) Potential gap at 50% discharge capacity. (g) Self‐discharge behavior of PE and CD‐CANF cells after resting for 72 h. (h) Voltage profiles of PE and CD‐CANF cells at various sulfur areal loadings (1.5–6 mg cm^−^^2^). (i) Long‐term cycling stability of the cell using PE, CD‐CANF, and CoSAs/NC@PE at 2 C. (j) Comparison of representative catalytic systems for Li–S batteries reported in recent literature, correlating catalyst loading with capacity decay rate under long‐term cycling. Systems based on cathode hosts, separator modifications, and interlayer catalysts are summarized from Table S2. The CD‐CANF interlayer developed in this work occupies the lower‐left region of the performance landscape, indicating low catalyst loading together with excellent cycling durability under high‐rate conditions. [1. CoSA‐NB (CoN_3_B); 2. Co SAs on CN nanosheets (CC@CN‐SACo); 3. Fe‐N_4_ (FeANAC/OC); 4. V‐N‐C (V SAs + VN NPs); 5. Co‐SAs@NC; 6. CoSA‐N‐C (Co‐N_4_ SAC); 7. Co‐O axially coordinated SAs; 8. Co‐N_x_ SAs; 9. Co‐N/P‐S asymmetric SAs; 10. FeSA‐CN; 11. Nb‐SAs@NC; 12. FeSA‐PCNF; 13. Ni‐N_5_/HNPC; 14. SACo/NDC; 15. CoSA (ALD), 16. CoSAC‐NC; 17. axial Co‐O SAs (MOF‐Co‐O); 18. Fe‐N_5_/GCNC; 19. Co‐O_2_N_2_ SAC (Co/NOC); 20. Co SA array/MOF‐NS; 21. Sm‐N_3_C_3_ SAC; 22. CoN_4_‐CoNCNF; 23. CNT@CoSA (ALD).]

To further elucidate the role of catalytic architecture, a control system was constructed by coating Co‐SAs/NC catalysts onto a commercial PE separator (Co‐SAs/NC@PE), with a thickness of ≈6 µm and a loading of ≈1.1 mg cm^−2^, in comparison to the CD‐CANF interlayer (≈35 µm, ≈1.8 mg cm^−2^). For comparison, the Co‐SAs/NC@PE system is included to distinguish the effect of catalytic architecture from intrinsic catalytic activity. The kinetic advantages of CD‐CANF translate directly into improved cycling stability and voltage characteristics in full Li–S cells. As shown in Figure 6a, the Co‐SAs/NC@PE cells deliver improved initial capacity relative to the bare PE separator, confirming the intrinsic catalytic activity of Co–N_x_ sites; however, a noticeably faster capacity decay is observed upon cycling, indicating limited structural stability of the surface‐coated configuration. The charge–discharge profiles (Figures 6b, and S21–S22), together with the potential‐gap analysis in Figure S23, further reveal increased polarization for Co‐SAs/NC@PE compared with CD‐CANF, suggesting sluggish reaction kinetics and incomplete conversion during cycling. Cells employing PE or CANF interlayers suffer from rapid capacity decay and pronounced voltage polarization, consistent with weak LiPS confinement and sluggish conversion kinetics. S‐CANF exhibits moderate improvement but still displays progressive discharge‐plateau distortion upon cycling. In sharp contrast, CD‐CANF maintains the highest reversible capacity and the most stable voltage profiles over prolonged cycling, accompanied by markedly reduced polarization in both the upper (S_8_ → Li_2_S_4_) and lower (Li_2_S_4_ → Li_2_S) discharge plateaus. Notably, the inset of Figure 6b reveals that CD‐CANF exhibits the smallest Li_2_S nucleation overpotential difference (*ΔE_n_*), indicating a facilitated nucleation‐growth process. This behavior is fully consistent with the symmetric‐cell kinetics (Figure 5a), diffusion analysis (Figure 5e), and Tafel trends (Figure 5g), confirming that CD‐CANF provides the most efficient LiPS conversion pathways. Supplementary discharge profiles at different cycle numbers further demonstrate that CD‐CANF preserves plateau definition and discharge capacity with minimal voltage drift, whereas PE‐, CANF‐, and S‐CANF‐based cells exhibit increasingly broadened plateaus and aggravated polarization. These results demonstrate that the uniformly distributed Co–N_x_ catalytic sites and intact ANF conductive network in CD‐CANF synergistically suppress LiPS shuttling, accelerate solid–liquid–solid sulfur redox, and stabilize Li_2_S deposition, thereby delivering superior cycling stability and electrochemical reversibility [36]. These trends are further reflected in the long‐term cycling behavior (Figure 6i), where the Co‐SAs/NC@PE system exhibits rapid capacity fading despite the presence of catalytic sites. Collectively, these results show that the superior performance of CD‐CANF originates not merely from the presence of Co–N_x_ catalytic sites, but from the spatially regulated catalytic architecture enabled by the contra‐diffusion design, which ensures uniform sulfur redox reactions, suppressed LiPS shuttling, and stabilized Li_2_S deposition [36]. Cross‐sectional FIB analysis (Figure S24) reveals that the Co‐SAs/NC layer is primarily confined to a thin surface coating (≈6 µm) on the PE separator, lacking a continuous three‐dimensional conductive framework. Such a configuration leads to nonuniform catalytic distribution and inefficient ion/electron transport pathways, which likely contributes to the rapid performance degradation observed during cycling.

Electrochemical impedance spectroscopy (EIS) (Figure 6c) reveals that CD‐CANF significantly reduces both the charge‐transfer resistance (*R_ct_*) and the middle‐frequency resistance (*R_sf_*), the latter being associated with LiPS diffusion and parasitic reactions at the Li‐metal interface. While PE and CANF exhibit large *R_sf_* values that further increase after cycling, indicative of severe LiPS crossover and nonuniform Li^+^ flux, CD‐CANF maintains the lowest and most stable *R_sf_* among all samples. In contrast, the Co‐SAs/NC@PE control exhibits a more pronounced increase in interfacial resistance after cycling, suggesting that the surface‐coated configuration suffers from progressive interfacial degradation and inefficient regulation of LiPS transport. This behavior indicates effective suppression of LiPS migration and more homogeneous Li^+^ transport toward the anode. Consistently, post‐mortem SEM analysis of the Li metal (Figures 6d and S25) shows that PE and CANF induce rough, mossy Li deposits, whereas CD‐CANF enables a smooth and compact Li surface, evidencing uniform Li deposition and a stabilized anode interface. The concurrent reduction of *R_ct_* and *R_sf_* demonstrates that CD‐CANF not only accelerates interfacial charge transfer but also mitigates LiPS‐induced interfacial degradation. These results are consistent with the enhanced cycling stability observed in Figure 6i and can be attributed to the spatially regulated catalytic architecture, which promotes uniform ion flux and suppresses interfacial instability during cycling.

The rate capability of the interlayers was further evaluated by galvanostatic charge‐discharge measurements at increasing current densities (Figures 6e and S26). With an increasing rate, PE and CANF cells exhibit pronounced polarization, manifested by smeared discharge plateaus and elevated charge overpotentials, while S‐CANF shows only partial mitigation. In contrast, CD‐CANF preserves well‐defined discharge plateaus even at high rates (4–6 C), indicating highly favorable reaction kinetics. Quantitative analysis of the capacity contributions from the high‐ and low‐voltage plateaus (*Q_H_* and *Q_L_*, Figure S27) reveals that CD‐CANF retains a substantially larger fraction of *Q_L_* at elevated rates. Since *Q_L_* corresponds to the kinetically demanding conversion of soluble LiPSs to solid Li_2_S_2_/Li_2_S, its superior retention directly reflects accelerated solid‐phase conversion enabled by the uniformly dispersed Co–N_x_ sites. The kinetic advantage of CD‐CANF is further quantified by the potential gap between charge and discharge at 50% depth of discharge (Figure 6f). At all current densities, the polarization follows the order CD‐CANF < S‐CANF < CANF < PE, confirming that the contra‐diffusion‐derived interlayer imposes the lowest kinetic penalty under dynamic operation. Together, the impedance response, Li‐metal morphology, rate‐dependent voltage profiles, and *Q_L_* retention consistently demonstrate that CD‐CANF enables rapid and reversible sulfur redox kinetics, underpinning its superior rate performance and long‐term cycling stability. These results collectively indicate that the diffusion‐regulated architecture effectively promotes fast solid‐liquid‐solid conversion kinetics, which is essential for sustaining high‐rate operation in Li–S batteries.

Building on the rate‐dependent kinetic, we further evaluated the performance of the interlayers under conditions approaching practical and extreme operation. Figure 6g compares the Li_2_S nucleation behavior using a potentiostatic discharge protocol. The PE cell exhibits a large nucleation overpotential, indicating a high energy barrier for solid‐phase Li_2_S formation. CANF provides only marginal improvement, consistent with its lack of catalytic functionality. In contrast, CD‐CANF markedly reduces the nucleation overpotential to ∼2.34 V, evidencing a substantially facilitated Li_2_S nucleation process. This reduced nucleation barrier is consistent with enhanced *Q_L_* retention at high rates (Figure S27), confirming that the diffusion‐regulated architecture enables spatially distributed nucleaton centers of the uniformly dispersed Co–N_x_ sites, enabling energetically favorable and spatially uniform Li_2_S deposition. The robustness of this kinetic advantage was further examined under high‐sulfur loadings (Figure 6h). While cells employing PE and CANF suffer from pronounced polarization growth and rapid capacity decay as the areal sulfur loading increases from 1.5 to 6.0 mg cm^−^^2^, CD‐CANF maintains stable discharge plateaus and delivers the highest areal capacities across all loadings. Even at 4–6 mg cm^−^^2^, the voltage profiles remain well‐defined, confirming that the diffusion‐regulated interlayer architecture enables spatial regulation of catalytic sites, thereby sustaining fast sulfur redox kinetics and efficient sulfur utilization under practical mass loadings.

To probe the ultimate durability and rate tolerance of the interlayer design, long‐term cycling was performed at a high current density of 2 C (Figure 6i), directly comparing the pristine PE and CD‐CANF. Under these demanding conditions, the PE cell exhibits rapid capacity decay and unstable Coulombic efficiency, indicative of accelerated parasitic reactions and irreversible sulfur loss. In sharp contrast, the CD‐CANF cell demonstrates exceptionally stable cycling over extended operation, retaining a high reversible capacity with a low decay rate and Coulombic efficiency approaching 100%. Notably, well‐defined discharge‐charge plateaus are preserved even after prolonged cycling, confirming sustained sulfur redox reversibility at high rates. To place this high‐rate durability in a broader context, representative interlayer‐ and separator‐modification strategies reported in recent literature are summarized in Table S1 and visualized in the two‐dimensional performance landscape in Figure 6j and Table S2, correlating catalyst loading with capacity decay rate across different catalytic configurations. Several representative SAC‐based interlayer systems prepared by advanced fabrication strategies, including template‐assisted precursor confinement and atomic layer deposition (ALD), are also included for comparison in Table S2. Notably, most reported low‐decay systems rely on catalyst incorporation directly into the sulfur cathode or host matrix, where catalytic sites are maximally utilized through intimate contact with active sulfur species. In contrast, interlayers operate in a spatially separated manner and are not the primary reaction phase, making it intrinsically more challenging to achieve comparable catalytic efficiency and long‐term stability. Within this comparison, CD‐CANF occupies the lower‐left region of the landscape, combining low Co loading with an exceptionally low decay rate under prolonged high‐rate cycling. Notably, compared with SAC interlayers fabricated by more sophisticated deposition approaches such as atomic layer deposition, the diffusion‐regulated CD‐CANF architecture achieves comparable or improved cycling durability while maintaining a lower catalyst loading and a fully freestanding fibrous scaffold. This advantageous positioning indicates that the durability of CD‐CANF does not arise from excessive catalyst content or direct cathode modification, but rather from the spatially regulated dispersion and efficient utilization of Co–N_x_ single‐atom sites enabled by the diffusion‐regulated architecture. Overall, these results highlight the capability of the interlayer to deliver such pronounced kinetic regulation and cycling durability, underscoring the effectiveness of interface‐centered catalyst design, with spatial regulation being critical for maximizing single‐atom utilization and achieving durable high‐rate performance in Li–S batteries.

### 2.5 Mechanistic Insights Into Bidirectional Sulfur Redox Regulation

**FIGURE 7:**
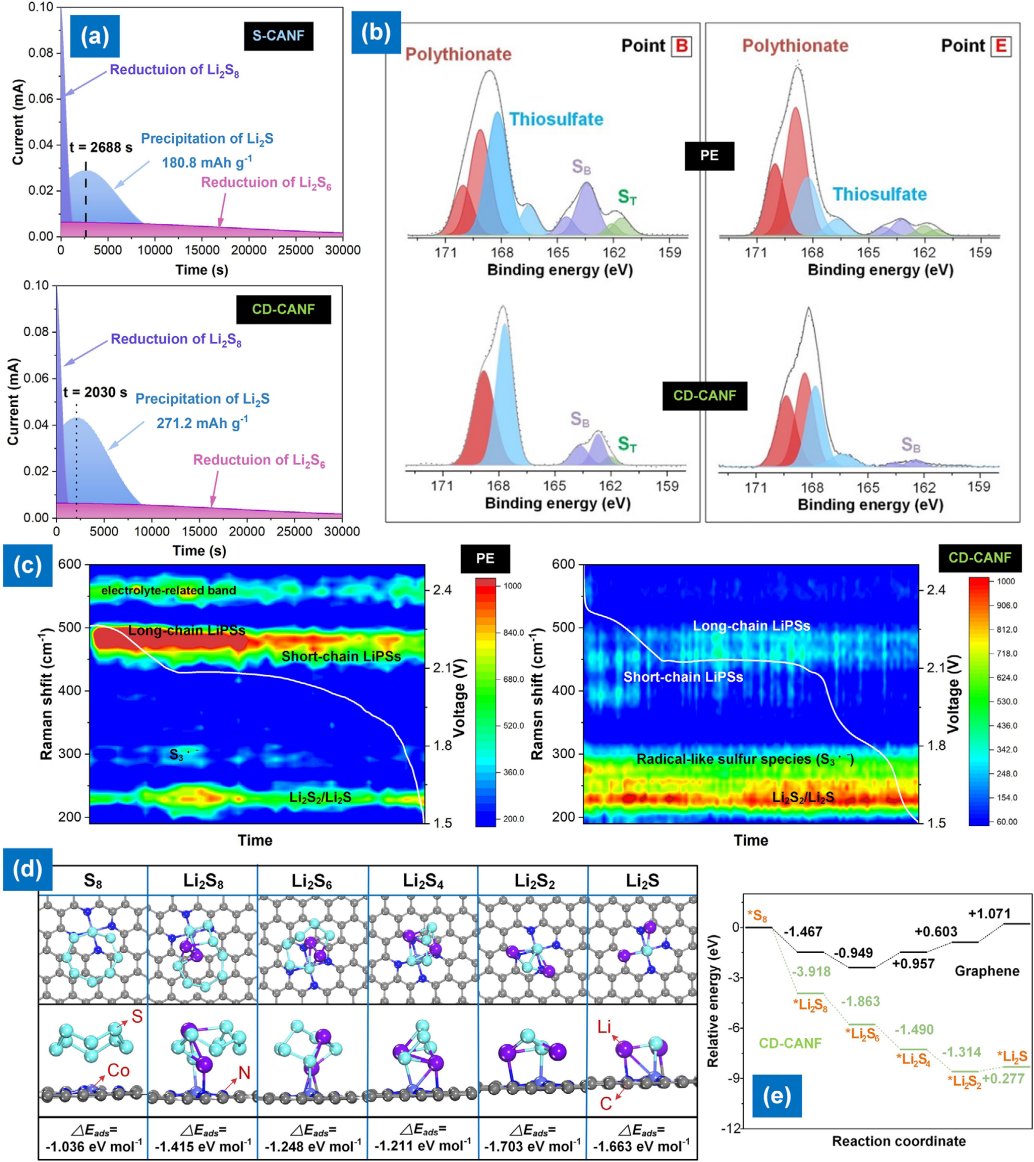
Mechanistic investigation of sulfur redox regulation by the CD‐CANF interlayer. (a) Potentiostatic Li_2_S precipitation profiles of CANF, S‐CANF, and CD‐CANF, highlighting the induction time and Li_2_S precipitation capacity. (b) Ex situ S 2p XPS spectra collected at representative discharge/charge states (points B and E) for PE and CD‐CANF cells, revealing distinct distributions of sulfur intermediates. (c) In situ Raman spectrum during the discharging process of PE and CD‐CANF cells at an excitation wavelength of 532 nm. (d) DFT‐calculated adsorption configurations of sulfur species (S_8_ to Li_2_S) on Co–N_x_ sites. (e) Corresponding reaction energy diagram for sulfur conversion on CD‐CANF, illustrating reduced energy barriers and stabilized intermediates.

To directly compare the Li_2_S precipitation and dissolution behaviors of different interlayers, potentiostatic measurements were first performed for CNT and CoSAs/NC (Figure S28). As shown in the upper panels of Figure S28, CoSAs/NC displays a substantially shortened induction time and a much higher Li_2_S precipitation capacity than pristine CNT, indicating significantly accelerated nucleation kinetics and a more efficient conversion from soluble polysulfide species to solid Li_2_S. In contrast, the CNT interlayer exhibits sluggish current evolution and limited precipitation capacity, reflecting kinetically hindered Li_2_S formation in the absence of catalytic sites. During the reverse process, CoSAs/NC also delivers a larger Li_2_S dissolution capacity accompanied by a faster current decay, suggesting facilitated solid‐to‐liquid conversion and improved reaction reversibility, whereas CNT shows markedly slower dissolution kinetics. These distinct behaviors between CNT and CoSAs/NC indicate that atomically dispersed Co–N_x_ sites provide a more uniformly accessible catalytic interface for both forward and reverse sulfur conversion processes. Such bidirectional catalytic regulation is schematically illustrated in Figures 7a and further supported by the Li_2_S precipitation behavior shown in Figure S29. As depicted, isolated Co–N_x_ single‐atom centers serve as effective adsorption and conversion sites for LiPS intermediates, lowering the kinetic barrier for Li_2_S nucleation during discharge while also enabling its efficient decomposition during charging [37]. Consequently, the uniformly distributed Co SACs promotes more continuous sulfur redox reactions, providing a mechanistic basis for the improved cycling stability observed in SAC‐modified interlayers.

To further verify the evolution of sulfur species during cycling, ex situ XPS analysis was conducted on the cathodes harvested at representative discharge states, corresponding to points B and E marked in the galvanostatic profiles (Figures 7b and S30). As illustrated in the potential‐capacity curves, point B is located at the end of the first discharge plateau, where soluble long‐chain LiPSs are actively converted, whereas point E corresponds to the deep discharge region dominated by Li_2_S formation. As shown in Figure 7b, the S 2p spectra collected at point B reveal the coexistence of thiosulfate and polythionate species, indicating the involvement of surface‐mediated LiPS conversion pathways during the intermediate discharge stage. Compared with the PE cathode, the CD‐CANF cathode exhibits a higher proportion of these oxidized sulfur intermediates, suggesting that diffusion‐regulated catalytic architecture promotes more effective activation and stabilization of LiPS species on the uniformly distributed Co–N_x_ sites. At point E, the S 2p spectra are dominated by signals corresponding to *S_T_* and *S_B_*, characteristic of solid Li_2_S formation. Notably, the CD‐CANF cathode shows a more uniform sulfur speciation distribution with suppressed accumulation of inactive sulfur species, indicating that the diffusion‐regulated catalytic architecture promotes a spatially regulated Li_2_S deposition process. In contrast, the PE cathode displays a relatively broader and less defined sulfide signal, implying heterogeneous deposition and incomplete sulfur utilization. These results provide direct spectroscopic evidence that the Co SA‐modified interlayer influences sulfur speciation at different discharge stages. Importantly, the diffusion‐regulated growth of the CD‐CANF framework ensures that these catalytic sites are homogeneously distributed throughout the interlayer, enabling spatially uniform sulfur conversion and suppressing localized Li_2_S accumulation. Combined with the reaction pathway illustrated in Figure 7a, the XPS analysis suggests that the diffusion‐regulated Co–N_x_ catalytic sites facilitate controlled LiPS conversion at the intermediate stage (point B) and promotes uniform Li_2_S formation at deep discharge (point E).

To directly probe how the interlayer architecture influences sulfur conversion processes, in situ Raman spectroscopy was employed to track the evolution of LiPS species during discharge in cells using PE and CD‐CANF (Figure 7c), with the Raman contour maps correlated to the real‐time voltage profiles. For the PE cell, intense Raman bands in the 480–520 cm^−1^ region, characteristic of long‐chain LiPSs, persist throughout a large portion of discharge, indicating sluggish conversion and continuous accumulation of soluble species. Signals associated with short‐chain LiPSs (≈420–460 cm^−1^) remain weak and diffuse, while a broad Li_2_S_2_/Li_2_S‐related band (≈200–230 cm^−1^) emerges gradually and heterogeneously, reflecting kinetically hindered and spatially nonuniform solid‐phase precipitation. Occasional features assignable to S_3_•^−^ radicals (≈280–310 cm^−1^) further suggest poorly regulated intermediate formation [38, 39]. In contrast, the CD‐CANF cell induces a markedly different sulfur conversion pathway. The long‐chain LiPS bands decay rapidly, accompanied by the transient emergence of well‐defined short‐chain LiPS signals, evidencing accelerated and orderly LiPS reduction. The S_3_•^−^‐related feature becomes more pronounced yet temporally confined, consistent with stabilized radical intermediates under catalytic control. Meanwhile, the Li_2_S_2_/Li_2_S‐related band appears earlier and remains spatially uniform across discharge, indicative of facilitated and homogeneous solid‐state precipitation. Overall, the Raman evolution reveals a smooth and continuous solid‐liquid‐solid sulfur conversion enabled by CD‐CANF. These in situ Raman results provide spectroscopic evidence that the diffusion‐regulated distribution of Co–N_x_ single‐atom sites in CD‐CANF influecnes sulfur speciation at different reaction stages. By accelerating long‐chain LiPS consumption, stabilizing reactive intermediates, and promoting uniform Li_2_S formation, the CD‐CANF suppresses LiPS accumulation and mitigates heterogeneous precipitation, in full agreement with the reduced polarization, enhanced kinetics, and superior cycling stability observed electrochemically. These results suggest that the diffusion‐regulated catalytic architecture improves catalytic accessibility and homogenizes the spatial reaction environment of LiPS conversion across the interlayer.

To further elucidate the interfacial kinetic regulation enabled by the CD‐CANF interlayer, the evolution of the GITT‐derived IR drop was analyzed and correlated with the voltage relaxation behavior (Figure S31a) and the apparent Li^+^ diffusion characteristics (Figure S31c) [40]. As shown in Figure S31b, the PE cell exhibits a pronounced and localized IR drop during discharge–charge processes, indicative of severe polarization and inefficient interfacial charge transfer associated with sluggish polysulfide conversion. In contrast, the CD‐CANF cell displays a markedly suppressed and more uniformly distributed IR drop over the entire reaction trajectory, reflecting a fundamentally altered interfacial environment with reduced energy dissipation. This behavior is fully consistent with the smoother voltage evolution and reduced polarization hysteresis observed in Figure S31a. Moreover, the consistently higher apparent Li^+^ diffusion coefficients of CD‐CANF indicate that the diminished IR drop originates from accelerated interfacial charge transfer and more continuous ionic transport pathways, rather than from transient kinetic effects. Collectively, these results demonstrate that the contra‐diffusion‐engineered CD‐CANF establishes a kinetically stabilized interface that effectively mitigates resistance accumulation during sulfur redox, thereby sustaining reversible polysulfide conversion and long‐term cycling durability.

To rationalize the experimentally observed kinetic advantages, density functional theory (DFT) calculations were performed to probe the interaction between sulfur species and Co–N_x_ catalytic sites (Figure 7d,e). As shown in Figure 7d, atomically dispersed Co–N_x_ centers exhibit progressively enhanced yet moderate adsorption toward sulfur intermediates along the S_8_→Li_2_S reduction pathway. This balanced adsorption behavior enables effective immobilization of soluble LiPSs without imposing excessive energetic penalties on subsequent conversion steps. Correspondingly, the reaction energy diagram in Figure 7e reveals that Co–N_x_ sites substantially lower the energy barriers for LiPS conversion compared with pristine graphene, particularly stabilizing the critical Li_2_S_2_→Li_2_S transformation. This energetically favorable landscape provides a direct mechanistic basis for the accelerated Li_2_S precipitation, reduced polarization, and enhanced reversibility observed experimentally. Taken together, these theoretical and experimental results highlight the essential role of atomically dispersed Co–N_x_ motifs in simultaneously regulating LiPS adsorption and conversion energetics, and establish a general interfacial design principle for constructing durable, high‐performance Li–S batteries.

## 3 Conclusion

In summary, we report a CD‐CANF that enables efficient and durable sulfur redox regulation in Li–S batteries. In contrast to conventional surface‐limited growth, the diffusion‐regulated growth enforces uniform nucleation throughout the aramid nanofiber scaffold, yielding homogeneously dispersed Co–N_x_ single‐atom sites while preserving continuous electronic and ionic transport pathways. As a result, CD‐CANF markedly suppresses polarization and accelerates interfacial charge‐transfer kinetics, leading to improved rate capability and long‐term cycling stability. Potentiostatic Li_2_S precipitation and dissolution measurements further demonstrate that atomically dispersed Co sites simultaneously lower the nucleation barrier for Li_2_S formation and promote its efficient decomposition, enabling effective bidirectional regulation of sulfur redox reactions. These macroscopic performance gains are consistently supported by spectroscopic analyses and theoretical calculations, which reveal optimized interfacial binding and moderated reaction energetics at the Co–N_x_ sites. Notably, comparative landscape analysis against state‐of‐the‐art interlayer systems reveals that CD‐CANF achieves a favorable combination of low metal loading and exceptional cycling stability, highlighting the efficiency of single‐atom utilization enabled by the contra‐diffusion architecture. Beyond Li–S batteries, this work provides a useful design strategy for integrating atomic‐scale catalytic centers into fibrous ion‐conducting frameworks, providing a versatile platform for regulating complex multistep electrochemical reactions in advanced energy‐storage systems.

## 4 Experimental Section

### 4.1 Synthesis of Zn‐Co ZIF‐8 Precursor

Zinc nitrate hexahydrate and cobalt nitrate hexahydrate were dissolved in methanol, followed by rapid mixing with a methanolic solution of 2‐MIm under vigorous stirring. The reaction was maintained at room temperature for 24 h, after which the precipitated bimetallic Zn‐Co ZIF‐8 precursor was collected, washed with methanol, and dried at 60°C overnight.

### 4.2 Preparation of Co Single‐Atom Catalyst (CoSAs/NC)

The Zn‐Co ZIF‐8 precursor was pyrolyzed under flowing N_2_ by heating to 910°C at 5°C min^−^^1^ and holding for 2 h. During carbonization, volatile Zn species were removed, while Co atoms were stabilized by nitrogen coordination within the carbon matrix, yielding Co SACs supported on N‐doped carbon.

### 4.3 Fabrication of Aramid Nanofiber (ANF) Membranes

ANF membranes were prepared *via* a dry‐wet phase inversion process derived from a sol‐gel transition of deprotonated poly(*p*‐phenylene terephthalamide) (PPTA, Kevlar 49). The resulting ANF slurry was blade‐cast onto glass substrates and immersed in deionized water to induce phase inversion, followed by solvent exchange and thermal pressing to obtain free‐standing porous ANF membranes [15].

### 4.4 Preparation of Carbonized ANF (CANF)

ANF membranes were carbonized under N_2_ using a stepwise heating program up to 910°C to obtain conductive carbonized ANF (CANF), which was directly used as an interlayer.

### 4.5 Preparation of Interlayers via Sink‐Growth Method (S‐CANF)

For sink growth, ANF membranes were immersed in a 2‐MIm solution, followed by dropwise addition of a mixed Zn^2^^+^/Co^2^^+^ solution. After in‐situ ZIF growth, the membranes were thermally pressed and carbonized to obtain sink‐derived carbonized interlayers (S‐CANF).

### 4.6 Preparation of Interlayers via Contra‐Diffusion Method (CD‐CANF)

For contra‐diffusion growth, ANF membranes were mounted in an H‐type cell separating metal‐ion and ligand solutions. Controlled bidirectional diffusion enabled confined ZIF growth within the membrane interior. After thermal pressing and carbonization, contra‐diffusion‐derived carbonized interlayers (CD‐CANF) were obtained.

## Author Contributions

**Yun‐Sheng Ye**: conceptualization; validation; data curation; supervision; funding acquisition; visualization; resources; writing – review and editing; project administration. **Tsung‐I Yeh**: methodology;investigation;formal analysis. **Yan‐Jhang Chen**: methodology; investigation; validation; formal analysis; data curation; writing – original draft. **Bing‐Joe Hwang**: supervision; resources; funding acquisition. **Wei‐Ming Huang**: investigation; formal analysis. **Mohamed Gamal Mohamed**: investigation; formal analysis. **Shiao‐Wei Kuo**: supervision, resources. **Jing‐Yu Li**: investigation; formal analysis. **Chia‐Yu Chang**: investigation; formal analysis.

## Conflicts of Interest

The authors declare no conflicts of interest.

## Supporting information




**Supporting File 1**: anie72359‐sup‐0001‐SuppMat.docx.

## Data Availability

The data that support the findings of this study are available from the corresponding author upon reasonable request.
