# Supplementary material for: Contra‐Diffusion Engineering of Single‐Atom Catalytic Interlayers Enables Reversible Sulfur Redox Chemistry
Source: Angew Chem Int Ed Engl. 2026 Apr 27;65(24):e7009531. doi: 10.1002/anie.7009531 (PMC13245605; doi:10.1002/anie.7009531)
Supplement: Supplementary file 1 — Supporting File 1: anie72359‐sup‐0001‐SuppMat.docx. [file ANIE-65-e7009531-s001.docx]

Contra-Diffusion Engineering of Single-Atom Catalytic Interlayers Enables Reversible Sulfur Redox Chemistry

Yan-Jhang Chen, Tsung-I Yeh, Chia-Yu Chang, Wei-Ming Huang, Jing-Yu Li, Mohamed Gamal Mohamed, Shiao-Wei Kuo, Bing-Joe Hwang, and Yun-Sheng Ye*

**S1. Materials**

Zinc nitrate hexahydrate (Zn(NO₃)₂·6H₂O, ≥99.0%), cobalt(II) nitrate hexahydrate (Co(NO₃)₂·6H₂O, ≥98.0%), and 2-methylimidazole (2-MIm, ≥99.0%) were purchased from commercial suppliers and used as received. Methanol (MeOH, anhydrous) was used as the solvent for ZIF precursor synthesis. Commercial poly(p-phenylene terephthalamide) (PPTA) (Kevlar® 49, DuPont) fibers were employed as the starting material for aramid nanofiber (ANF) preparation. Potassium tert-butoxide (KOtBu) and dimethyl sulfoxide (DMSO, anhydrous) were used to deprotonate and disperse PPTA into aramid nanofibers. Deionized water, methanol, and n-hexane were used sequentially for phase inversion and solvent exchange during membrane fabrication. Carbon nanotubes (CNTs, L/D ~ 1000, ≥99.9 wt. % MWCNT basis) and super P were used as conductive substrates for Li₂S₆/Li₂S₈ loading in symmetric-cell and Li₂S nucleation/dissolution tests. Poly(vinylidene fluoride) (PVDF) was used as the polymer binder for electrode fabrication, and N-methyl-2-pyrrolidone (NMP) was employed as the solvent for slurry preparation. Lithium bis(trifluoromethanesulfonyl)imide (LiTFSI) and lithium nitrate (LiNO₃) were used as electrolyte salts. 1,3-dioxolane (DOL) and 1,2-dimethoxyethane (DME) were used as electrolyte solvents. Lithium metal foil (battery grade) served as the counter/reference electrode. Commercial polyethylene (PE) separators were used as control interlayers. All materials were used as received without further purification unless otherwise specified.

**S2. Experimental section**

**S2.1. Detailed synthesis of Zn-Co ZIF-8.** Cobalt(II) nitrate hexahydrate (0.32 g, 1.10 mmol) and zinc nitrate hexahydrate (2.61 g, 8.77 mmol) were dissolved in 200 mL of methanol to form solution A. Separately, 2-MIm (6.52 g, 79.40 mmol) was dissolved in 200 mL of methanol as solution B. Solution B was poured into solution A under vigorous stirring, and the mixture was stirred at room temperature for 24 h. The resulting precipitate was collected by centrifugation, washed repeatedly with methanol, and dried at 60 °C overnight.

**S2.2. Detailed preparation of CoSAs/NC.** The Zn-Co ZIF-8 precursor was placed in a tubular furnace and heated to 910 °C at a ramping rate of 5 °C mim^-1^ under flowing high-purity N₂. The temperature was maintained for 2 h to ensure complete carbonization and Zn evaporation. After natural cooling, the obtained black powder was denoted as CoSAs/NC.

**S2.3. Detailed fabrication of ANF membranes.^[1]^** Commercial PPTA fibers were deprotonated using potassium tert-butoxide (KOtBu) under strongly alkaline conditions to form aramid nanofibers, which were subsequently dispersed in DMSO. The dispersion was stirred until homogeneous and heated at 70 °C for 1 h to obtain a viscous ANF slurry. The slurry was cast onto a glass plate using a doctor blade and immersed in deionized water at 60 °C to induce phase inversion. The membrane was sequentially washed with DI water, methanol, and n-hexane to remove residual solvents and dried by thermal pressing at 120 °C.

**S2.4. Carbonization of ANF membranes.** ANF membranes were punched into 16 mm disks, sandwiched between quartz plates, and carbonized under N₂ using a multistep heating protocol: 350 °C, 650 °C, and 910 °C, each held for 2 h with a heating rate of 5 °C min⁻¹.

**S2.5. Sink-growth ZIF formation on ANF (S-CANF).** ANF membranes were immersed in 2-MIm solution, followed by controlled dropwise addition of Zn²⁺/Co²⁺ precursor solution over 1 h. After reaction, membranes were thermally pressed and carbonized to yield S-CANF.

**S2.6. Contra-diffusion ZIF formation on ANF (CD-CANF).** ANF membranes were mounted in an H-type diffusion cell using rubber gaskets. Metal-ion and ligand solutions were introduced into opposite compartments, allowing bidirectional diffusion and confined ZIF growth. After reaction, membranes were thermally pressed, trimmed, and carbonized to obtain CD-CANF.

**S2.7. Preparation of Li₂S₆/Li₂S₈ solution.** Li₂S and sulfur powder were mixed in molar ratios of 1:5 and 1:7, respectively, and dissolved in a 1:1 v/v mixture of DOL and DME. The mixtures were sealed and heated at 70 °C under constant stirring for 24 h to ensure complete dissolution and equilibration. After cooling to room temperature, the solutions were centrifuged to remove any undissolved sulfur. The resulting clear dark-brown supernatants were collected as Li₂S₆ and Li₂S₈ stock solutions, respectively, and stored in an argon-filled glovebox to prevent oxidation and moisture contamination.

**S2.8. Preparation of sulfur and CNT control cathodes.** After completely dissolving 0.8 g of PVDF in 56 mL of anhydrous NMP, thoroughly grind 4.8 g of sulfur powder and 2.4 g of CNT in a mortar. Then add the mixed powder into the NMP solution and sonicate for 1 hour to obtain a preliminary mixed slurry. Move the slurry to an ARE-310 planetary centrifugal mixer and stir at 2000 rpm for 1 h to obtain a uniformly mixed high-viscosity slurry. The slurry was subsequently spread onto aluminum foil using blades of different thicknesses, dried overnight at 70 °C, and then moved to a vacuum oven at 50 °C for 8 h. The sulfur loading on the electrode substrates utilized in the electrochemical tests for this study varied from approximately 1.5, 4, and 6 mg cm^-2^.

**S2.9. Preparation of Co-SAs/NC-coated PE separator.** To prepare the Co-SAs/NC-coated separator (Co-Sas/NC@PE), Co-SAs/NC powder, Super P conductive carbon, and PVDF binder were mixed in a mass ratio of 8:1:1. The mixture was dispersed in NMP to form a homogeneous slurry under magnetic stirring. The slurry was then blade-coated onto a commercial PE separator to form a thin catalytic layer. After coating, the separators were dried at 60 °C under vacuum overnight to remove residual solvent. The resulting catalytic coating exhibited an average thickness of ~6 μm with a catalyst loading of ~1.1 mg cm^-2^. The coated separators were directly used for electrochemical measurements as control samples.

**S3. Characterization**

Fourier-transform infrared (FTIR) spectra were recorded on a Bruker Tensor 27 spectrometer using KBr pellets with a resolution of 4 cm^-1^ and 128 scans. X-ray photoelectron spectroscopy (XPS) was performed on a Thermo Fisher Scientific ESCALAB 250 instrument with a resolution of 8-20 eV. Thermogravimetric analysis (TGA) was carried out on a TA Instruments Q50 under nitrogen flow (60 mL min^-1^), heating from room temperature to 800 °C at a rate of 10 °C min^-1^. Electrochemical measurements were performed with a CH Instruments 600E workstation. Galvanostatic charge/discharge cycling was conducted on a LANHE CT3002A battery tester.

**S4. Electrochemical analysis**

**S4.1. Li₂S₆ symmetric cell tests**

To evaluate the intrinsic catalytic activity of different interlayers toward lithium polysulfide (LiPS) conversion, symmetric cells were assembled using CNT and CoSAs/NC electrodes in combination with various interlayers. Model electrodes were prepared by uniformly drop-casting a quantified amount of Li₂S₆ solution onto CNT or CoSAs/NC electrodes, followed by vacuum drying to remove residual solvent. The Li₂S₆ solution (0.2 M) was prepared in a DOL/DME mixture (1:1 v/v) containing 1 M LiTFSI, and the loading amount was strictly controlled to ensure identical polysulfide content for all tests. This quantitative immobilization of Li₂S₆ enables a direct comparison of catalytic contributions from different interlayers.

CR2032-type symmetric cells were assembled in an argon-filled glovebox (H₂O, O₂ < 0.1 ppm) using two identical electrodes with different configurations depending on the presence of interlayers. For the control group, Li₂S₆ solution was quantitatively loaded onto CNT electrodes, and symmetric cells were constructed using CNT‖CNT electrodes with a commercial PE separator. For interlayer-assisted configurations, the Li₂S₆ solution was directly and quantitatively loaded onto the interlayer membranes (CANF, S-CANF, or CD-CANF), which thus served as the active electrodes. Two identical Li₂S₆-loaded interlayers were then assembled face-to-face, with a PE membrane placed between them as the separator. In all cases, no additional liquid electrolyte was introduced into the cells.

Galvanostatic cycling and cyclic voltammetry (CV, 2.0-2.8 V vs. Li/Li⁺) were performed at 30 °C to probe the redox kinetics of immobilized LiPSs. The peak current response, peak separation, and polarization behavior were used to evaluate the catalytic efficiency of different interlayers in promoting LiPS conversion.

**S4.2. Li₂S precipitation/dissolution tests**

The Li₂S nucleation and dissolution behaviors were investigated by potentiostatic deposition and stripping measurements using Li₂S₈ as the sulfur precursor.

(i) Intrinsic catalytic activity of CNT and CoSAs/NC electrodes: To probe the intrinsic catalytic effect of atomically dispersed Co sites, CNT and CoSAs/NC electrodes were first impregnated with a quantified amount of Li₂S₈ solution (0.2 M in DOL/DME, 1:1 v/v) and vacuum-dried to immobilize Li₂S₈ within the porous electrode framework. CR2032-type cells were assembled in an argon-filled glovebox (H₂O, O₂ < 0.1 ppm) using the Li₂S₈-loaded CNT or CoSAs/NC as the cathode, lithium metal foil as the anode, and a commercial PE membrane as the separator. A liquid electrolyte consisting of DOL/DME (1:1 v/v) with LiNO₃ additive was added, with an electrolyte-to-sulfur (E/S) ratio of 20:1. Potentiostatic discharge was conducted at a fixed potential of 2.05 V vs. Li/Li⁺ until the current decayed to a stable baseline, corresponding to complete Li₂S deposition.

(ii) Interlayer-assisted Li₂S nucleation and dissolution tests: To evaluate the role of interlayers in regulating Li₂S nucleation under identical cathode chemistry, a Li₂S₈-loaded CNT cathode (prepared with the same quantified Li₂S₈ loading) was employed for all interlayer-assisted cells. The interlayer membrane (CANF, S-CANF, or CD-CANF) was placed between the cathode and the PE separator, followed by lithium metal foil as the anode. The same DOL/DME-based electrolyte with LiNO₃ was added at an E/S ratio of 20:1. Potentiostatic Li₂S deposition was carried out at 2.05 V vs. Li/Li⁺ under otherwise identical conditions. For both configurations, the induction time, nucleation current, and total Li₂S deposition capacity were extracted to quantify the kinetics of solid Li₂S formation. After deposition, Li₂S dissolution was examined by reversing the potential, and the corresponding oxidation capacity and current decay behavior were recorded to assess the solid-to-liquid conversion kinetics. All measurements were conducted at 30 °C.

**S4.3. Electrochemical evaluation of interlayers**

***Cell assembly.*** CR2032-type coin cells were assembled in an argon-filled glovebox (H₂O, O₂ < 0.1 ppm) using a conventional Li-S battery configuration. S-cathodes were prepared by coating sulfur composite onto aluminum foil, while Li metal foil served as both the counter and reference electrode. PE membranes were used as separators. For interlayer-modified cells, CANF, S-CANF, or CD-CANF membranes (~35 μm, ~1.8 mg) were inserted between the S-cathode and the PE separator as functional interlayers. For comparison, cells without interlayers (PE only) and using the Co-SAs/NC@PE-coated separator were also tested under the same conditions. The electrolyte consisted of DOL/DME (1:1 v/v) with LiNO₃ additive, and the electrolyte-to-sulfur (E/S) ratio was fixed at 20:1 for all cells to ensure a fair comparison. After assembly, the cells were rested for 12 h to allow sufficient electrolyte infiltration and interfacial equilibration. Unless otherwise specified, all electrochemical measurements were conducted at 30 °C.

***Rate capability test.*** Rate capability measurements were carried out to evaluate the kinetic advantages imparted by different interlayers. Galvanostatic charge-discharge tests were performed at progressively increasing current rates, as shown in **Figure 6e**, while maintaining identical electrode configurations and sulfur loadings. Each current rate was applied for a fixed number of cycles before switching to the next rate, and the reversibility of capacity was examined by returning to a lower current density at the end of the test. The resulting discharge capacities and voltage profiles were used to assess polarization behavior and reaction kinetics under dynamic operating conditions.

***Long-term cycling stability.*** Long-term cycling performance was evaluated at a constant current rate of 2 C to probe the durability of the interlayers under prolonged operation. As presented in **Figure 6i**, the cells assembled with the CD-CANF interlayer were continuously cycled for up to 2000 cycles. For comparison, cells using the Co-SAs/NC@PE-coated separator were also tested under the same conditions. The specific capacity retention and Coulombic efficiency were monitored throughout the test to quantify capacity decay and electrochemical stability.

***Polarization analysis.*** Voltage polarization was analyzed from the galvanostatic charge-discharge profiles. The potential gap between charge and discharge at defined states of charge (e.g., 50% depth of discharge) was extracted to compare the overpotential induced by different interlayers under identical current conditions.

***Post-cycling characterization.*** After electrochemical testing, selected cells were disassembled in the glovebox. The electrodes and interlayers were retrieved, gently rinsed with anhydrous solvent to remove residual electrolyte, and dried prior to further structural and surface analyses.

**S4.4. Ionic conductivity (*σ*).** The *σ* of the PE with different interlayers was measured by electrochemical impedance spectroscopy (EIS) using stainless steel blocking electrodes.

$\sigma=\frac{d}{R_{b}\times S}$ (S1)

where *d* is the electrolyte thickness (cm), *S* is the electrode/electrolyte contact area (cm²), and *R_b_* is the bulk resistance obtained from the Nyquist plot intercept at high frequency.

**S4.5. Li⁺ transference number (**$\boldsymbol{t}_{\boldsymbol{Li}^{\boldsymbol{+}}}$**), Bruce-Vincent-Evans method).^[2]^**

$t_{{Li}^{+}=}\frac{I_{S}(\Delta V-I_{0}R_{0})}{I_{0}(\Delta V-I_{S}R_{S})}$ (S2)

where *I_0_* and *I_s_* are the initial and steady-state currents, *ΔV* is the applied potential, and *R_0_* and *R_s_* are the interfacial resistances before and after polarization, respectively.

**S4.6. Randles–Sevcik equation for Li⁺ diffusion coefficient.^[3]^**

The Li⁺ diffusion coefficient ($D_{{Li}^{+}}$) was determined from cyclic voltammetry (CV) using the Randles–Sevcik equation:

$I_{p}=(2.69\times{10}^{5})n^{3/2}AD^{1/2}Cv^{1/2}$ (S3)

where *I_p_* is the peak current (A), *n* is the number of electrons transferred, *A* is the electrode surface area (cm²), *D* is the Li⁺ diffusion coefficient (cm² s⁻¹), *C* is the concentration of the active species in the electrolyte (mol cm^-3^), and *v* is the scan rate (V s⁻¹).

A linear relationship between *I_p_* and *v^1/2^* confirms diffusion-controlled kinetics, and the slope of the plot was used to calculate $D_{{Li}^{+}}$ for different electrolyte systems.

**S4.7. Li⁺ diffusion coefficient (GITT method).^[4]^**

$D_{{Li}^{+}}=\frac{4}{\pi\tau}{(\frac{m_{B}V_{M}}{M_{B}S})}^{2}{(\frac{\Delta E_{S}}{\Delta E_{\tau}})}^{2}$ (S4)

where *τ* is the current pulse duration, *m_B_* is the mass of active material, *V_M_* is the molar volume, *M_B_* is the molar mass, *S* is the electrode/electrolyte interface area, *ΔE_τ_* is the potential change during the current pulse (excluding *IR* drop), and *ΔE_S_* is the steady-state voltage change after relaxation.

**S4.8. Tafel slope analysis.^[5]^ T**afel slopes were derived from CV using:

$\eta=a+b log(i)$ (S5)

where *η* is the overpotential, *i* is the current density, and *b* is the Tafel slope (mV dec⁻¹). The smaller the slope, the faster the interfacial redox kinetics and charge-transfer processes.

**S5. In-situ Raman spectroscopy**

In situ Raman measurements were carried out to monitor the sulfur speciation evolution during galvanostatic discharge of Li-S cells equipped with either a pristine PE separator or the CD-CANF interlayer. A customized optically transparent in situ cell was employed, allowing direct laser access to the cathode surface while maintaining an electrochemically sealed environment.

Raman spectra were collected using a confocal Raman microscope equipped with a 532 nm excitation laser. The laser power was carefully controlled (<1 mW at the sample surface) to minimize local heating and avoid laser-induced sulfur degradation. Spectra were acquired in the range of 150-800 cm^-1^ with an acquisition time of 5 min per spectrum. The laser spot was focused on a fixed position of the cathode throughout the entire discharge process to ensure spatial consistency.

Galvanostatic discharge was performed at 0.5 C between 1.5 and 3 V (vs. Li/Li⁺), and Raman spectra were continuously recorded at defined time intervals. The electrochemical voltage profile was synchronously recorded and subsequently correlated with the Raman spectral evolution. Raman intensity contour maps were generated by normalizing the discharge time and plotting Raman shift as a function of time, enabling visualization of sulfur species evolution throughout the discharge process.

**S6. Computational Methods**

Spin-polarized density functional theory (DFT) calculations were performed using the CP2K package with a mixed Gaussian and plane-wave basis set. Core electrons were treated by norm-conserving Goedecker-Teter-Hutter pseudopotentials, while valence electrons were expanded in a double-ζ basis set with polarization functions and an auxiliary plane-wave cutoff of 450 Ry. The exchange–correlation functional was described by the generalized gradient approximation of Perdew–Burke-Ernzerhof (PBE), together with Grimme’s DFT-D3 dispersion correction. All atomic structures were fully optimized using the Broyden-Fletcher-Goldfarb-Shanno (BFGS) algorithm until the SCF convergence criterion of 1×10^-5^ au was achieved.

The adsorption energy (*E_ads_*) of Li₂Sₓ/S₈ species on the substrate was calculated as:

$E_{ads}=E_{substrate+LiS}-E_{substrate}-E_{LiS}$ (S6)

where $E_{substrate+LiS}$ is the total energy of the adsorption system, $E_{substrate}$ the pristine substrate energy, and $E_{LiS}$ the isolated polysulfide cluster energy. More negative values indicate stronger binding interactions.

The Gibbs free energy of sulfur reduction steps was derived from:

$\Delta G=\Delta E_{DFT}+\Delta E_{ZPE}-T\Delta S$ (S7)

where $\Delta E_{DFT}$ is the reaction energy from DFT, $\Delta E_{ZPE}$ is the zero-point energy correction obtained from vibrational frequencies, and $\Delta S$ is the vibrational entropy contribution evaluated within the harmonic oscillator approximation.

The zero-point energy (ZPE) contribution is given by:

$E_{ZPE}= \sum_{i} \frac{{hv}_{i}}{2}$ (S8)

where $h$ and $v_{i}$ are the Plank’s constant and vibrational frequencies which are calculated based on the localized harmonic oscillator approximation with a displacement of 0.01 Å. Only the mobile ascorbates and the atoms at the active site were considered while all other atoms on the catalyst are treated as fixed. The vibrational entropy contributions ($S$) can be calculated below:

$S=k_{B}\sum_{i} \left( \frac{hv_{i}}{k_{B}T\left( e^{\frac{hv_{i}}{k_{B}T}}-1 \right)}-ln\left( 1-e^{\frac{-hv_{i}}{k_{B}T}} \right) \right)$ (S9)

The equations describing the reduction process of S_8_ to Li_2_S can be presented as follows, where the symbol of star indicates the adsorbed/activated state:

| ${*S}_{8}+16{Li}^{+}+{2e}^{-}\to{*Li}_{2}S_{8}+14{Li}^{+}$ | (S10) |
| --- | --- |
| ${*Li}_{2}S_{8}+14{Li}^{+}+{2e}^{-}\to{*Li}_{2}S_{6}+{Li}_{2}S_{2}+12{Li}^{+}$ | (S11) |
| ${*Li}_{2}S_{6}+{Li}_{2}S_{2}+12{Li}^{+}+{2e}^{-}\to{*Li}_{2}S_{4}+{2Li}_{2}S_{2}+10{Li}^{+}$ | (S12) |
| ${*Li}_{2}S_{4}+{2Li}_{2}S_{2}+10{Li}^{+}+{2e}^{-}\to{*Li}_{2}S_{2}+{3Li}_{2}S_{2}+8{Li}^{+}$ | (S13) |
| ${*Li}_{2}S_{2}+{3Li}_{2}S_{2}+8{Li}^{+}+{8e}^{-}\to{*Li}_{2}S+{7Li}_{2}S$ | (S14) |

**S7. H-cell membrane diffusion model**

The diffusion coefficient was determined based on a classical two-compartment diffusion model, in which two well-stirred reservoirs are separated by a planar membrane. According to Fick’s diffusion theory, the concentration deviation from the equilibrium state decays exponentially with time, and can be expressed as:

$C\left( t \right)-C_{\infty}=(C_{0}-C_{\infty})exp(-\beta t)$ (S15)

where *C(t)* is the concentration at time *t*, $C_{\infty}$ is the equilibrium concentration, and *β* is the overall mass-transfer coefficient. For membrane-controlled diffusion, *β* is defined as:

$\beta=\frac{DA}{l}(\frac{1}{V_{1}}+\frac{1}{V_{2}})$ (S16)

where *D* is the diffusion coefficient, *A* is the membrane area, *l* is the membrane thickness, and *V_1_* and *V_2_* are the volumes of the two compartments. Accordingly, *D* can be calculated from the slope of $ln(C-C_{\infty})$ versus time:

$D=\frac{\beta l}{A(\frac{1}{V_{1}}-\frac{1}{V_{2}})}$ (S17)

This analytical formulation represents the exact solution of Fick’s second law for concentration relaxation between two stirred vessels separated by a membrane and can be found in classical diffusion theory literature.^[6]^


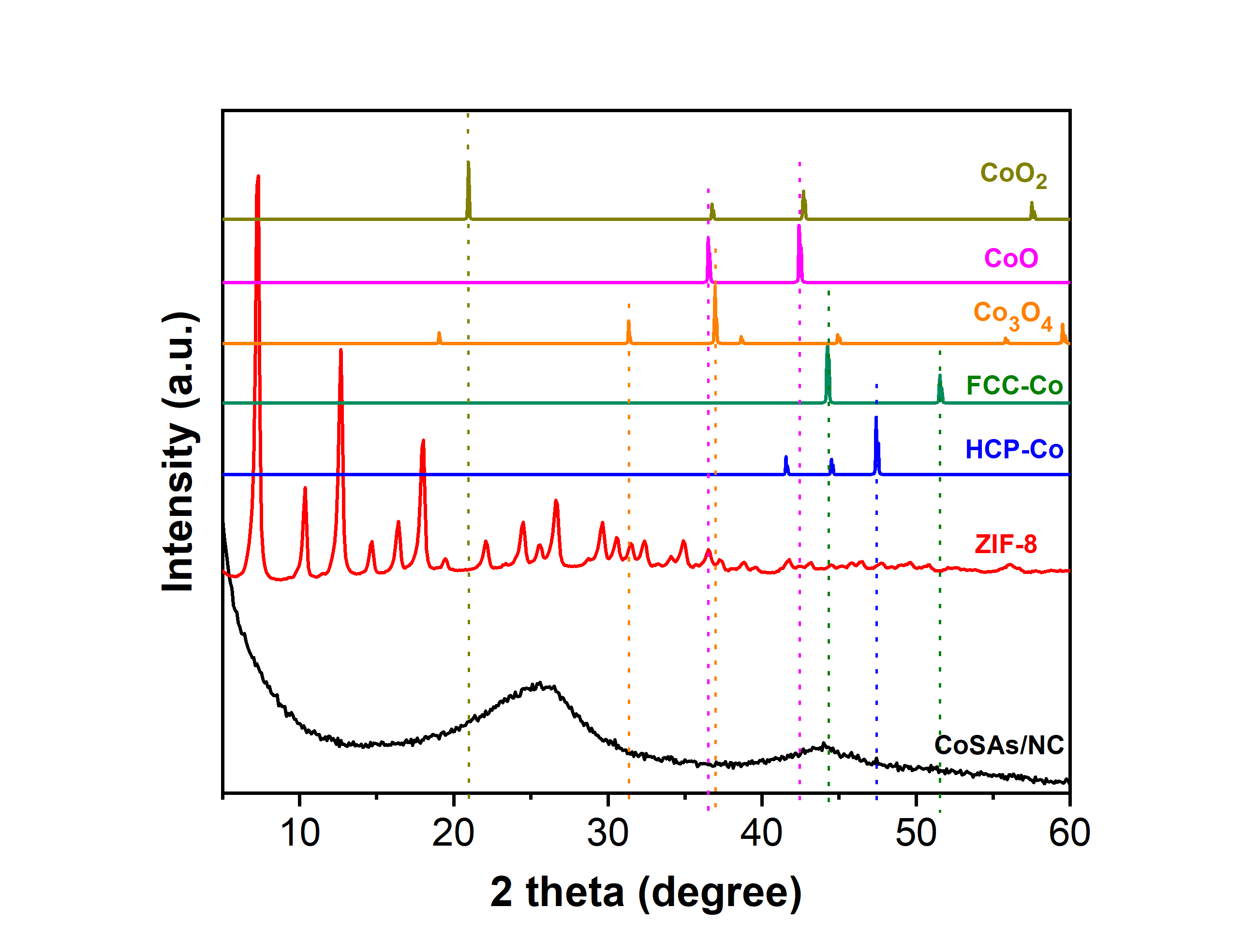


**Figure S1.** XRD patterns of ZIF-8 and CoSAs/NC compared with reference diffraction lines of CoO_2_, CoO, Co_3_O_4_, FCC-Co, and HCP-Co. The ZIF-8 precursor shows the typical sodalite-type reflections, whereas CoSAs/NC displays no detectable crystalline phases corresponding to cobalt metal or cobalt oxides.


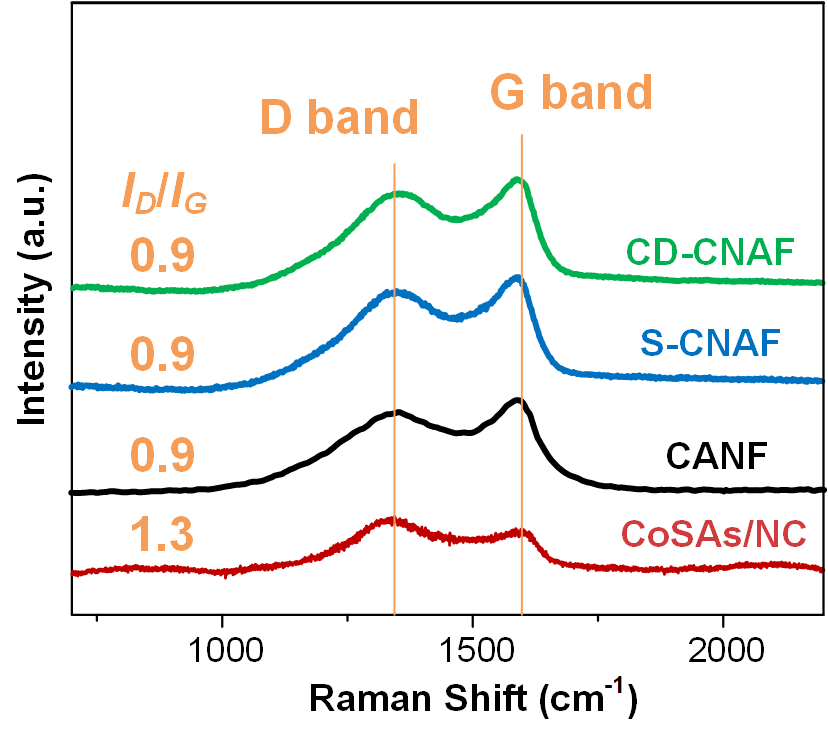


**Figure S2.** Raman spectra of CoSAs/NC, CNAF, S-CANF, and CD-CANF.


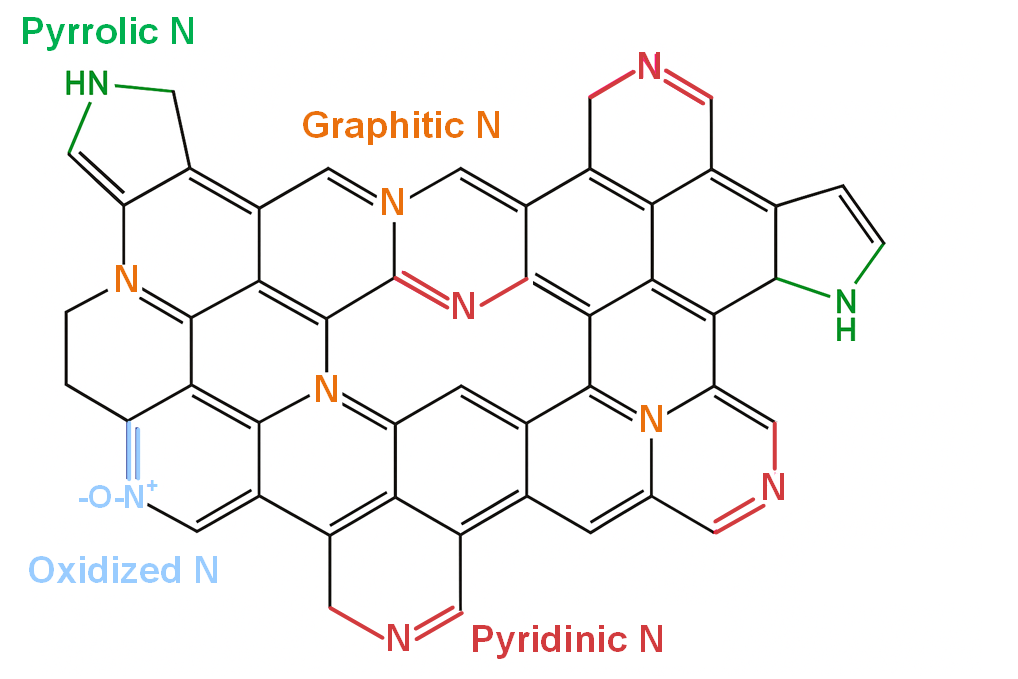


**Figure S3.** Schematic representation of the major nitrogen configurations in N-doped carbon materials, including pyridinic N, pyrrolic N, graphitic N, and oxidized N.

**
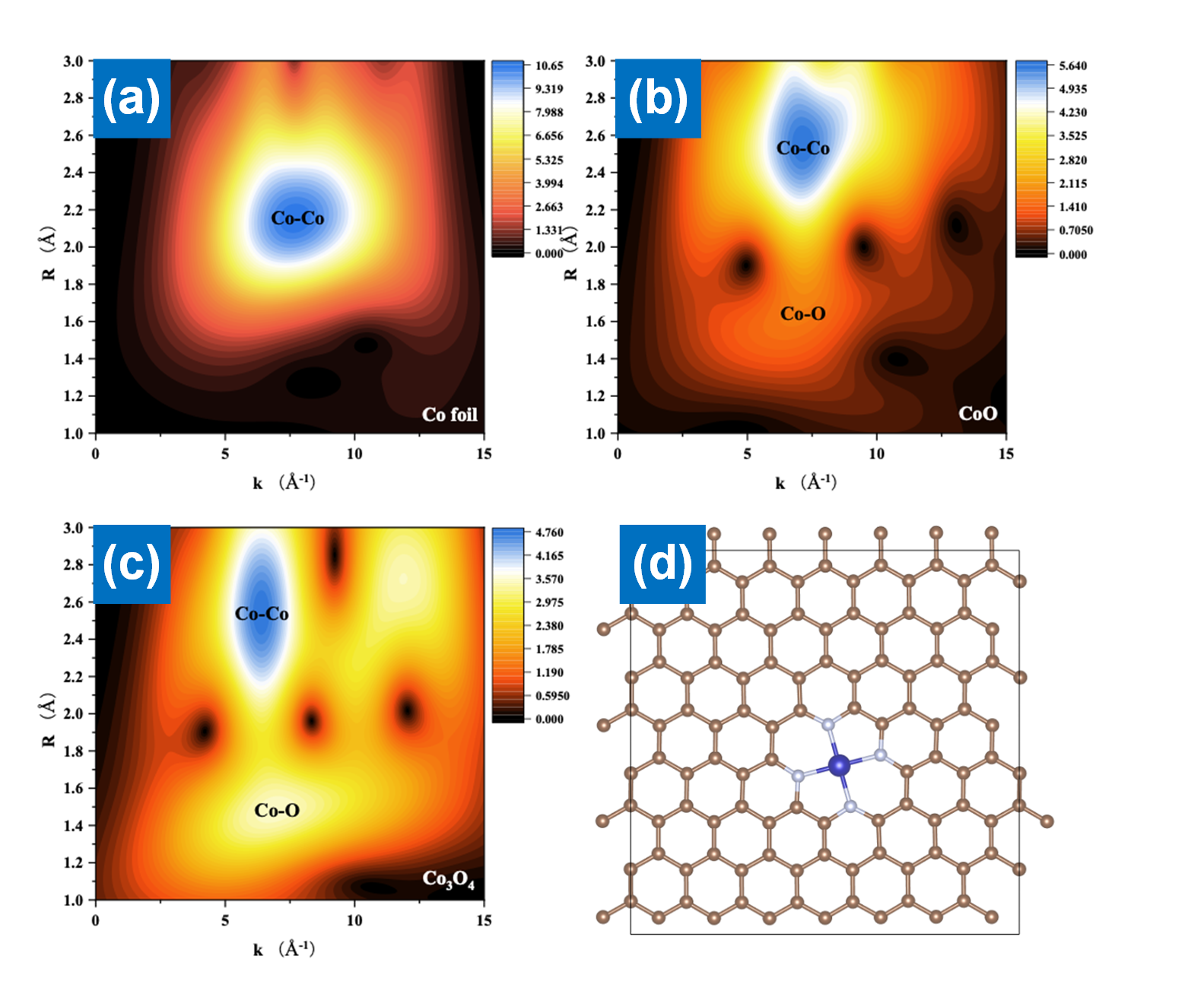
**

**Figure S4.** (a-c) Wavelet transform (WT) contour plots of Co foil (a), CoO (b), and Co₃O₄ (c), showing characteristic Co-Co and Co-O scattering features in the reference materials. (d) Schematic model of a Co-N₄ coordination environment embedded within a carbon matrix.


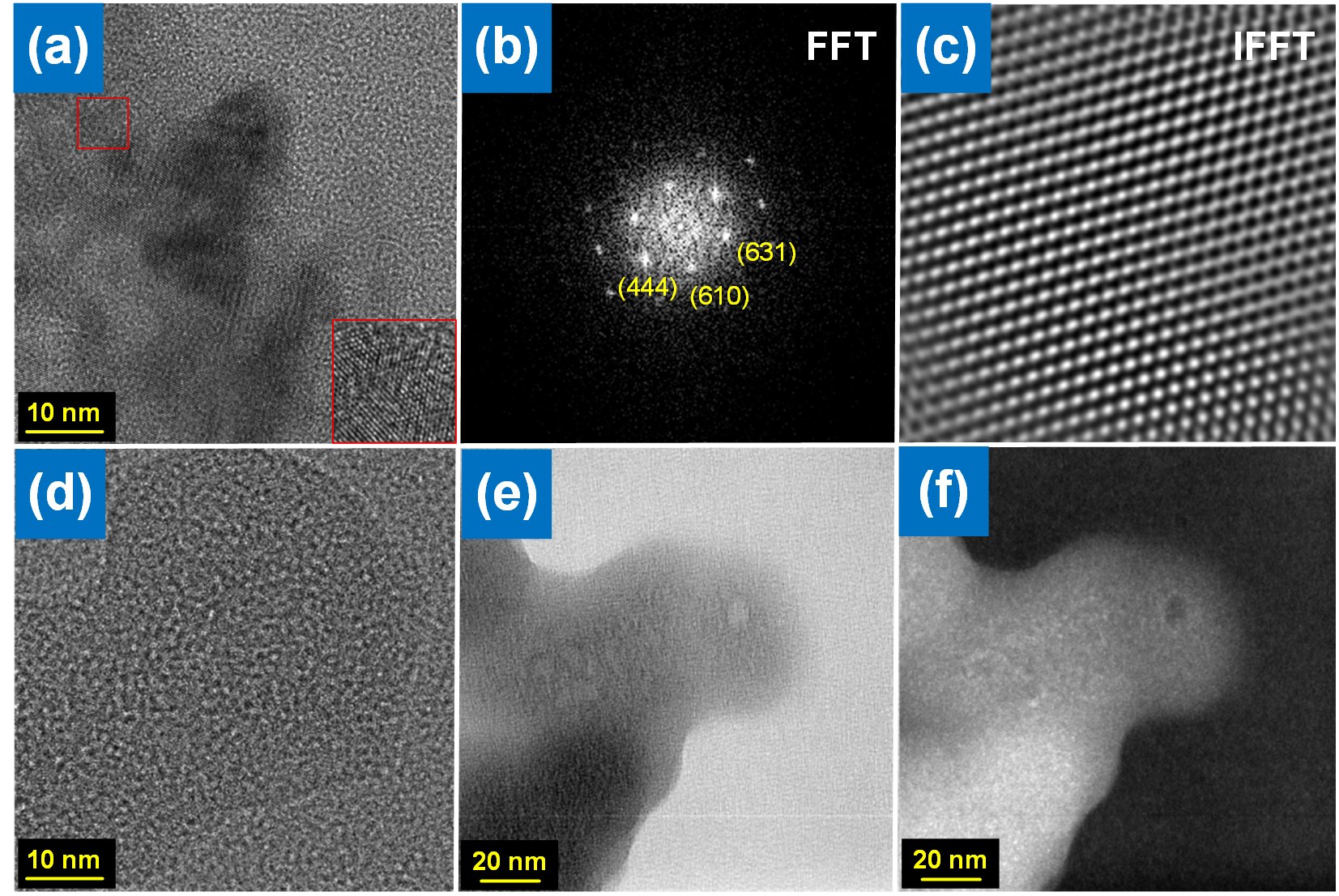


**Figure S5.** (a) HRTEM image, (b) Fast Fourier transform (FFT) pattern, and (c) Lattice fringe image of ZIF-8. (d) HRTEM image, (e) Bright field, and (f) Dark field image of CoSAs/NC.

**
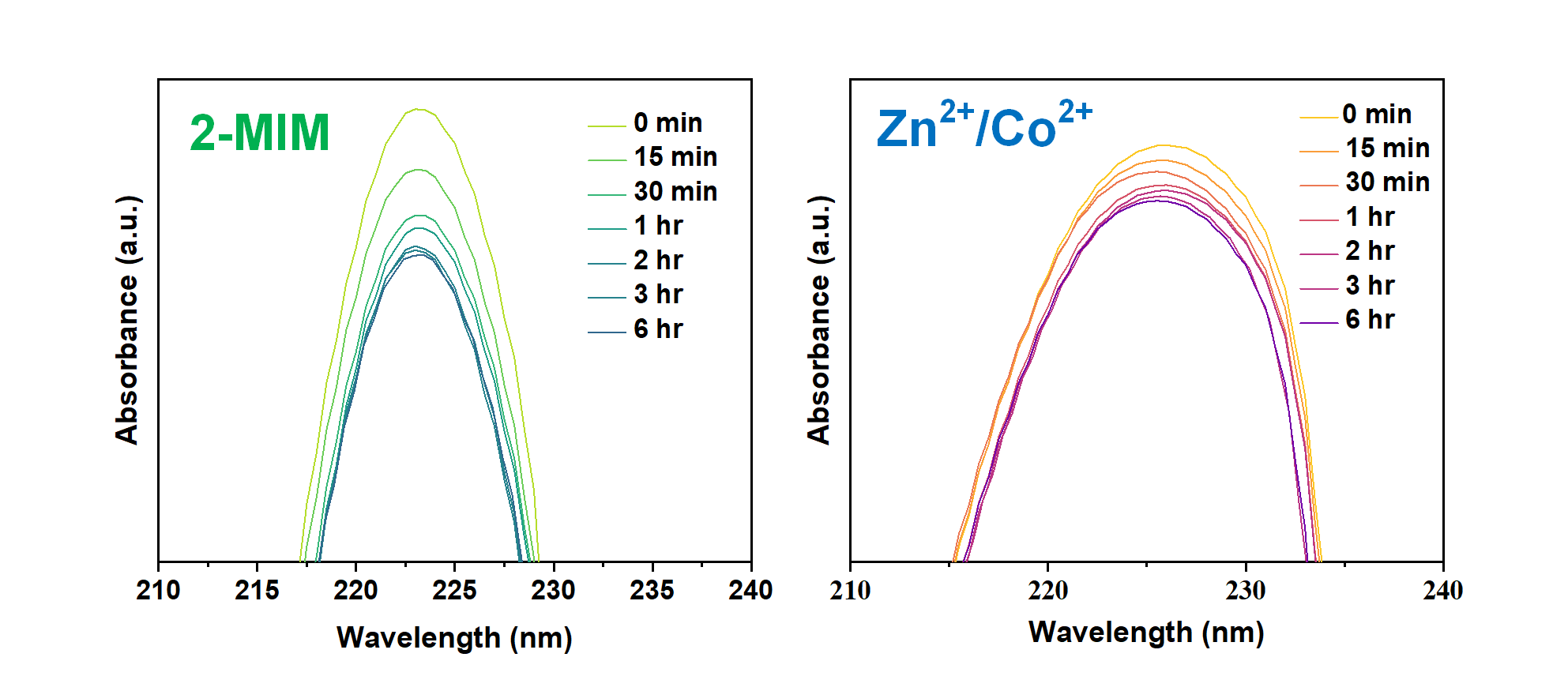
**

**Figure S6.** Time-resolved UV-vis absorption spectra of 2-MIm and Zn²⁺/Co²⁺ solutions during the contra-diffusion process, showing the gradual decrease in reactant concentration over time.


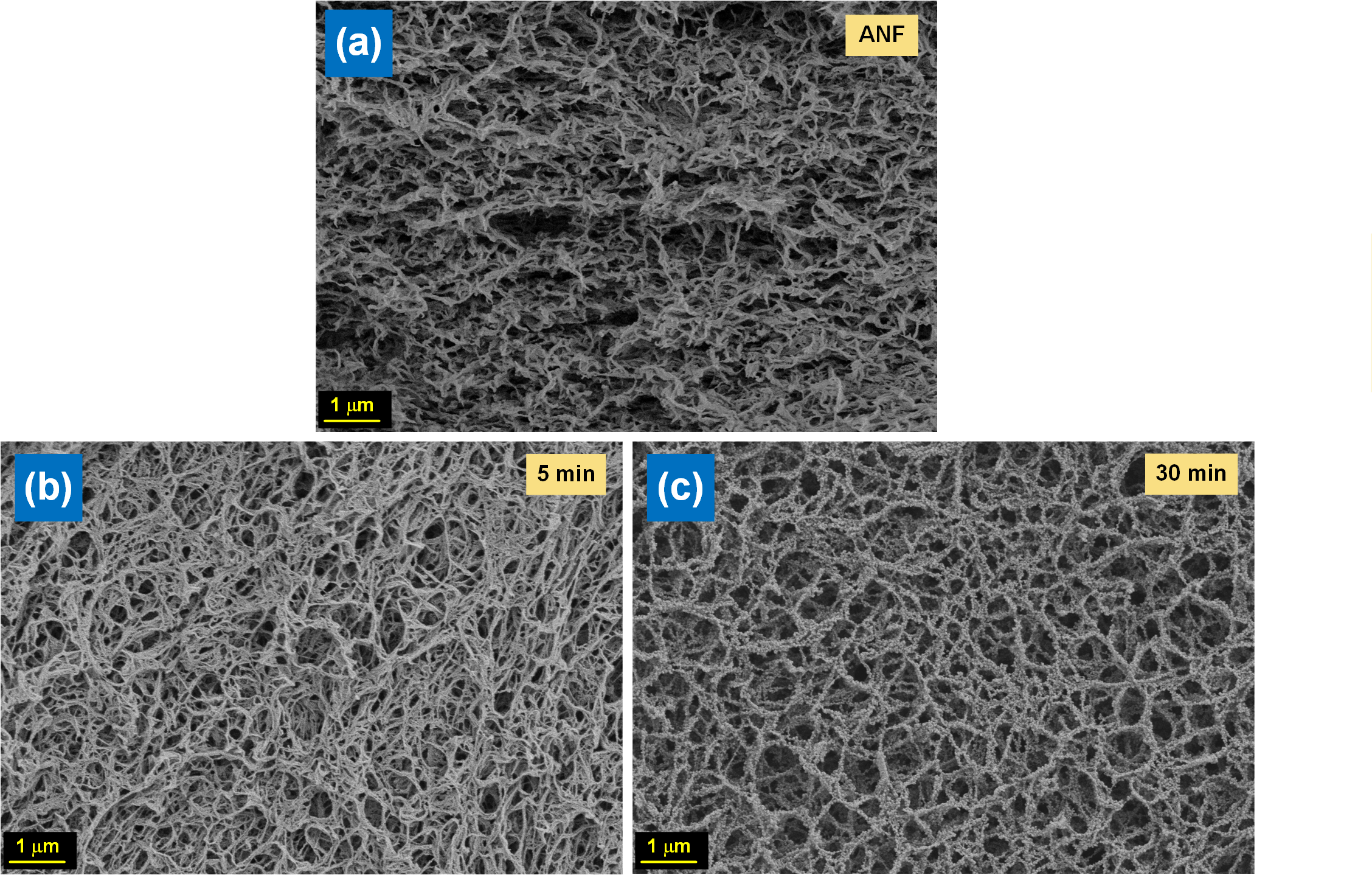


**Figure S7.** SEM images of ANF during contra-diffusion growth at different reaction times

(0, 5, and 30 min).


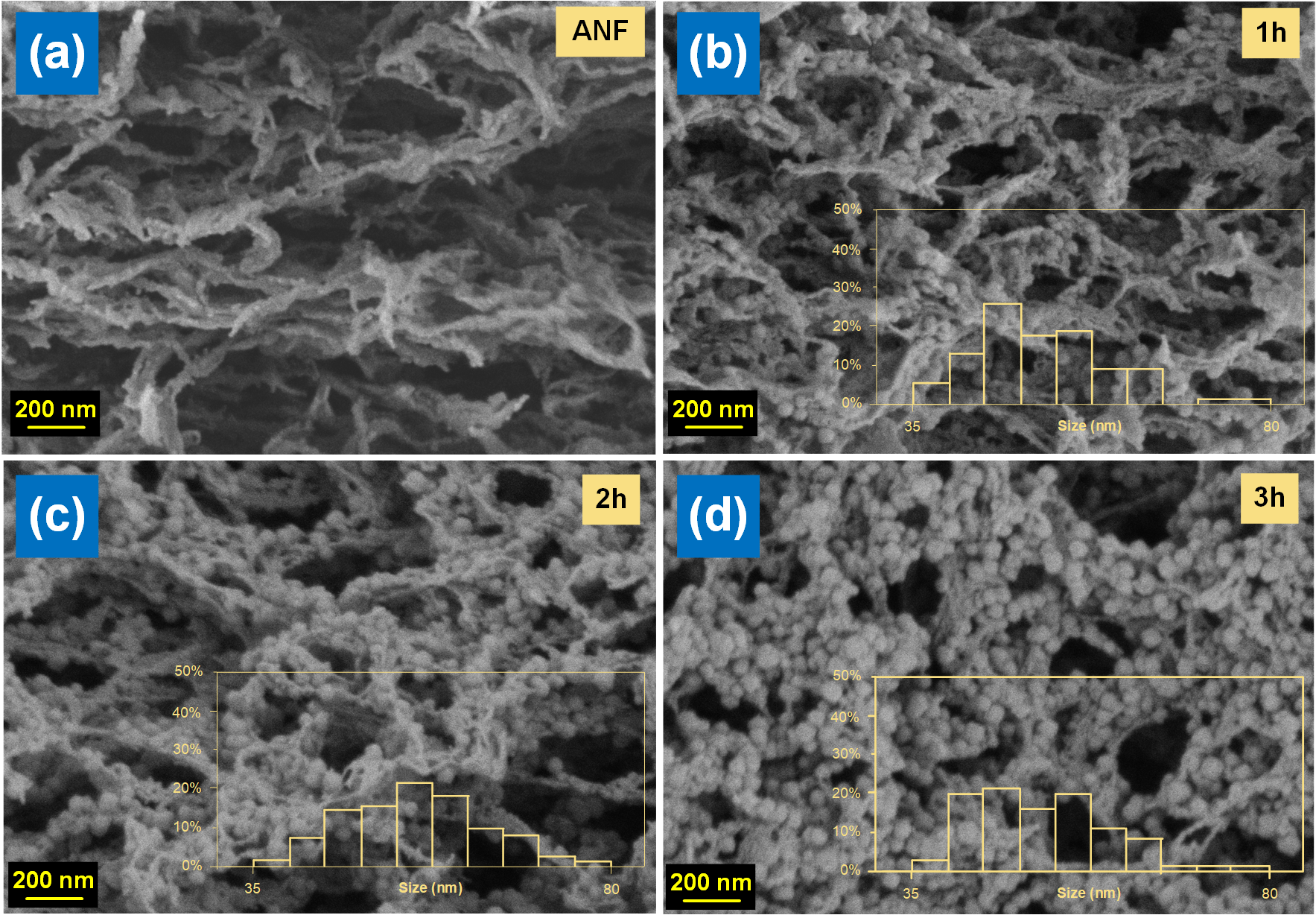


**Figure S8.** SEM images of ANF during contra-diffusion growth at different reaction times (0 h, 1, 2, 3 h), with particle size distributions shown in the insets.


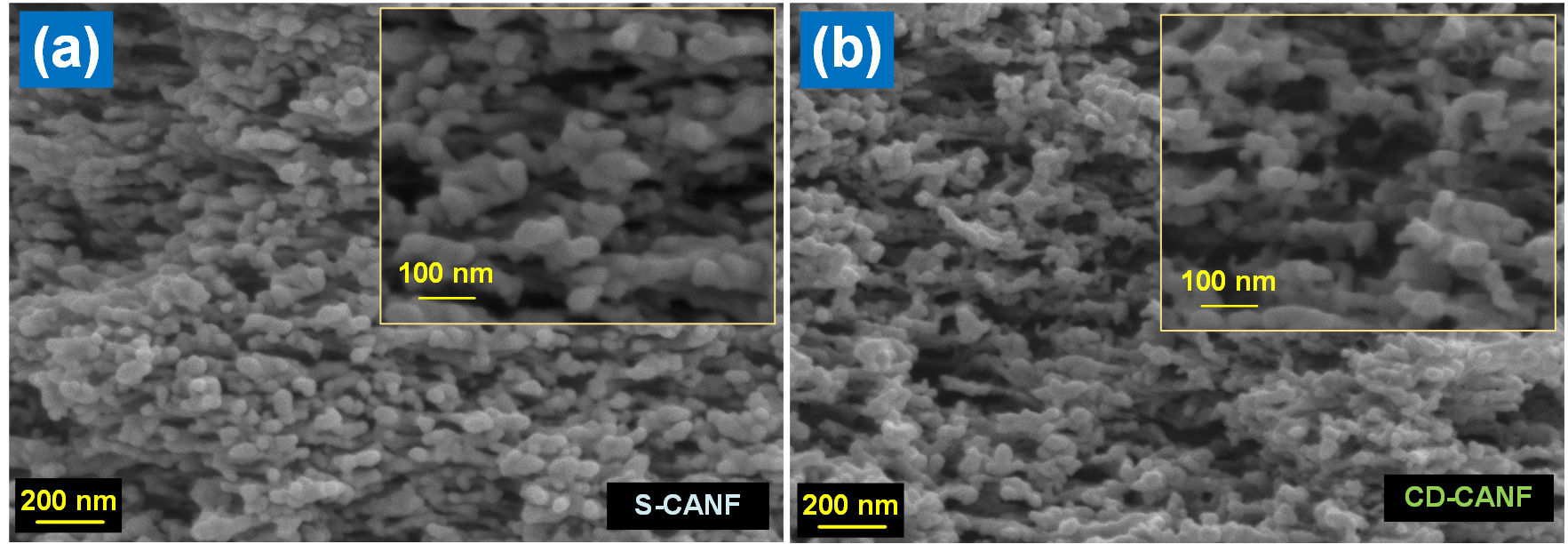


**Figure S9.** Cross-sectional SEM images of carbonized interlayers:

(a) S-CANF (2 h) and (b) CD-CANF (2 h).


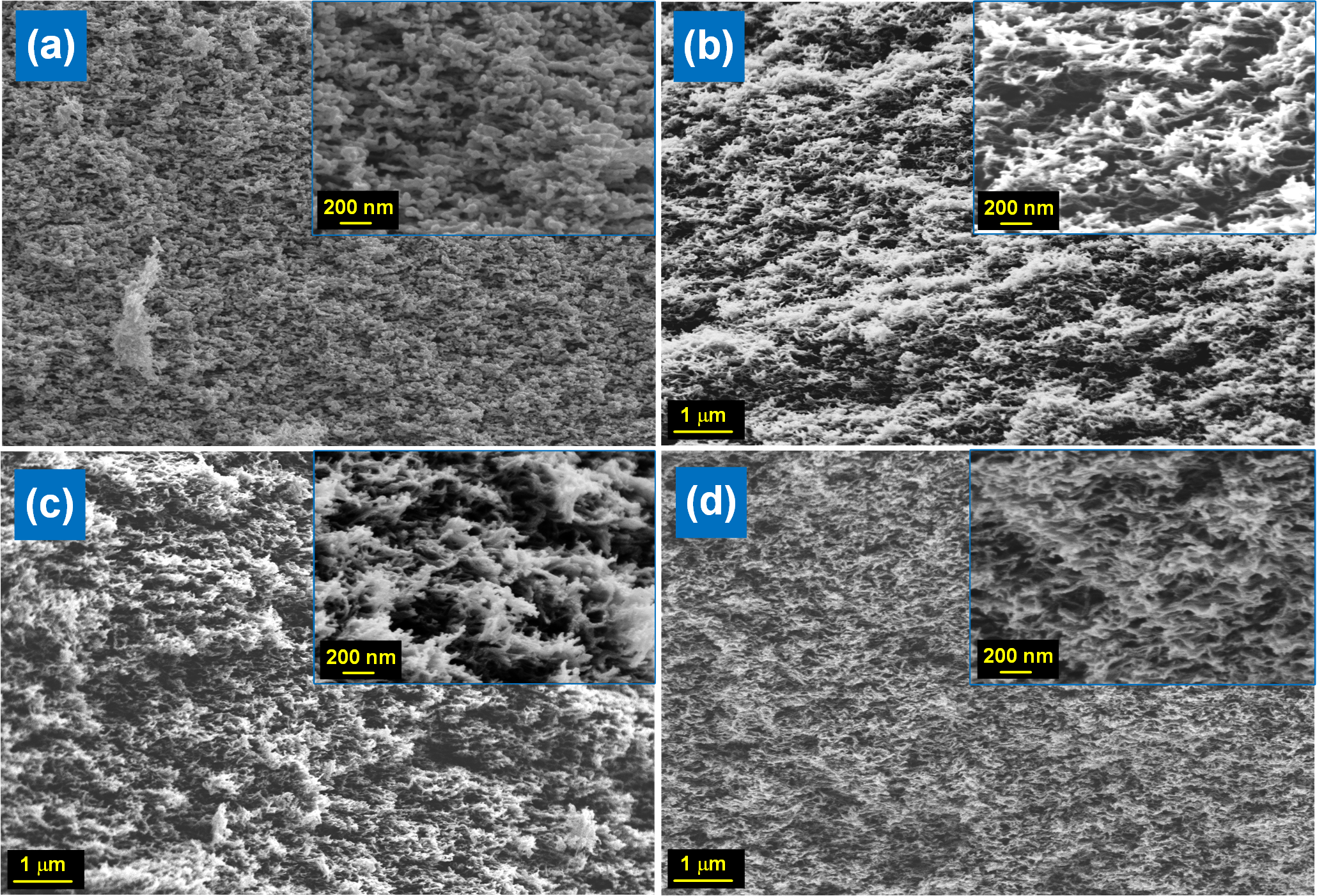


**Figure S10**. Cross-sectional SEM images of carbonized interlayers:

time-dependent structural evolution of CD-CANF after carbonization with growth durations of (a) 3 h, (b) 6 h, (c) 12 h, and (d) 16 h.


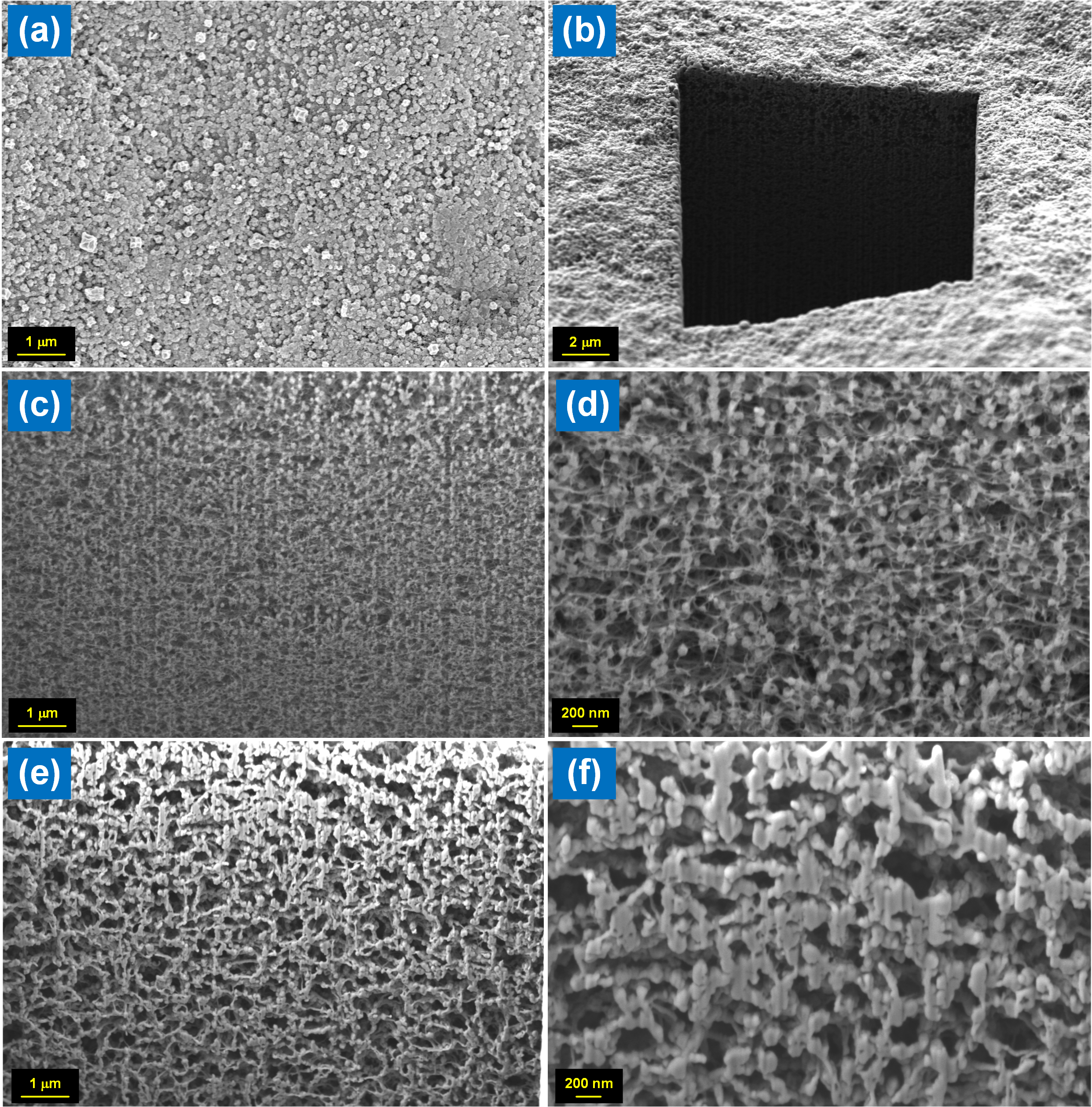


**Figure S11.** FIB characterization of the carbonized S-CANF interlayer. (a) Top-view SEM image of the surface corresponding to the dense catalytic layer. (b) FIB-milled cross-sectional trench of the interlayer. (c, d) Cross-sectional SEM images of the dense surface layer. (e, f) Cross-sectional SEM images of the underlying porous fibrous layer.


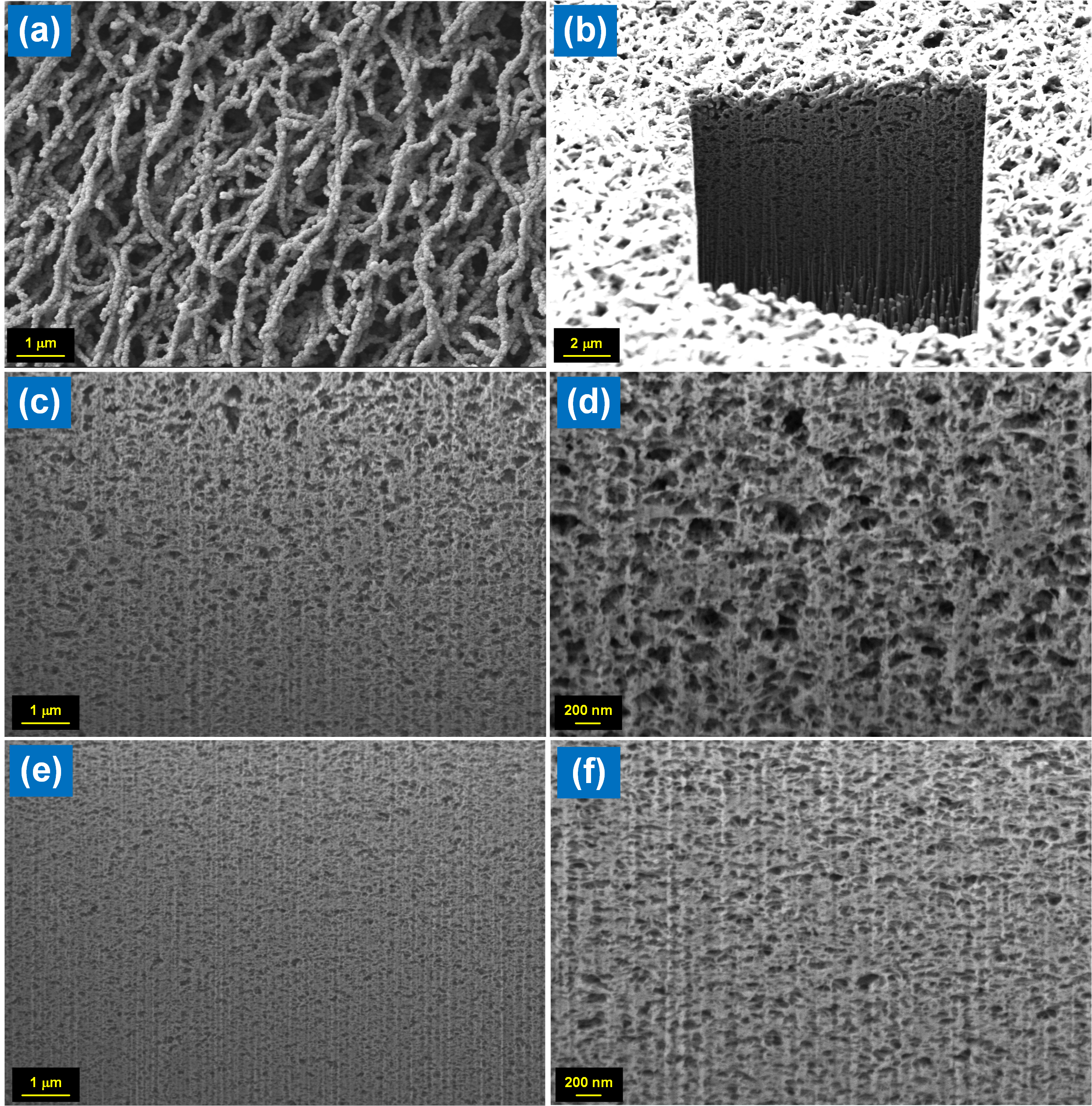


**Figure S12.** FIB characterization of the carbonized CD-CANF interlayer. (a) Top-view SEM image of the surface corresponding to the dense catalytic layer. (b) FIB-milled cross-sectional trench of the interlayer. (c, d) Cross-sectional SEM images of the dense surface layer. (e, f) Cross-sectional SEM images of the underlying porous fibrous layer.


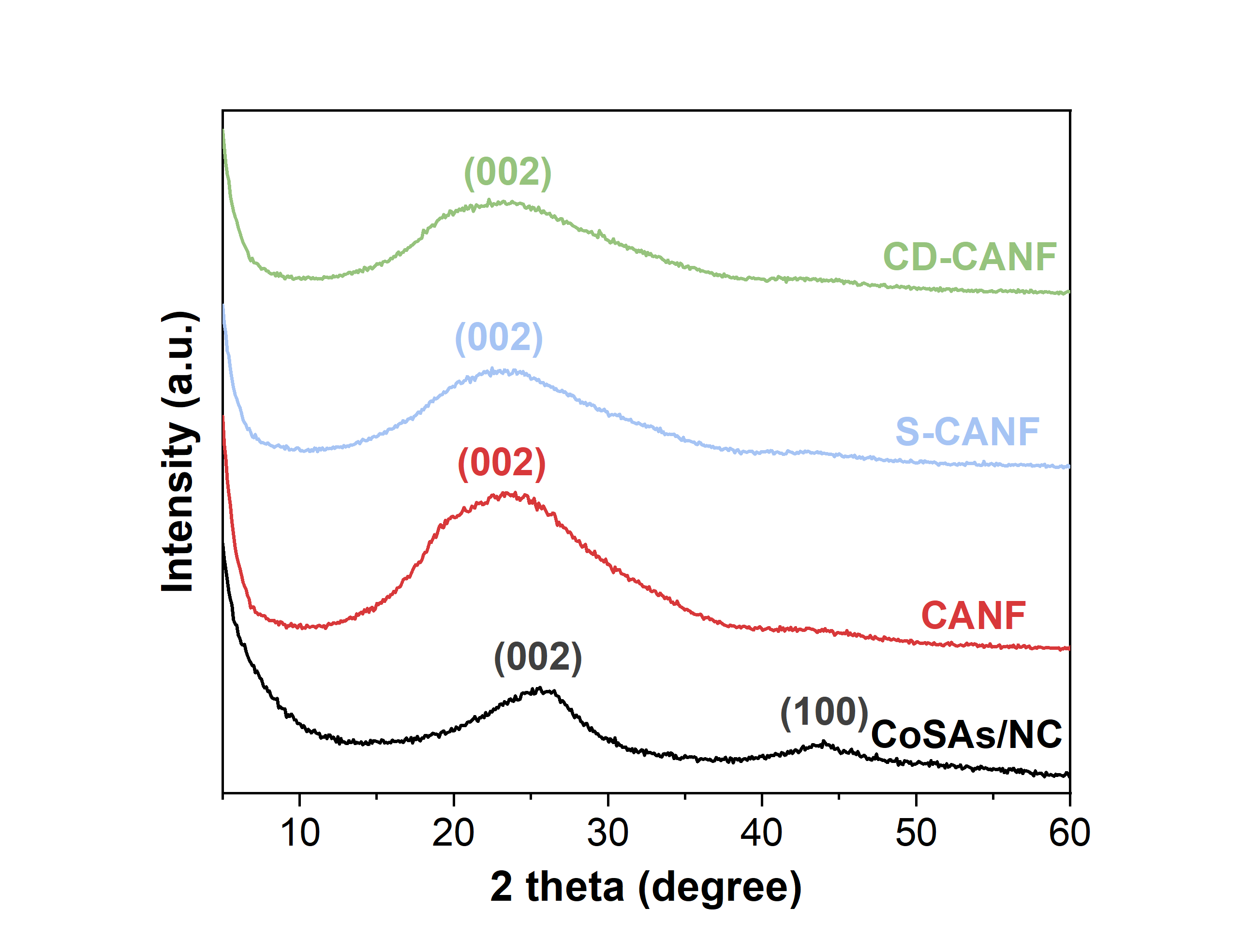


**Figure S13.** XRD patterns of CANF, S-CANF, CD-CANF, and CoSAs/NC after pyrolysis.


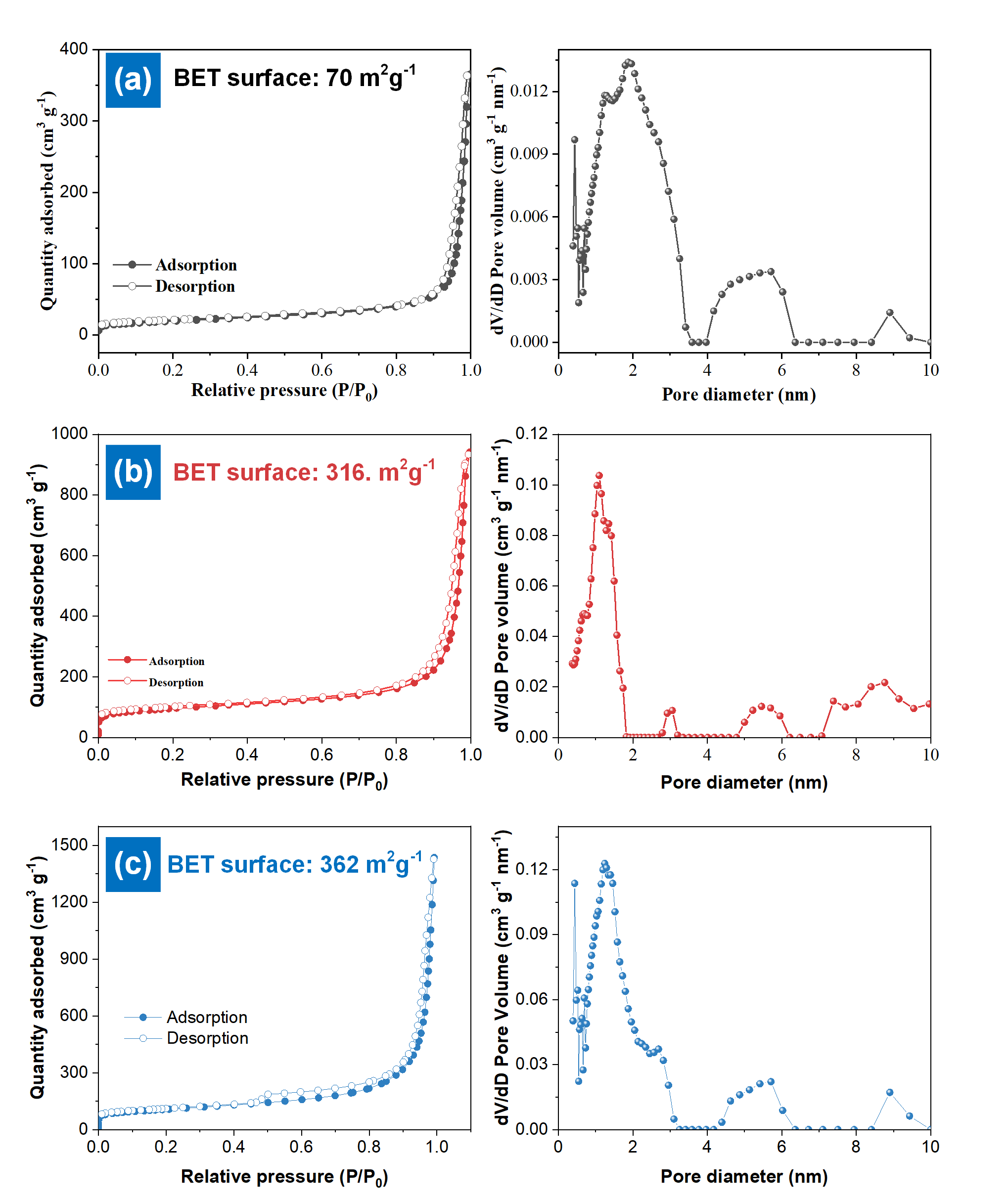


**Figure S14.** N₂ adsorption-desorption isotherms (left) and corresponding pore size distributions (right) of (a) CoSAs/NC, (b) S-CANF, and (c) CD-CANF. The BET surface areas are 70, 316, and 362 m^2^ g^-1^, respectively.


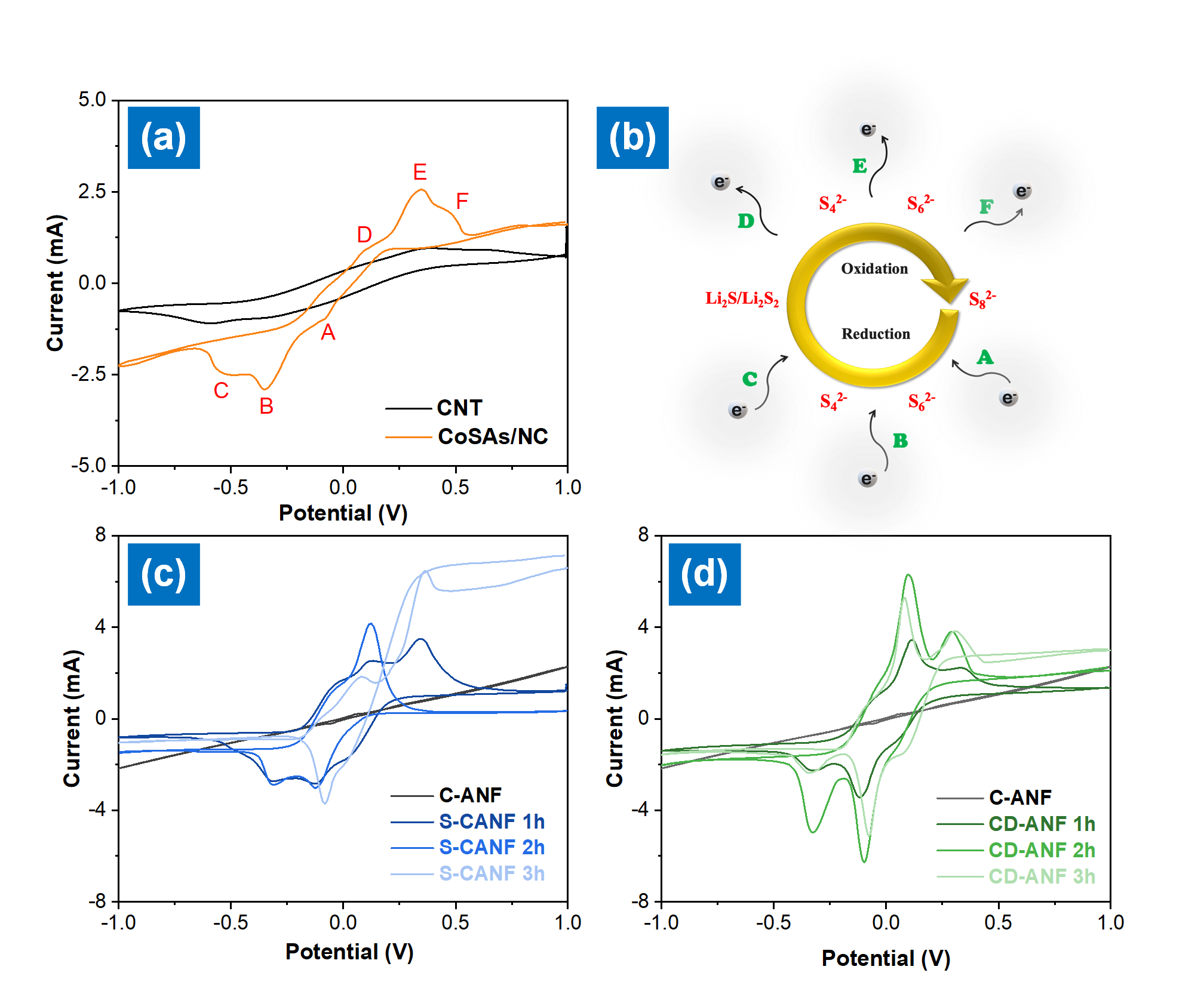


**Figure S15.** CV analysis of (a) CNT and CoSAs/NC catalysts with Li₂S₆, (b) corresponding LiPS conversion scheme, and growth-time-dependent catalytic activity of (c) S-CANF and (d) CD-CANF interlayers.


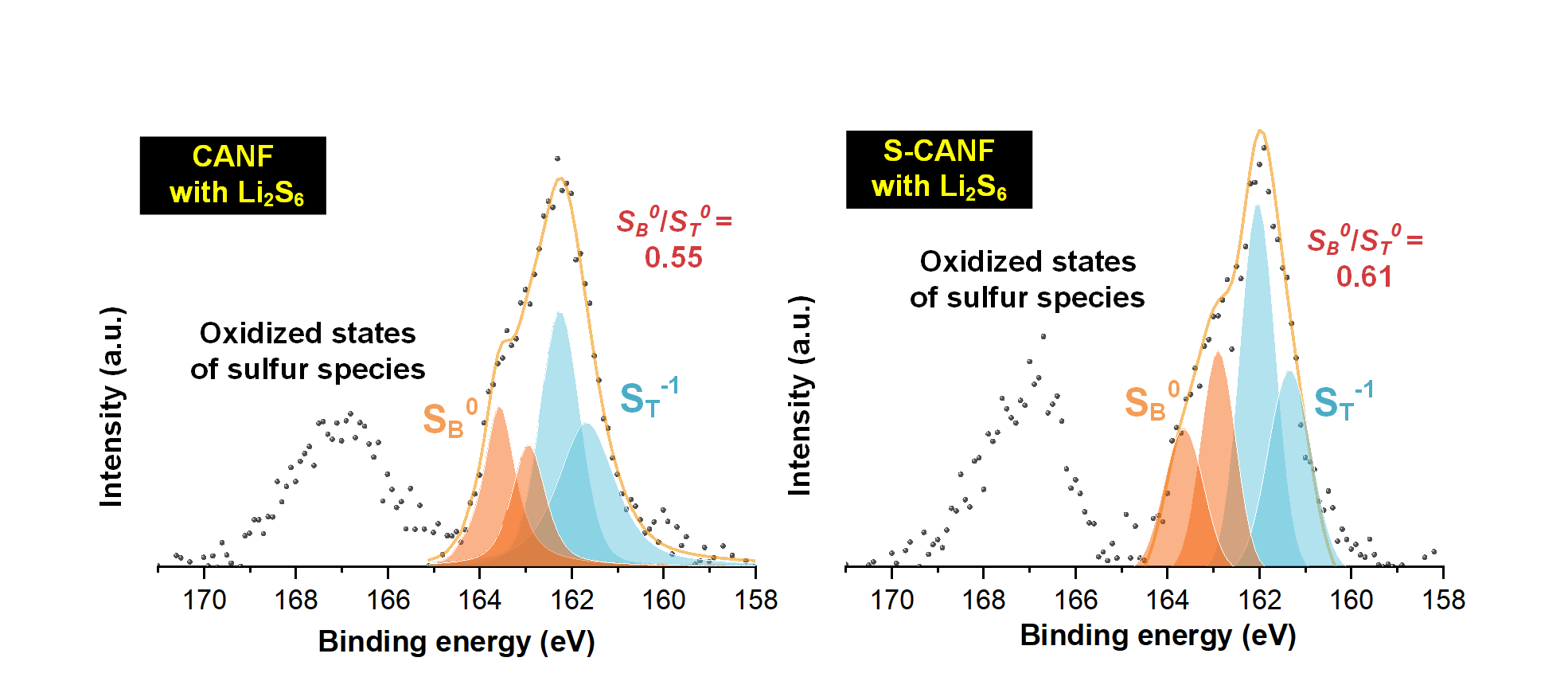


**Figure S16.** S 2p XPS spectra of CANF and S-CANF after Li₂S₆ exposure. Deconvoluted terminal (*S_T_^-1^*) and bridging (*S_B_^0^*) sulfur species are shown, with *S_B_^0^*/*S_T_^-1^* ratios of 0.55 (CANF) and 0.61 (S-CANF).


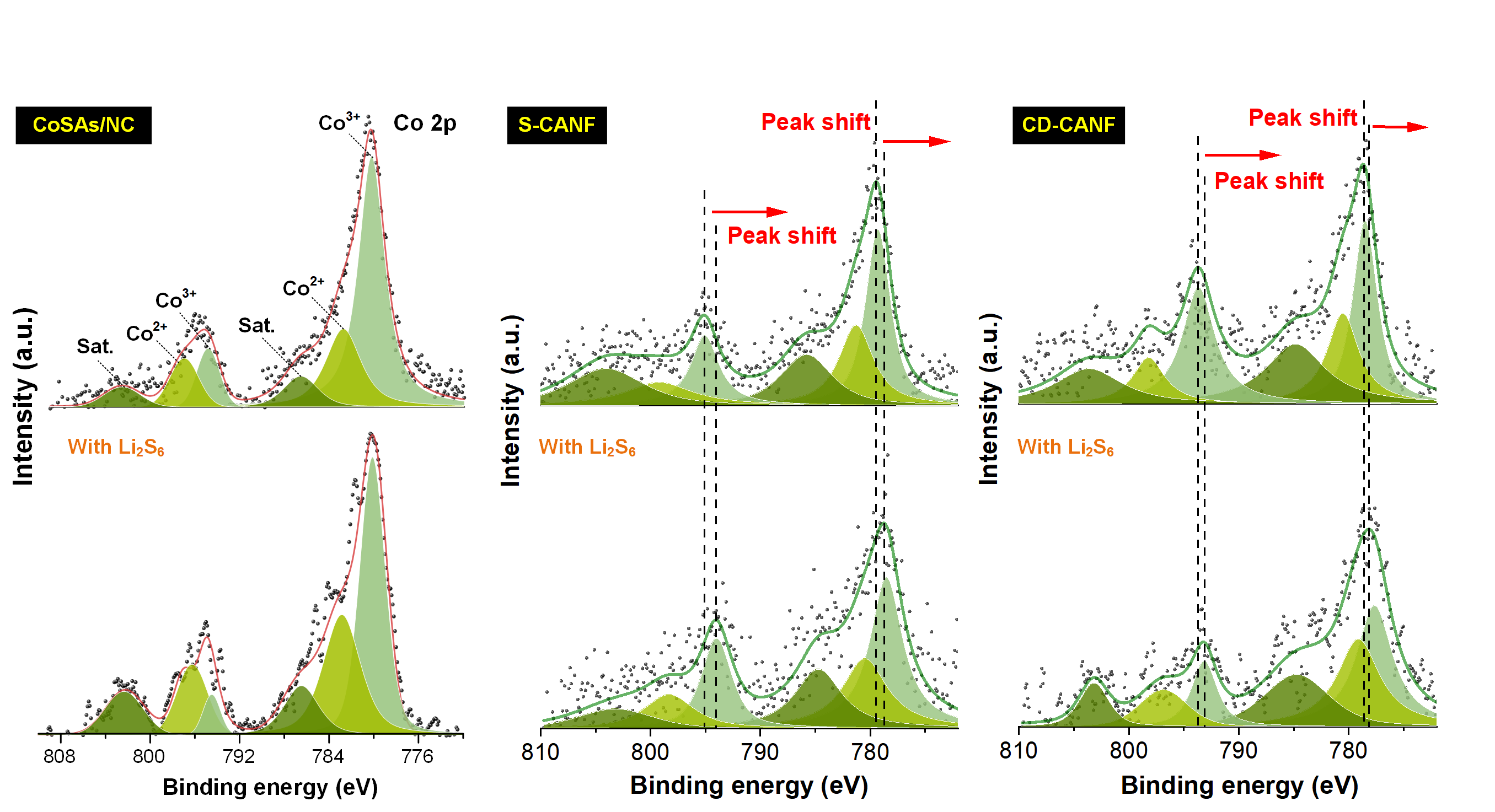


**Figure S17**. Co 2p XPS spectra of CoSAs/NC, S-CANF, and CD-CANF before and after Li₂S₆ adsorption.


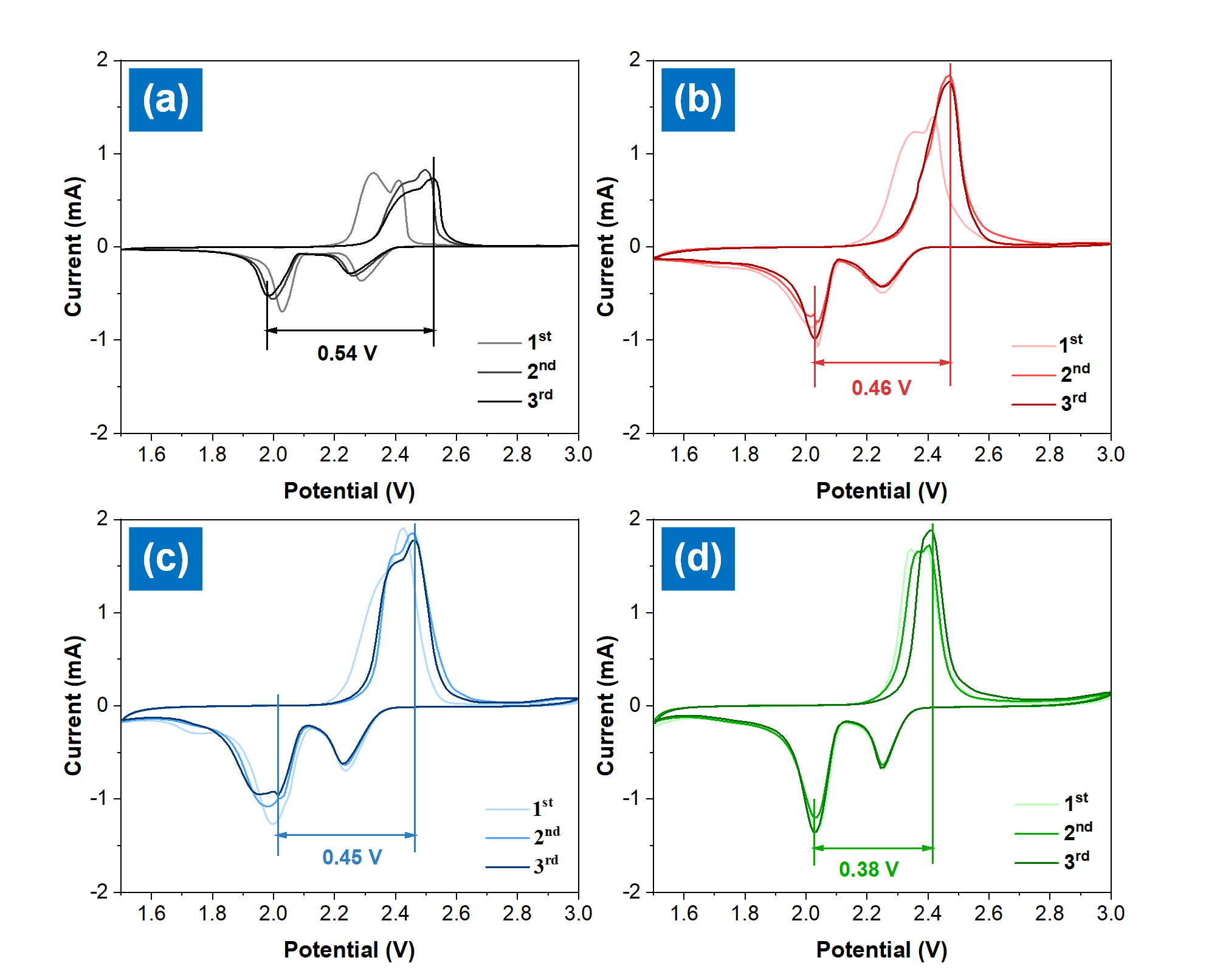


**Figure S18**. CV curves symmetric cells with pristine PE, CANF, S-CANF, and CD-CANF interlayers, with indicated peak separations used for comparison of redox kinetics.


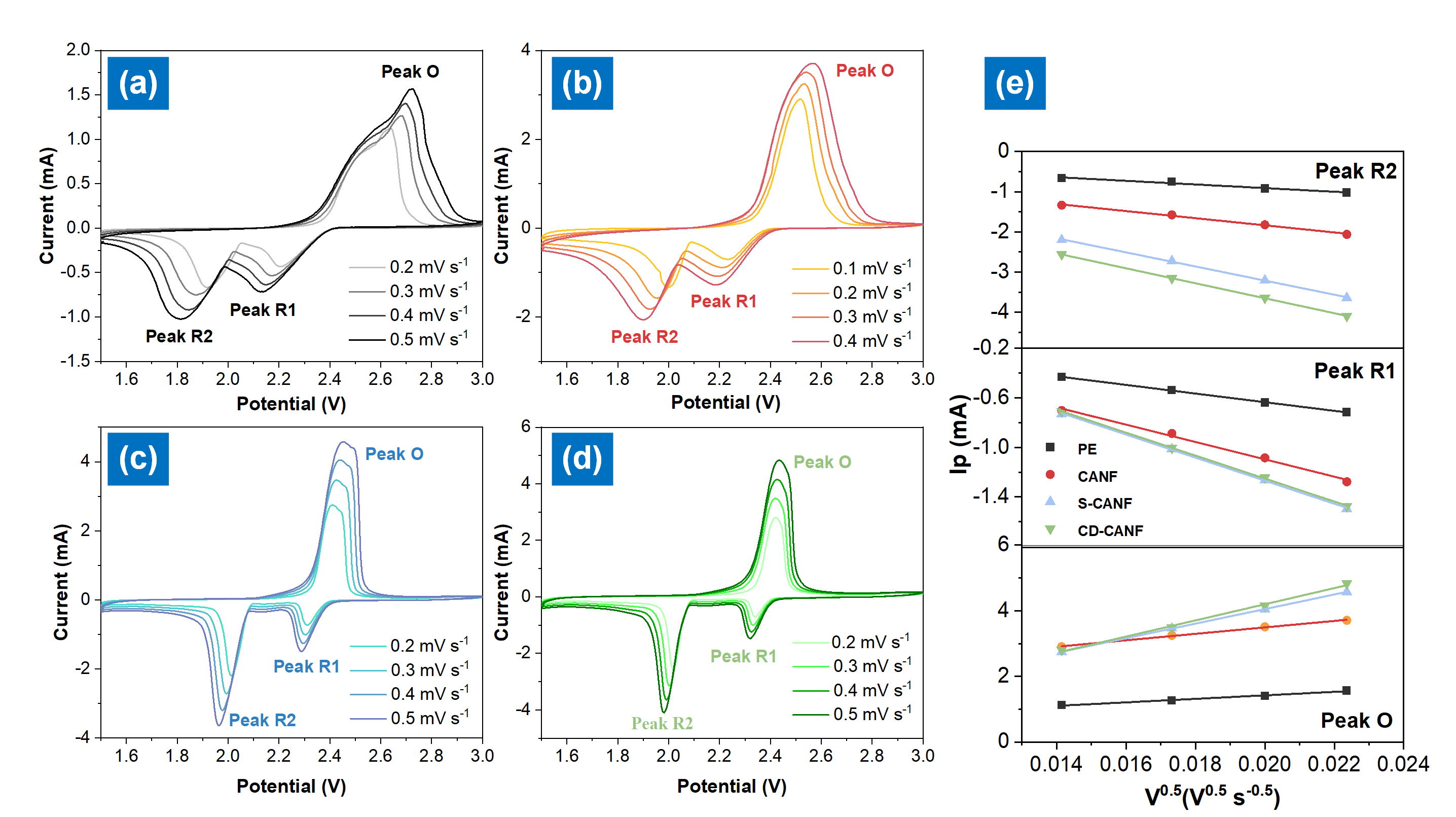


**Figure S19**. CV curves of symmetric cells assembled with (a) pristine PE, (b) CANF, (c) S-CANF, and (d) CD-CANF interlayers at a scan rate of 0.1 mV s^-1^. The peak separations (*ΔE*) between the anodic (O) and cathodic (R1, R2) redox transitions are indicated for comparison of LiPS conversion kinetics and interfacial polarization.


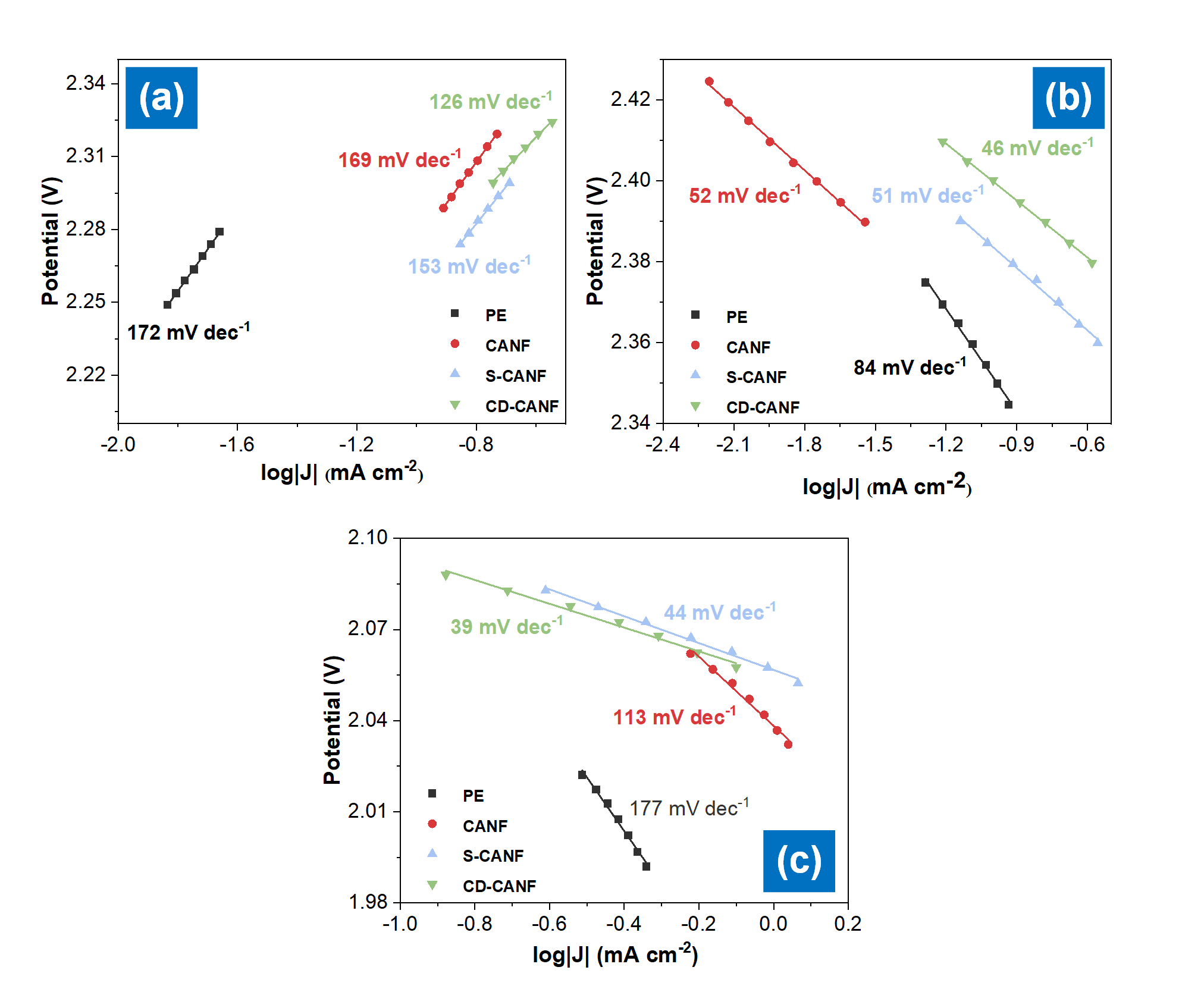


**Figure S20**. Tafel plots derived from the CV curves of symmetric cells for (a) Peak O, (b) Peak R1, and (c) Peak R2, comparing pristine PE, CANF, S-CANF, and CD-CANF interlayers.

**
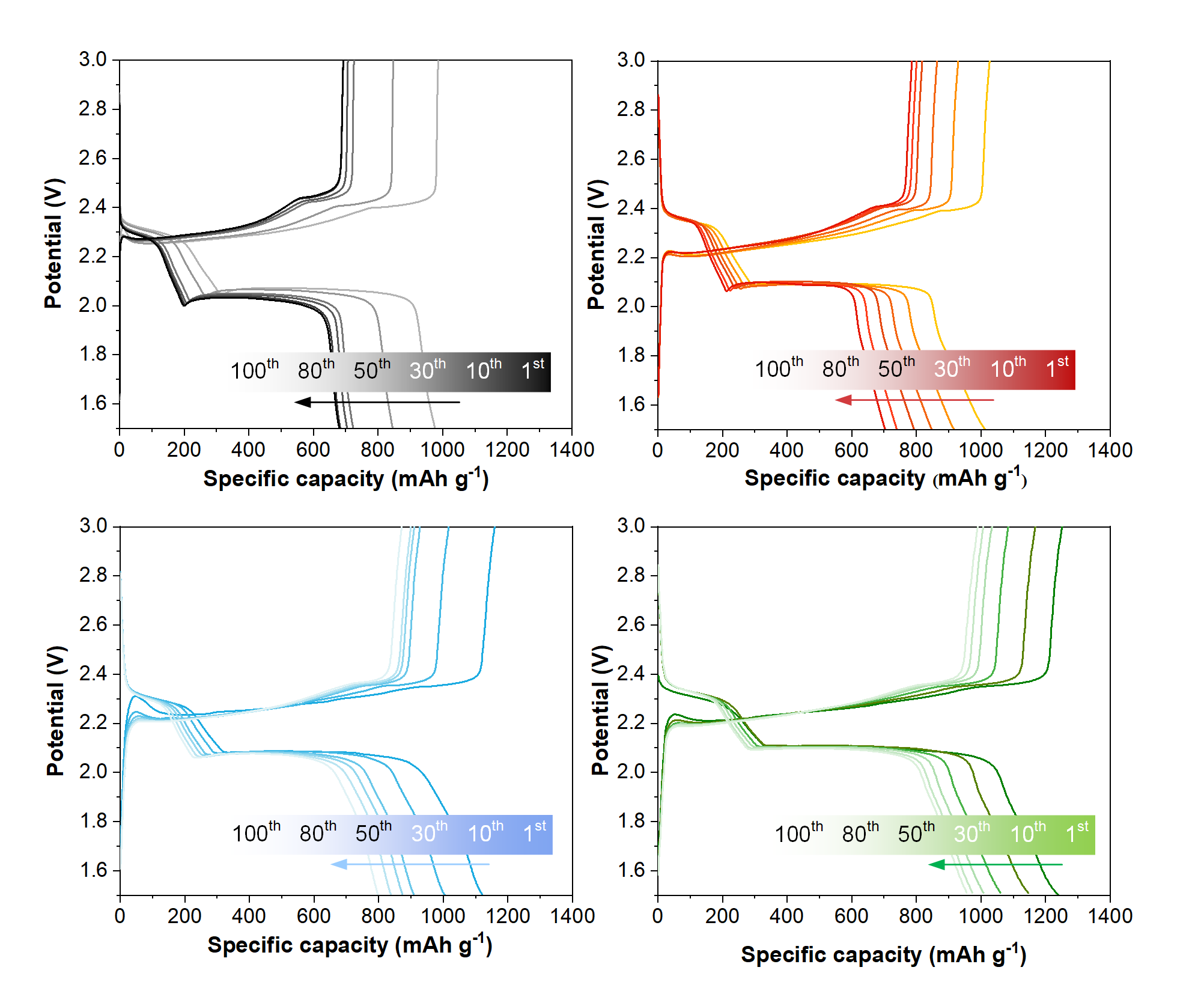
**

**Figure S21**. Charge-discharge voltage profiles of Li-S cells using (a) PE, (b) CANF, (c) S-CANF, and (d) CD-CANF interlayers at selected cycle numbers (1^st^, 10^th^, 30^th^, 50^th^, 80^th^, and 100^th^).


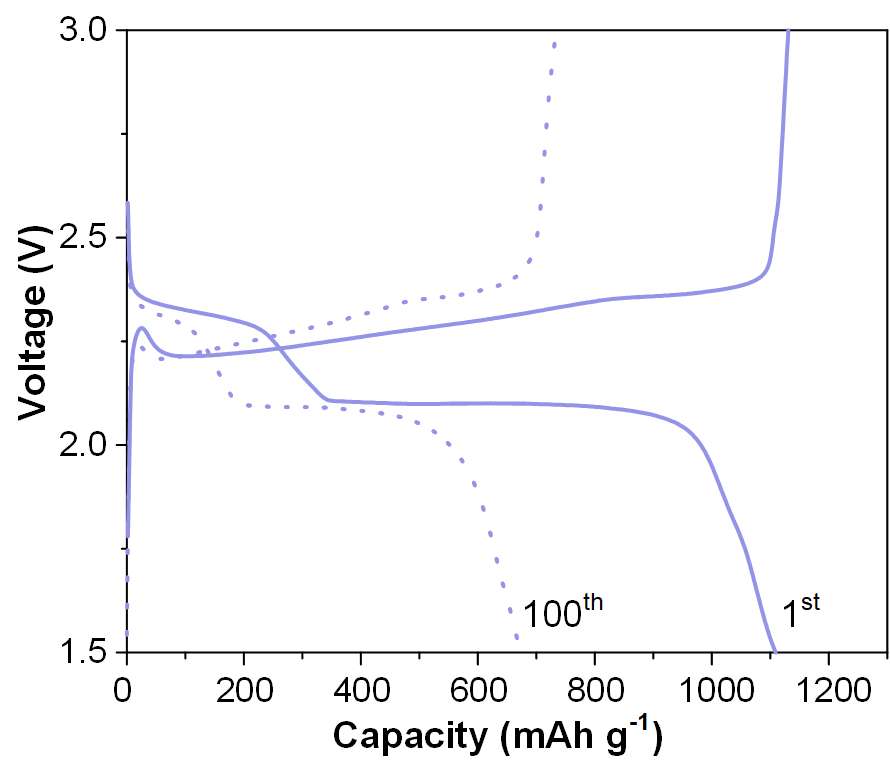


**Figure S22.** Charge-discharge voltage profiles of the cells with CoSAs/NC@PE modified separator.


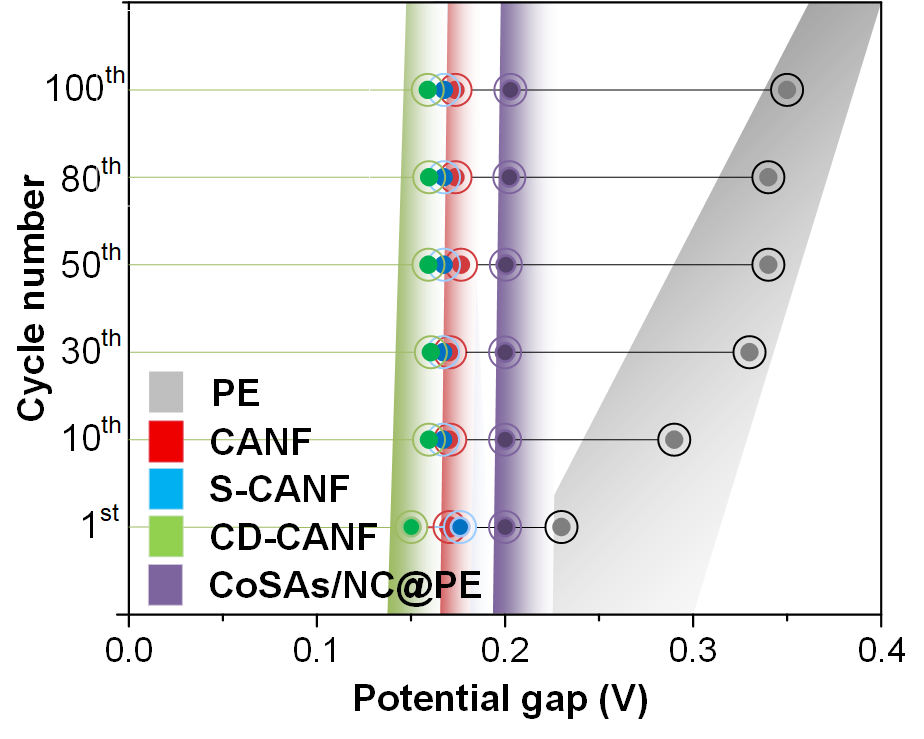


**Figure S23.** Potential gap of cells using PE, CANF, S-CANF, CD-CANF, and CoSAs/NC@PE at 50% discharge capacity.


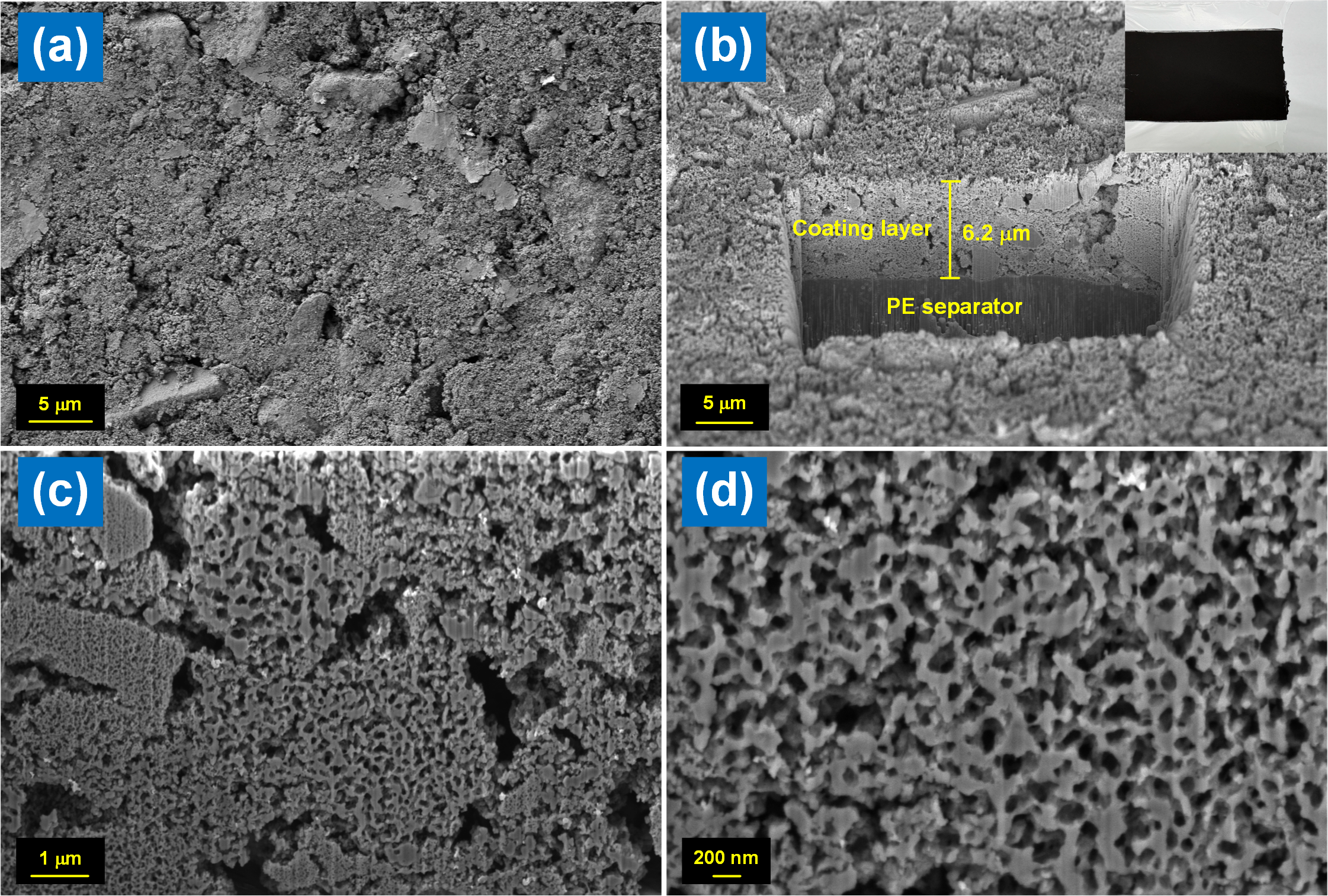


**Figure S24.** FIB characterization of the CoSAs/NC@PE modified separator. (a) Top-view SEM image of the surface corresponding to the CoSAs/NC coating layer. (b) FIB-milled cross-sectional trench of the modified separator. (c, d) Cross-sectional SEM images of the coating layer.


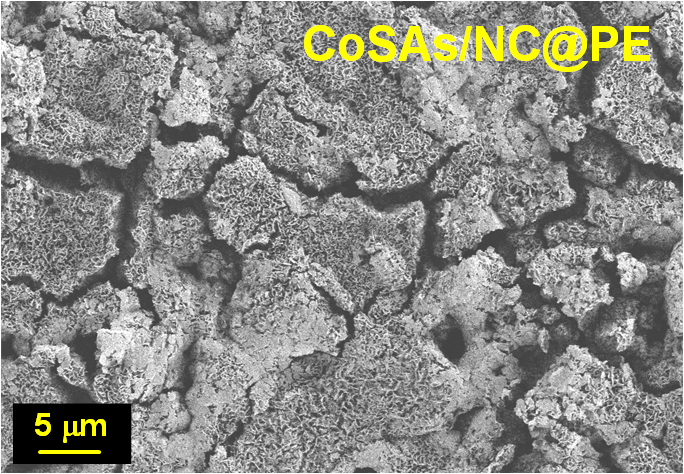


**Figure S25.** SEM images of Li anode after cycling with CoSAs/NC@PE modified separator.


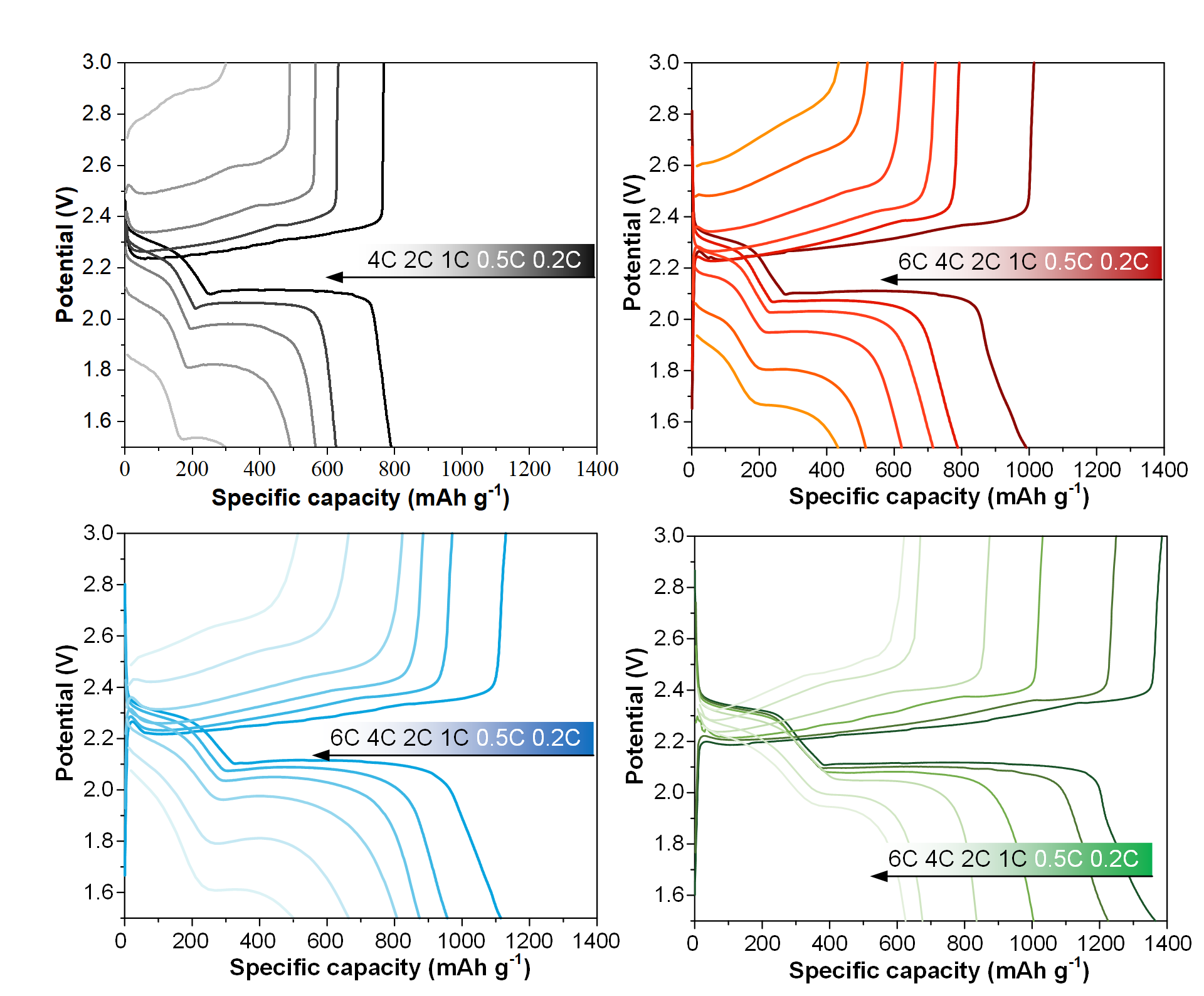


**Figure S26.** Galvanostatic charge-discharge curves of Li-S cells with (a) PE, (b) CANF, (c) S-CANF, and (d) CD-CANF interlayers measured at 0.2, 0.5, 1, 2, 4, and 6 C.


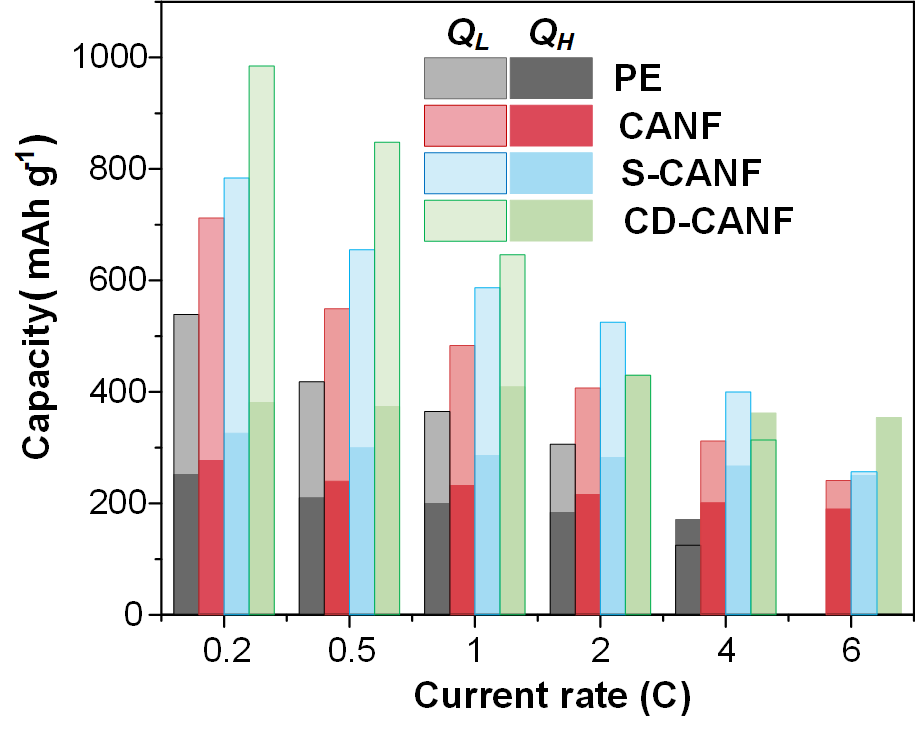


**Figure S27.** Discharge capacities of cells using PE, CANF, S-CANF, and CD-CANF interlayers corresponding to the low-voltage plateau (*Q_L_*) and high-voltage plateau (*Q_H_*) at various C-rates.


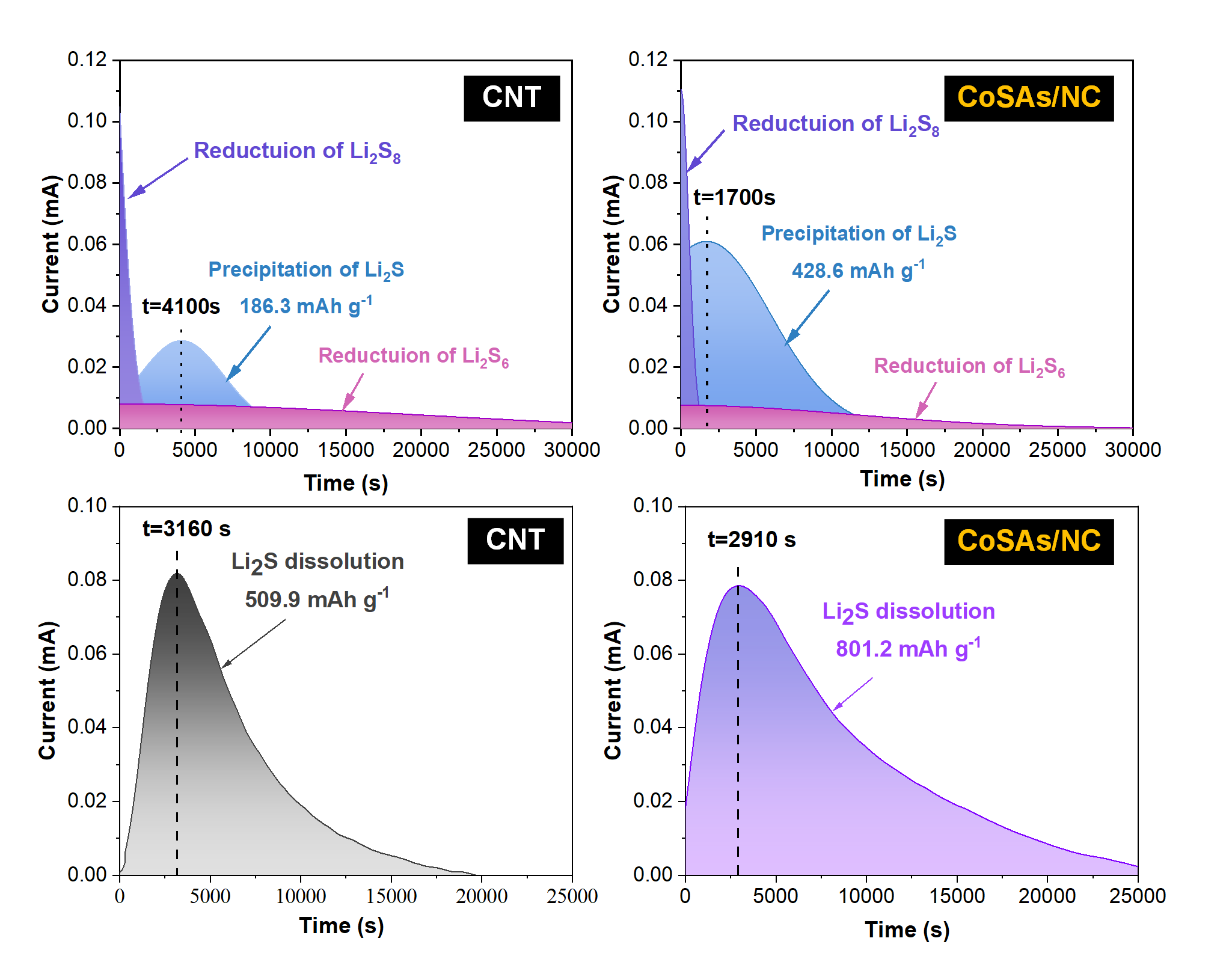


**Figure S28**. Potentiostatic nucleation and dissolution behaviors of Li₂S on CNT and CoSAs/NC electrodes.


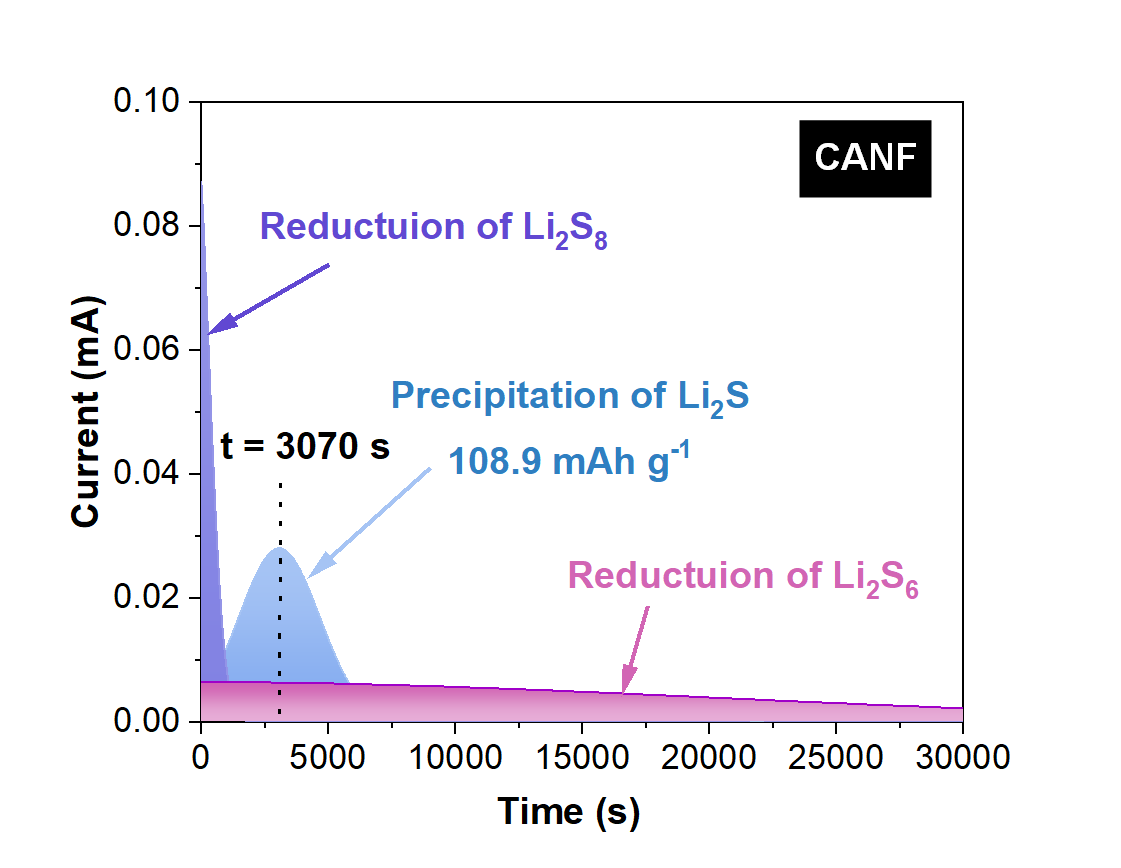


**Figure S29.** Potentiostatic Li₂S precipitation profiles of CANF, highlighting the induction time and Li₂S precipitation capacity.


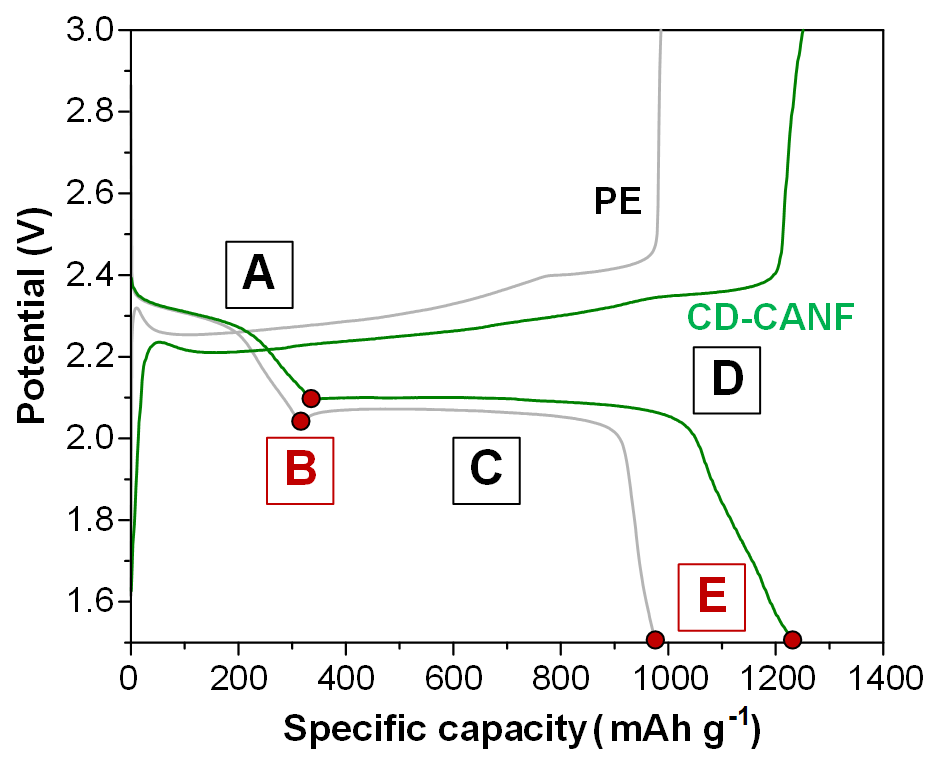


**Figure S30.** Points B and E correspond to the intermediate discharge plateau and deep discharge state used for ex situ XPS analysis in **Figure 7b**.


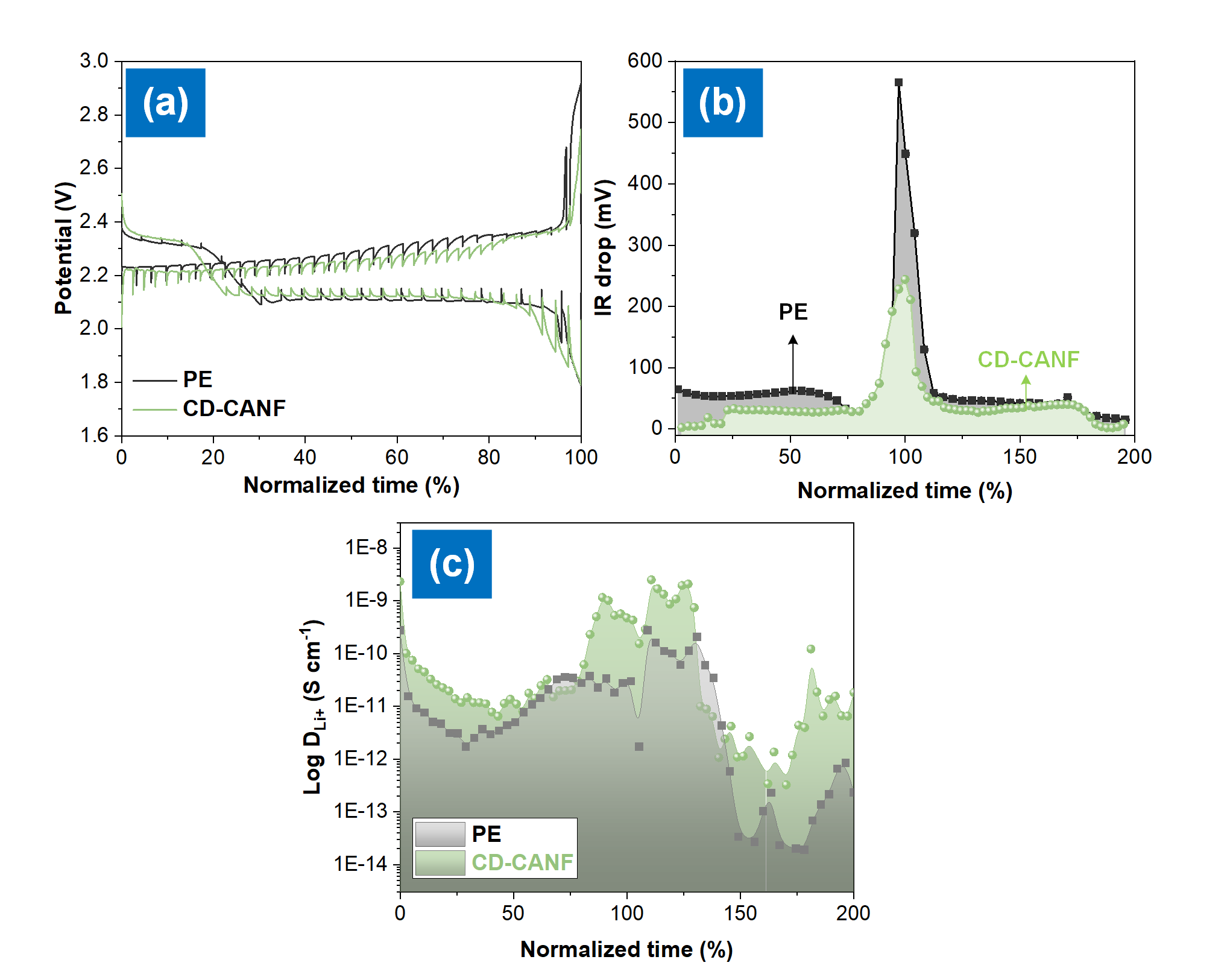


**Figure S31.** (a) Normalized galvanostatic voltage profiles comparing PE and CD-CANF. Comparison of GITT-derived (b) IR drop profiles and (c) Li⁺ diffusion coefficients of Li-S cells with pristine PE and CD-CANF interlayers plotted as a function of normalized time.

**Table S1**. Comparison of reported interlayer and separator modification strategies for long-term cycling stability in Li-S batteries.

| System / Interlayer | Electrode / Interlayer Composition | Rate & Cycle Number | Decay Rate/  per cycle (%) | References |
| --- | --- | --- | --- | --- |
| NC@SA-Co/ CNT-CNF Freestanding Interlayer | Freestanding carbon nanofiber membrane with atomically dispersed Co SAC | ~0.1 C initial; long cycling at 700 cycles | 0.058 | [7] |
| Fe/Zn Single-Atom on Porous CNF  (FeZn-PCNF) | Electrospun CNF membrane implanted with Fe/Zn SAC | 1 C, 600 cycles | 0.06 | [8] |
| Co₉S_8-x_/CNT Interlayer | Sulfur-deficient Co₉S₈ nanosheets on CNT conductive framework | 0.3 C, 1000 cycles | 0.049 | [9] |
| P-PMIA@ZIF-8 Gel Nanofiber Separator | PMIA nanofiber membrane with in-situ grown ZIF-8 + PVDF-HFP | 0.2 C, 300 cycles | 0.086 | [10] |
| Z-PMIA  (ZIF-L(Co) on Aramid Nanofiber) | ANF separator coated with 2D ZIF-L(Co) nanosheets | 0.2 C, 350 cycles | 0.033 | [11] |
| TiO₂-C65 Coated Separator | Thin multifunctional TiO₂/C65 coating | 0.5 C, 500 cycles | 0.1 | [12] |
| Porous Carbon Sheet + TiO₂ Nanoparticles Interlayer | TiO₂ NP embedded porous carbon interlayer | 0.5 C, 300 cycles | 0.063 | [13] |
| SnO₂@MXene laminar interlayer | Ultrathin SnO₂ QDs/MXene heterostructure coated on PP separator | 2 C, 500 cycles | 0.052 | [14] |
| N-M@CNi MXene separator | N-doped MXene–carbon nanosheet–Ni composite coated PP separator (N-M@CNi) | 0.2 C, 500 cycles | 0.043 | [15] |
| MXene-Nafion laminar separator | Laminar MXene-Nafion composite layer on PP separator | 1 C, 1000 cycles | ≈0.03 | [16] |
| Pore-space-partitioned MOF separator | Pore-space-partitioned MOF layer（PSP-MOF）coating on PVDF-HFP/PP | 0.5 C, 500 cycles | ≈0.042 | [17] |
| VO₂@rGO-modified separator | VO₂@rGO Mott–Schottky catalyst coating on PP separator | 2 C, 600 cycles | 0.048 | [18] |
| PVDF-C coated separator | PVDF-C coating on commercial separator（S + Super P mixture cathode） | 0.5 C, 500 cycles | ≈0.068 | [18] |
| MoS₂@CF-NrGO | Hierarchical MoS₂ nanosheets anchored on nitrogen-doped rGO-reinforced carbon fibers | 1 C, 1000 cycles | 0.064 | [19] |
| Nb₂O₅/rGO | Polar Nb₂O₅ quantum dots distributed on conductive rGO nanosheets | 0.3 C, 500 cycles | 0.086 | [20] |
| CeO₂@Graphene | CeO₂ nanoparticles anchored onto graphene sheets forming a polar catalytic interlayer | 0.5 C, 200 cycles | 0.12 | [21] |
| SrF₂/Graphene | SrF₂ nanoparticles grown on graphene creating polar-conductive hybrid interlayer | 0.5 C, 350 cycles | 0.05 | [22] |
| Co-3DC-rGO | 3D cobalt-carbon framework integrated with rGO nanosheets | 1 C, 500 cycles | 0.12 | [23] |
| Ni₃Sn₂/NG | Nickel-tin alloy nanoparticles uniformly distributed over nitrogen-doped graphene | 1 C, 400 cycles | 0.07 | [24] |
| rGO@MoS₂ | MoS₂ nanolayers anchored on rGO sheets forming a conductive catalytic interlayer | 1 C, 500 cycles | 0.116 | [25] |
| CoPc@GO | Molecular Co-phthalocyanine immobilized on GO nanosheets acting as a molecular-level catalyst | 1 C, 400 cycles | 0.08 | [26] |
| Sb₂Se₃/rGO | Antimony selenide nanorods anchored on rGO conductive sheets | 1 C, 500 cycles | 0.03 | [27] |
| Ni₃B@rGO | Nickel boride nanoparticles homogeneously dispersed on rGO | 2 C, 500 cycles | 0.06 | [28] |
| Ni@C/Graphene | Core-shell Ni@C nanoparticles supported on graphene network | 0.5 C, 1000 cycles | 0.061 | [29] |
| CoSAs/CD-CANF | Contra-diffusion grown ZIF precursor forming uniformly dispersed Co-Nₓ single-atom catalytic sites embedded in carbonized ANF nanofiber membrane | 2 C, 2000 cycles | 0.03 | This work |

**Table S2.** Summary of representative SAC systems for Li-S batteries categorized by catalyst configuration, including cathode-host, separator-modified, and interlayer-based architectures. Metal loading, cycling conditions, and capacity decay rates are summarized to enable cross-comparison of catalytic efficiency.

| Code | System / SAC metal | Composition & role | Host / Interlayer type | Metal loading  (wt%) | Current density & cycles  (long-term) | Capacity decay rate  (% per cycle) | Ref |
| --- | --- | --- | --- | --- | --- | --- | --- |
| (A) SAC Cathode-Host Systems | | | | | | | |
| 1 | CoSA-NB (CoN₃B) | CoN₃B SAs anchored on porous CNF; B-coordination enhances LiPS anchoring + SRR kinetics | Self-supporting electrospun CNF host | - | 1 C, 500 cycles  2 C, 1800 cycles | 0.076 (1C)  0.045 (2C) | [30] |
| 2 | Co SAs on CN nanosheets (CC@CN-SACo) | Co SAs anchored on carbon nanosheets; enhances Li adsorption + LiPS conversion | Carbon nanosheet host | - | 0.2 C, 300 cycles (S/CC@CN-SACo) | ≈ 0.034 | [31] |
| 3 | Fe-N₄ (FeANAC/OC) | Fe SAs anchored on hierarchically ordered porous carbon; accelerates LiPS conversion and improves rate kinetics | SAC porous carbon host | - | 1 C, 300 cycles | 0.036 | [32] |
| 4 | V-N-C  (V SAs + VN NPs) | Isolated V SAs (V-N₄ type) + ultra-small VN nanoparticles embedded in N-doped carbon; dual active centers synergistically accelerate LiPS redox | SAC sulfur host（cathode host） | 0.7 at% V  （XPS） | 2 C, 1000 cycles | 0.052 | [33] |
| 5 | Co-SAs@NC | Co SAs anchored on nitrogen-doped carbon polyhedra; Co-N₄ SAC sites accelerate LiPS conversion and stabilize sulfur redox | SAC sulfur host（MOF-derived N-doped carbon） | 0.66 wt% Co（ICP-OES） | 1 C, 600 cycles | ≈ 0.023 | [34] |
| 6 | CoSA-N-C  （Co-N₄ SAC） | High-loading Co SAs (Co-N₄) anchored on 2D N-doped carbon nanosheets；accelerates LiPS redox, suppresses shuttle, regulates Li₂S deposition morphologies | SAC sulfur host（cathode host） | ≈ 15.3 wt% Co | 1 C, 1000 cycles | 0.035 | [35] |
| 7 | Co-O Axially Coordinated SAs | Co SAs with axial Co-O/N coordination hosted in conductive MOF nanosheets (Co-HTP) anchored on carboxylated graphene; modulates LiPS redox kinetics by tuning electronic structure | MOF-derived SAC sulfur host  (Co-HTP/CG composite) | - | 1 C, 500 cycles | 0.052 | [36] |
| 8 | Co-N_x_ SAs（Co SAs） | Microporous carbon with uniformly dispersed Co-N_x_ SAs; solid-solid sulfur conversion + lowering activation energy + suppressing LiPS formation | SAC Microporous Carbon Host（Cathode host） | ≈ 2 wt% Co（ICP-MS） | 0.5 C, 1000 cycles | 0.016 | [37] |
| 9 | Co-N/P-S Asymmetric SAs | Asymmetric P/S/N coordination around isolated Co atoms (Co-N₃PS); *d-p* orbital hybridization enhances catalytic activity + accelerates LiPS redox kinetics | SAC heteroatom-rich hollow carbon nanocage host | 0.29 at% Co (XPS)  0.10 at% Co (EDS) | 1 C, 1000 cycles  5 C, 2000 cycles | 0.038 (1C)  0.027 (5C) | [38] |
| 10 | FeSA-CN  (Fe SAs) | Fe SAs anchored on nitrogen-rich MOF-derived carbon nanocage; catalyzes LiPS conversion + enhances adsorption | SAC porous carbon host (cathode host) | 1.14 wt% Fe (ICP-OES) | 4 C, 500 cycles | 0.06 | [39] |
| 11 | Nb-SAs@NC (Nb SAC) | Isolated Nb SAs coordinated as distorted Nb-N₄ sites; strong trapping-coupling-conversion catalytic mechanism for LiPS anchoring and conversion | SAC nitrogen-doped carbon (MOF-derived) sulfur host | 1.42 wt% Nb (ICP-OES) | 4 C, 1000 cycles | 0.015 | [40] |
| 12 | FeSA-PCNF (Fe SAs) | Fe SAs coordinated in N-doped porous carbon nanofibers; strong LiPS adsorption + fast electrocatalysis | Self-standing sulfur cathode (host) | 0.78 wt% Fe | 2C, 500 cycles | 0.048 | [41] |
| 13 | Ni-N₅/HNPC | Isolated Ni SAs (Ni-N₅ coordination) embedded in hollow N-doped porous carbon capsules; catalyzes LiPS redox + enhances conductivity + confines LiPSs | SAC hollow porous carbon host | ≈ 0.6 wt% Ni (ICP-OES) | 0.5 C, 500 cycles | 0.053 | [42] |
| 14 | SACo/NDC (Co SAs on N-doped 3D carbon) | Freeze-dried salt-templated 3D N-doped carbon with atomically dispersed Co-N_x_ sites; strong LiPS adsorption + catalytic redox promotion | SAC sulfur host | 3.18 wt% Co（ICP-OES） | 0.5 C, 300 cycles | 0.08 | [43] |
| (B) SAC Separator-Modified Systems | | | | | | | |
| 15 | CoSA (ALD) | Atomically dispersed Co deposited by atomic layer deposition; catalytic LiPS conversion | ALD-modified PP separator | ~0.6 wt% Co | 1 C, 500 cycles | ≈0.045 | [44] |
| 16 | CoSAC-NC (Co-Nₓ sites) | Co SAs anchored on ordered macro-microporous N-doped carbon; catalyzes LiPS conversion + enhances Li⁺ transport | SAC separator coating (MOF-derived ordered macro-microporous carbon) | *-* | 1 C, 1000 cycles | 0.043 | [45] |
| 17 | Axial Co-O SAs  (MOF-Co-O) | Axially coordinated Co-O SAs in MOF network; accelerate LiPS redox | SAC MOF-modified separator | - | 0.5 C, 500-700 cycles | ≈0.05 | [46] |
| 18 | Fe-N₅/GCNC (Fe SAs) | Asymmetric Fe-N₅ SA centers supported on graphitic carbon nanocapsules; catalyzes LiPS conversion + reinforces adsorption | SAC-modified separator  (Fe-N₅/GCNC coating on PP separator) | 0.49 wt% Fe (ICP-OES);  0.41 at% (XPS) | 1 C, 1000 cycles | 0.0386 | [47] |
| 19 | Co-O₂N₂ SAC (Co/NOC) | Co SAs with O₂N₂ coordination on N,O-co-doped porous carbon; accelerates LiPS conversion and suppresses shuttle | SAC-modified PP separator | 2.86 wt% Co (ICP-OES) | 1 C, 500 cycles  2 C, 1000 cycles | ≈0.047 ( 2C) | [48] |
| 20 | Co SA Array/ MOF-NS | Ultrathin MOF nanosheets containing periodically arranged Co-O₄ SA array mimic; homogeneous Li⁺ flux + suppressing dendritic growth + regulating LiPS transport | MOF-derived separator coating（LBL coated BC separator） | ≈ 24.8 wt% Co (EDX) | 1 C, 600 cycles | 0.07 | [49] |
| 21 | Sm-N₃C₃ SAC | Sm SAs coordinated in N₃C₃ sites on N-doped carbon; enables *f-d-p orbital hybridization* for bidirectional LiPS catalysis + Li dendrite suppression | SAC-modified separator  (Sm-N₃C₃@PP) | 0.98 wt% Sm (ICP-OES) | 1C, 1000 cycles  4C, 2000 cycles | 0.027 (1C)  0.0078 (4C) | [50] |
| 22 | CoN₄-CoNCNF | Atomically dispersed Co-N₄ SAs embedded in cross-linked hollow carbon nanovesicle fibers; accelerate LiPS conversion + inhibit shuttle | SAC carbon nanovesicle fiber interlayer | 1.13 wt% Co (ICP-OES) | 1 C, 1000 cycles  2 C, 5000 cycles | 0.027 (1C)  0.016 (2C) | [51] |
| (C) SAC Interlayer Systems | | | | | | | |
| 23 | CNT@CoSA (ALD) | Single-atomic Co deposited on CNT via atomic layer deposition; accelerates LiPS conversion | Free-standing CNT interlayer | 2.9 wt% Co | 1 C, 500 cycles | 0.064 | [52] |
| 24 | CoSAs/CD-CANF | Contra-diffusion grown ZIF precursor forming uniformly dispersed Co-Nₓ single-atom catalytic sites embedded in carbonized ANF nanofiber membrane; confines LiPS + excellent catalytic conversion + enhances Li⁺ transport | SAC-modified aramid nanofiber (ANF) interlayer | 1.4 wt% Co (ICP-OES)  ≈ 0.3 at% | 2 C, 2000 cycles | ≈ 0.03 | This Work |

**References**

[1] H. Pei, C. Yang, Q. Wu, X. Zhou, X. Xie, B. Hwang, Y. Ye, *Journal of Materials Chemistry A* **2022**, *10*, 5317-5327.

[2] aP. G. Bruce, C. A. Vincent, *Journal of Electroanalytical Chemistry and Interfacial Electrochemistry* **1987**, *225*, 1-17; bJ. Evans, C. A. Vincent, P. G. Bruce, *Polymer* **1987**, *28*, 2324-2328.

[3] R. S. Nicholson, I. Shain, *Analytical Chemistry* **1964**, *36*, 706-723.

[4] aW. Weppner, R. A. Huggins, *Journal of The Electrochemical Society* **1977**, *124*, 1569; bT. Wang, J. He, Z. Zhu, X.-B. Cheng, J. Zhu, B. Lu, Y. Wu, *Advanced Materials* **2023**, *35*, 2303520.

[5] aO. van der Heijden, S. Park, R. E. Vos, J. J. J. Eggebeen, M. T. M. Koper, *ACS Energy Letters* **2024**, *9*, 1871-1879; bL. Zhou, D. L. Danilov, F. Qiao, J. Wang, H. Li, R.-A. Eichel, P. H. L. Notten, *Advanced Energy Materials* **2022**, *12*, 2202094.

[6] aE. L. Cussler, *Diffusion: Mass Transfer in Fluid Systems*, Cambridge University Press, Cambridge, **2009**; bJ. Crank, *The Mathematics of Diffusion*, Oxford University Press, Oxford, **1975**.

[7] Y. Li, P. Zhou, H. Li, T. Gao, L. Zhou, Y. Zhang, N. Xiao, Z. Xia, L. Wang, Q. Zhang, L. Gu, S. Guo, *Small Methods* **2020**, *4*, 1900701.

[8] M. Zhang, S. Kong, B. Chen, M. Wu, *Batteries* **2024**, *10*, 15.

[9] H. Lin, S. Zhang, T. Zhang, S. Cao, H. Ye, Q. Yao, G. W. Zheng, J. Y. Lee, *ACS Nano* **2019**, *13*, 7073-7082.

[10] J. Liu, J. Wang, L. Zhu, X. Chen, G. Yi, Q. Ma, S. Sun, N. Wang, X. Cui, Q. Chai, J. Feng, W. Yan, *Journal of Materials Chemistry A* **2022**, *10*, 14098-14110.

[11] J. Liu, J. Wang, L. Zhu, X. Chen, Q. Ma, L. Wang, X. Wang, W. Yan, *Chemical Engineering Journal* **2021**, *411*, 128540.

[12] G. Xu, Q.-b. Yan, S. Wang, A. Kushima, P. Bai, K. Liu, X. Zhang, Z. Tang, J. Li, *Chemical Science* **2017**, *8*, 6619-6625.

[13] Y. Jiang, Y. Deng, B. Zhang, W. Hua, X. Wang, Q. Qi, Q. Lin, W. Lv, *Nanoscale* **2020**, *12*, 12308-12316.

[14] S. Deng, W. Sun, J. Tang, M. Jafarpour, F. Nüesch, J. Heier, C. Zhang, *Nano-Micro Letters* **2024**, *16*, 229.

[15] R. Yi, Y. Zhao, C. Liu, Y. Sun, C. Zhao, Y. Li, L. Yang, C. Zhao, *Nanomaterials* **2022**, *12*, 3770.

[16] J. Wang, P. Zhai, T. Zhao, M. Li, Z. Yang, H. Zhang, J. Huang, *Electrochimica Acta* **2019**, *320*, 134558.

[17] Y. Chen, L. Zhang, H. Pan, J. Zhang, S. Xiang, Z. Cheng, Z. Zhang, *Journal of Materials Chemistry A* **2021**, *9*, 26929-26938.

[18] G. Liu, Q. Zeng, Q. Wu, S. Tian, X. Sun, D. Wang, X. Li, W. Wei, T. Wu, Y. Zhang, Y. Sheng, K. Tao, E. Xie, Z. Zhang, *ACS Applied Materials & Interfaces* **2023**, *15*, 39384-39395.

[19] J. Zhang, G. Xu, Q. Zhang, X. Li, Y. Yang, L. Yang, J. Huang, G. Zhou, *Advanced Science* **2022**, *9*, 2201579.

[20] P. Guo, K. Sun, X. Shang, D. Liu, Y. Wang, Q. Liu, Y. Fu, D. He, *Small* **2019**, *15*, 1902363.

[21] P. Cheng, P. Guo, K. Sun, Y. Zhao, D. Liu, D. He, *Journal of Membrane Science* **2021**, *619*, 118780.

[22] W. Jing, J. Zu, K. Zou, X. Dai, Y. Song, J. Han, J. Sun, Q. Tan, Y. Chen, Y. Liu, *Journal of Materials Chemistry A* **2022**, *10*, 4833-4844.

[23] T. Xiao, Q. Chen, W. Zhong, M. Yang, F. Cai, W. Liu, M. Ren, Y. Wang, *Journal of Alloys and Compounds* **2022**, *907*, 164486.

[24] X. Qi, L. Huang, Y. Luo, Q. Chen, Y. Chen, *Journal of Colloid and Interface Science* **2022**, *628*, 896-910.

[25] L. Tan, X. Li, Z. Wang, H. Guo, J. Wang, *ACS Applied Materials & Interfaces* **2018**, *10*, 3707-3713.

[26] C. Shen, Y. Li, M. Gong, C. Zhou, Q. An, X. Xu, L. Mai, *ACS Appl Mater Interfaces* **2021**, *13*, 60046-60053.

[27] Y. Tian, G. Li, Y. Zhang, D. Luo, X. Wang, Y. Zhao, H. Liu, P. Ji, X. Du, J. Li, Z. Chen, *Advanced Materials* **2020**, *32*, 1904876.

[28] A. E. Shrshr, Y. Dong, M. A. Al-Tahan, X. Kang, H. Guan, X. Zheng, J. Zhang, *Journal of Alloys and Compounds* **2022**, *910*, 164917.

[29] Z. Yu, B. Wang, X. Liao, K. Zhao, Z. Yang, F. Xia, C. Sun, Z. Wang, C. Fan, J. Zhang, Y. Wang, *Advanced Energy Materials* **2020**, *10*, 2000907.

[30] M. Lu, T. Yan, Y. Ding, S. Chen, Z. Chen, J. Gu, X. Chen, L. Zhang, M. Tian, J. Sun, *Energy Storage Materials* **2024**, *70*.

[31] Z. Liang, C. Peng, J. Shen, Y. Yang, S. Yao, D. Xue, M. Zhu, J. Liu, *Journal of Power Sources* **2023**, *556*.

[32] X. Chen, Z. Zhu, E. Vargun, Y. Li, P. Saha, Q. Cheng, *Journal of Electroanalytical Chemistry* **2023**, *928*.

[33] S. Yu, Y. Sun, L. Song, X. Cao, L. Chen, X. An, X. Liu, W. Cai, T. Yao, Y. Song, W. Zhang, *Nano Energy* **2021**, *89*.

[34] Y. Li, G. Chen, J. Mou, Y. Liu, S. Xue, T. Tan, W. Zhong, Q. Deng, T. Li, J. Hu, C. Yang, K. Huang, M. Liu, *Energy Storage Materials* **2020**, *28*, 196-204.

[35] Y. Li, J. Wu, B. Zhang, W. Wang, G. Zhang, Z. W. Seh, N. Zhang, J. Sun, L. Huang, J. Jiang, J. Zhou, Y. Sun, *Energy Storage Materials* **2020**, *30*, 250-259.

[36] Q. Lv, Y. Sun, B. Li, C. Li, Q. Zhang, L. Wang, *Advanced Energy Materials* **2024**, *15*.

[37] H. Yang, L. Wang, C. Geng, Y. Zhao, Q. Li, X. Jiang, Z. Tian, M. Wang, C. Jiang, Z. Sun, B. Cui, Y. B. He, W. Chen, W. Lv, Q. H. Yang, *Advanced Energy Materials* **2024**, *14*.

[38] C. Dong, C. Ma, C. Zhou, Y. Yu, J. Wang, K. Yu, C. Shen, J. Gu, K. Yan, A. Zheng, M. Gong, X. Xu, L. Mai, *Adv Mater* **2024**, *n/a*, e2407070.

[39] C. Wang, H. Song, C. Yu, Z. Ullah, Z. Guan, R. Chu, Y. Zhang, L. Zhao, Q. Li, L. Liu, *Journal of Materials Chemistry A* **2020**, *8*, 3421-3430.

[40] Y. Zhang, C. Kang, W. Zhao, Y. Song, J. Zhu, H. Huo, Y. Ma, C. Du, P. Zuo, S. Lou, G. Yin, *Journal of the American Chemical Society* **2023**, *145*, 1728-1739.

[41] G. Zhao, Q. Chen, L. Wang, T. Yan, H. Li, C. Yuan, J. Mao, X. Feng, D. Sun, L. Zhang, *Journal of Materials Chemistry A* **2022**, *10*, 19893-19902.

[42] S. Zhang, X. Ao, J. Huang, B. Wei, Y. Zhai, D. Zhai, W. Deng, C. Su, D. Wang, Y. Li, *Nano Letters* **2021**, *21*, 9691-9698.

[43] Y. Wang, C. Shi, J. Sha, L. Ma, E. Liu, N. Zhao, *ACS Applied Materials & Interfaces* **2022**, *14*, 25337-25347.

[44] K. Zhang, Z. Chen, R. Ning, S. Xi, W. Tang, Y. Du, C. Liu, Z. Ren, X. Chi, M. Bai, C. Shen, X. Li, X. Wang, X. Zhao, K. Leng, S. J. Pennycook, H. Li, H. Xu, K. P. Loh, K. Xie, *ACS Applied Materials & Interfaces* **2019**, *11*, 25147-25154.

[45] W. Cao, M. Liu, K. Zhang, S. Zhang, C. Li, Y. Wen, Q. Lv, Q. Wen, L. Wang, *J Colloid Interface Sci* **2025**, *684*, 189-196.

[46] Z. Lian, L. Ma, H. Wu, H. Xiao, Y. Yang, J. Zhang, J. Zi, X. Chen, W. Wang, H. Li, *Applied Catalysis B: Environment and Energy* **2025**, *361*.

[47] S. He, J. Yang, S. Liu, X. Wang, X. Che, M. Wang, J. Qiu, *Chemical Engineering Journal* **2023**, *454*.

[48] Y. Zhang, H. Yuan, E. Guo, S. Chen, M. Ren, J. Ma, J. Cui, M. Li, L. Jing, L. Li, *Journal of Energy Chemistry* **2024**, *99*, 604-614.

[49] Y. Li, S. Lin, D. Wang, T. Gao, J. Song, P. Zhou, Z. Xu, Z. Yang, N. Xiao, S. Guo, *Adv Mater* **2020**, *32*, e1906722.

[50] R. Zhou, Y. Ren, W. Li, M. Guo, Y. Wang, H. Chang, X. Zhao, W. Hu, G. Zhou, S. Gu, *Angew Chem Int Ed Engl* **2024**, *63*, e202405417.

[51] H. Zhuang, T. Zhang, H. Xiao, F. Zhang, P. Han, H. Gu, J. Jiao, W. Chen, Q. Gao, *Applied Catalysis B: Environmental* **2024**, *340*.

[52] Q. Lin, B. Ding, S. Chen, P. Li, Z. Li, Y. Shi, H. Dou, X. Zhang, *ACS Applied Energy Materials* **2020**, *3*, 11206-11212.
